# Supplementary material for: Immune or Genetic-Mediated Disruption of CASPR2 Causes Pain Hypersensitivity Due to Enhanced Primary Afferent Excitability
Source: Neuron. 2018 Feb 21;97(4):806–822.e10. doi: 10.1016/j.neuron.2018.01.033 (PMC6011627; doi:10.1016/j.neuron.2018.01.033)
Supplement: Document S1. Figures S1–S10, Tables S1–S8, and Data S1 [file mmc1.pdf]

Neuron, Volume *97*

Supplemental Information

Immune or Genetic-Mediated Disruption of CASPR2 Causes Pain Hypersensitivity Due to Enhanced Primary Afferent Excitability

John M. Dawes, Greg A. Weir, Steven J. Middleton, Ryan Patel, Kim I. Chisholm, Philippa Pettingill, Liam J. Peck, Joseph Sheridan, Akila Shakir, Leslie Jacobson, Maria Gutierrez-Mecinas, Jorge Galino, Jan Walcher, Johannes Kühnemund, Hannah Kuehn, Maria D. Sanna, Bethan Lang, Alex J. Clark, Andreas C. Themistocleous, Noboru Iwagaki, Steven J. West, Karolina Werynska, Liam Carroll, Teodora Trendafilova, David A. Menassa, Maria Pia Giannoccaro, Ester Coutinho, Ilaria Cervellini, Damini Tewari, Camilla Buckley, M. Isabel Leite, Hendrik Wildner, Hanns Ulrich Zeilhofer, Elior Peles, Andrew J. Todd, Stephen B. McMahon, Anthony H. Dickenson, Gary R. Lewin, Angela Vincent, and David L. Bennett

Figure S1

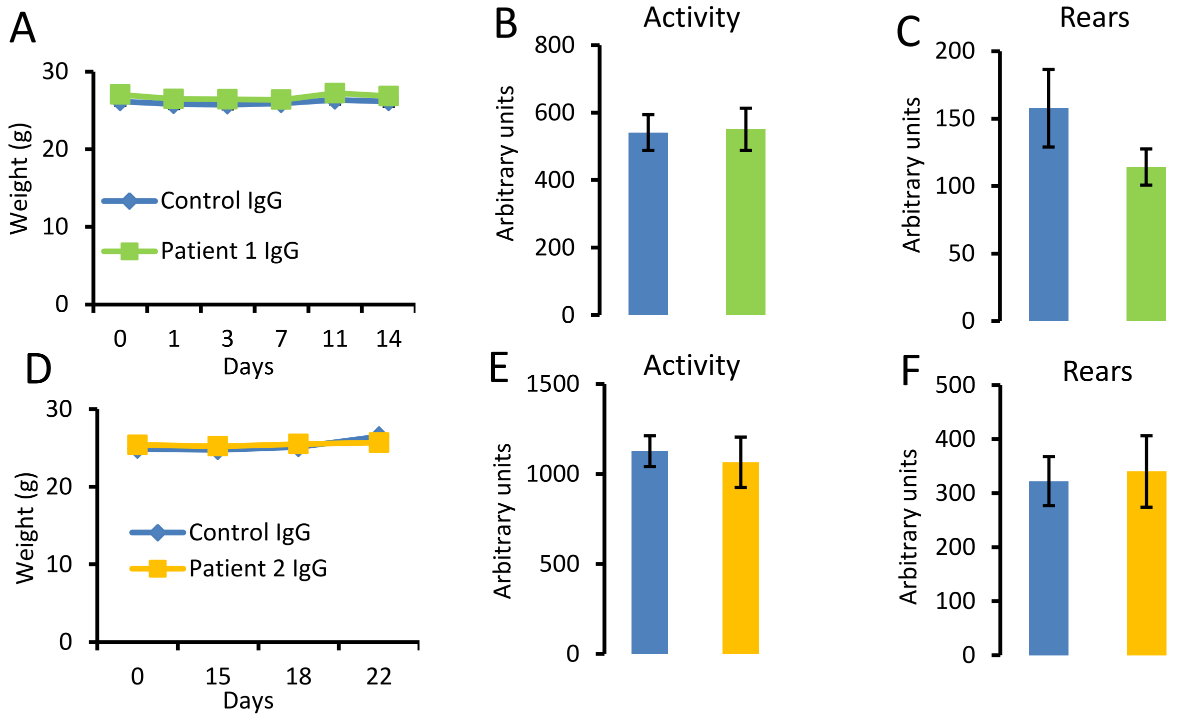

A
30
20
10
0
Weight (g)
Control IgG
Patient 1 IgG
0
1
3
7
11
14
Days
B
Activity
800
600
400
200
0
Arbitrary units
C
Rears
200
150
100
50
0
Arbitrary units
D
30
20
10
0
Weight (g)
Control IgG
Patient 2 IgG
0
15
18
22
Days
E
Activity
1500
1000
500
0
Arbitrary units
F
Rears
500
400
300
200
100
0
Arbitrary units

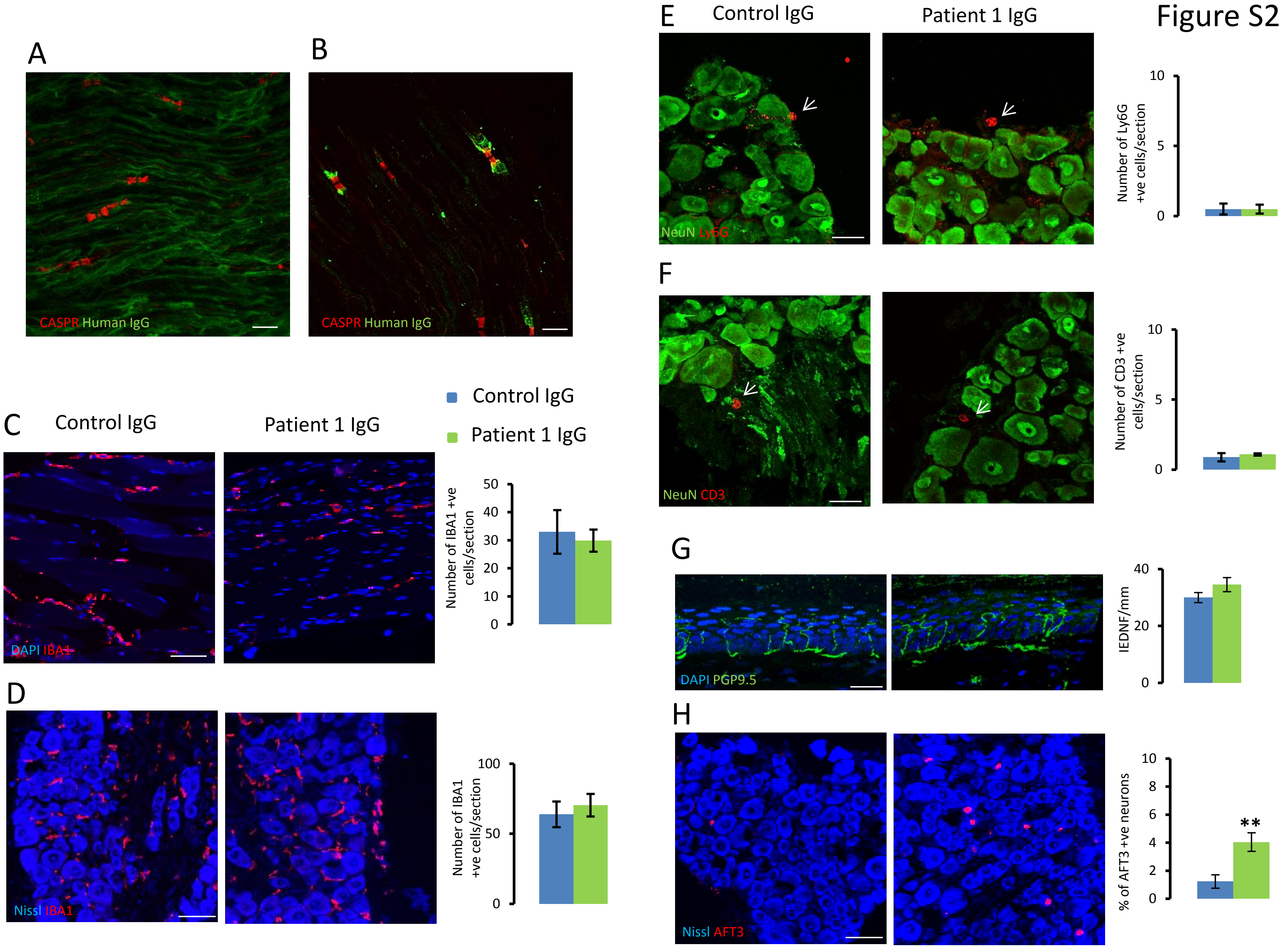
Figure S2
A
B
CASPR Human IgG
CASPR Human IgG
C
Control IgG
Patient 1 IgG
Control IgG
Patient 1 IgG
DAPI IBA1
Number of IBA1 +ve cells/section
50
40
30
20
10
0
D
Nissl IBA1
Number of IBA1 +ve cells/section
100
50
0
E
Control IgG
Patient 1 IgG
NeuN Ly6G
Number of Ly6G +ve cells/section
10
5
0
F
NeuN CD3
Number of CD3 +ve cells/section
10
5
0
G
DAPI PGP9.5
IEDNF/mm
40
20
0
H
Nissl AFT3
% of AFT3 +ve neurons
10
8
6
4
2
0
**

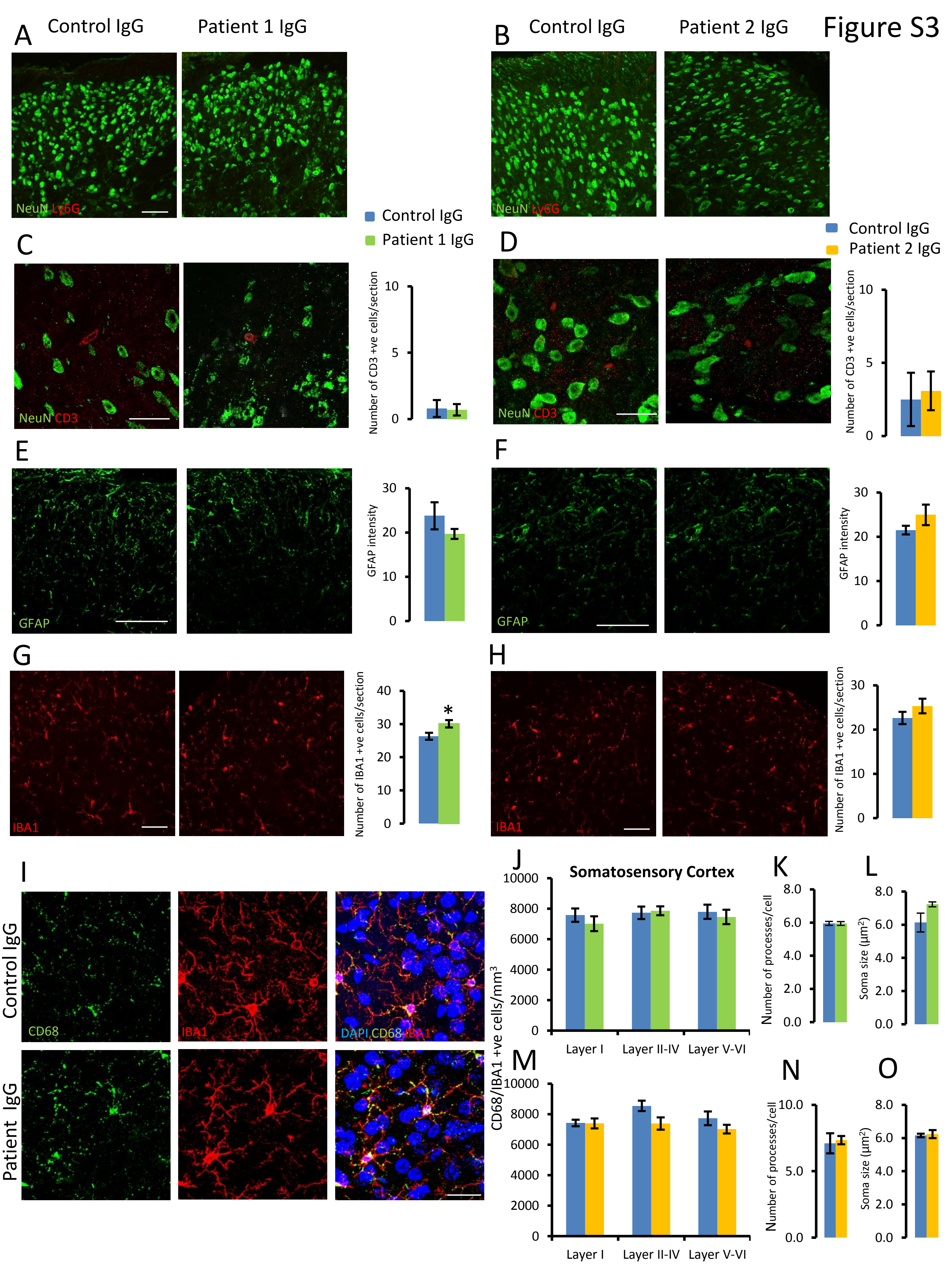

Figure S3
A
Control IgG
Patient 1 IgG
NeuN Ly6G
B
Control IgG
Patient 2 IgG
NeuN Ly6G
C
Control IgG
Patient 1 IgG
NeuN CD3
Number of CD3 +ve cells/section
D
Control IgG
Patient 2 IgG
NeuN CD3
Number of CD3 +ve cells/section
E
GFAP
GFAP intensity
F
GFAP
GFAP intensity
G
IBA1
Number of IBA1 +ve cells/section
*
H
IBA1
Number of IBA1 +ve cells/section
I
Control IgG
Patient IgG
CD68
IBA1
DAPI CD68 IBA1
J
Somatosensory Cortex
CD68/IBA1 +ve cells/mm3
Layer I
Layer II-IV
Layer V-VI
K
Number of processes/cell
L
Soma size (µm2)
M
Layer I
Layer II-IV
Layer V-VI
N
Number of processes/cell
O
Soma size (µm2)

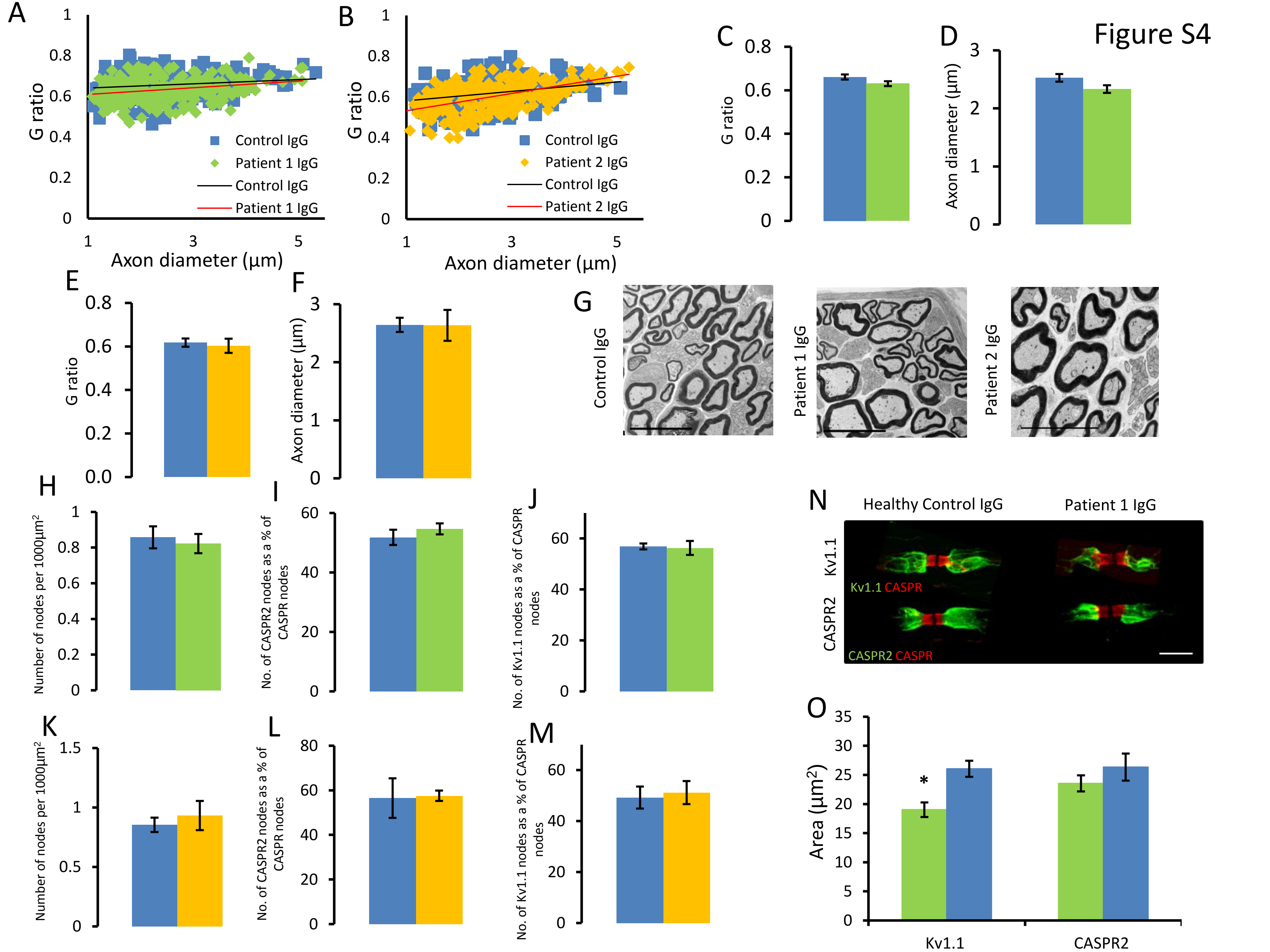

Figure S4
A
G ratio
Axon diameter (µm)
Control IgG
Patient 1 IgG
Control IgG
Patient 1 IgG
B
G ratio
Axon diameter (µm)
Control IgG
Patient 2 IgG
Control IgG
Patient 2 IgG
C
G ratio
D
Axon diameter (µm)
E
G ratio
F
Axon diameter (µm)
G
Control IgG
Patient 1 IgG
Patient 2 IgG
H
Number of nodes per 1000µm²
I
No. of CASPR2 nodes as a % of CASPR nodes
J
No. of Kv1.1 nodes as a % of CASPR nodes
N
Healthy Control IgG
Patient 1 IgG
Kv1.1
CASPR2
Kv1.1 CASPR
CASPR2 CASPR
K
Number of nodes per 1000µm²
L
No. of CASPR2 nodes as a % of CASPR nodes
M
No. of Kv1.1 nodes as a % of CASPR nodes
O
Area (µm²)
Kv1.1
CASPR2

Figure S5

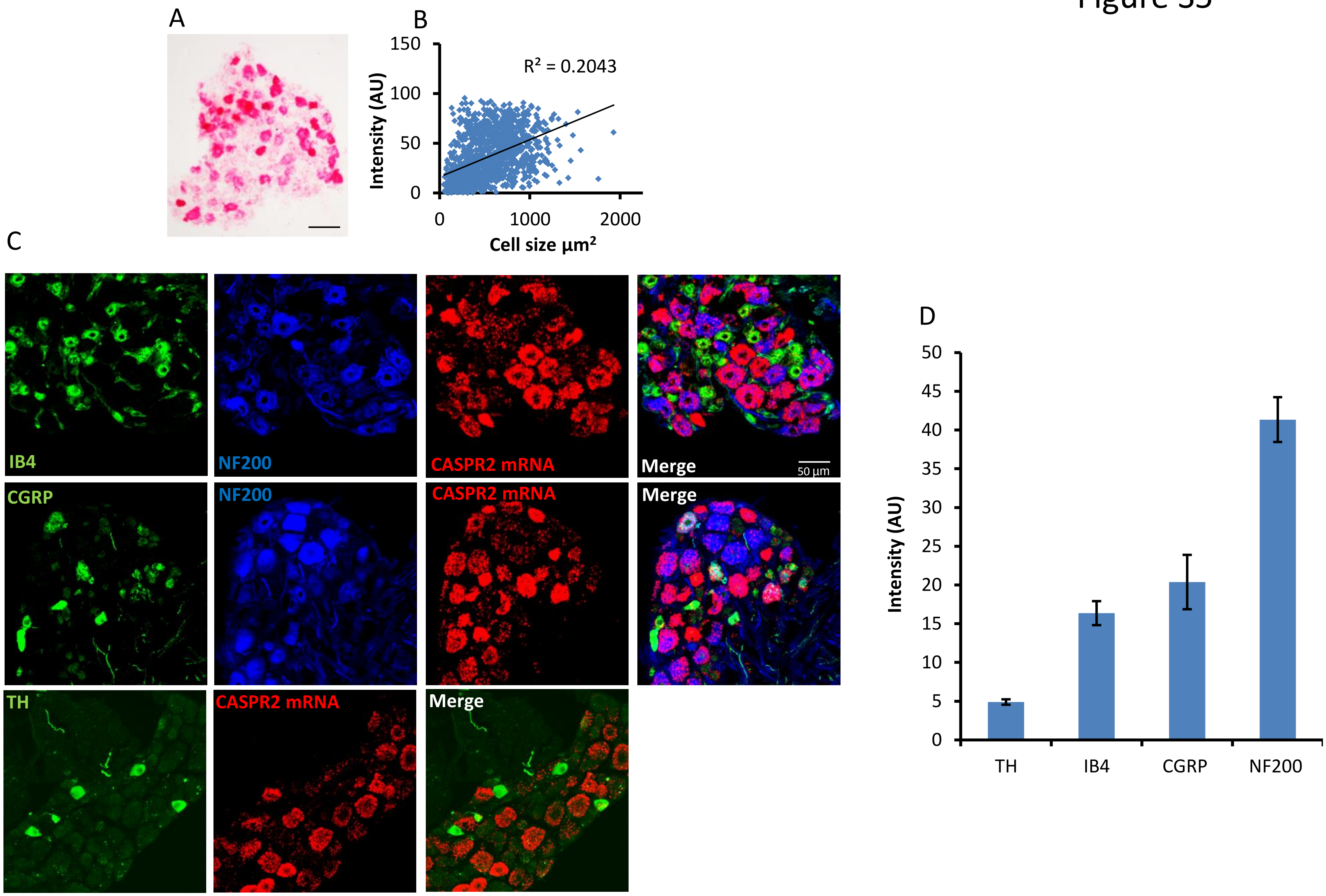
A
B
150
100
50
0
Intensity (AU)
R² = 0.2043
0
1000
2000
Cell size µm²
C
IB4
NF200
CASPR2 mRNA
Merge
50 µm
CGRP
NF200
CASPR2 mRNA
Merge
TH
CASPR2 mRNA
Merge
D
50
45
40
35
30
25
20
15
10
5
0
Intensity (AU)
TH
IB4
CGRP
NF200

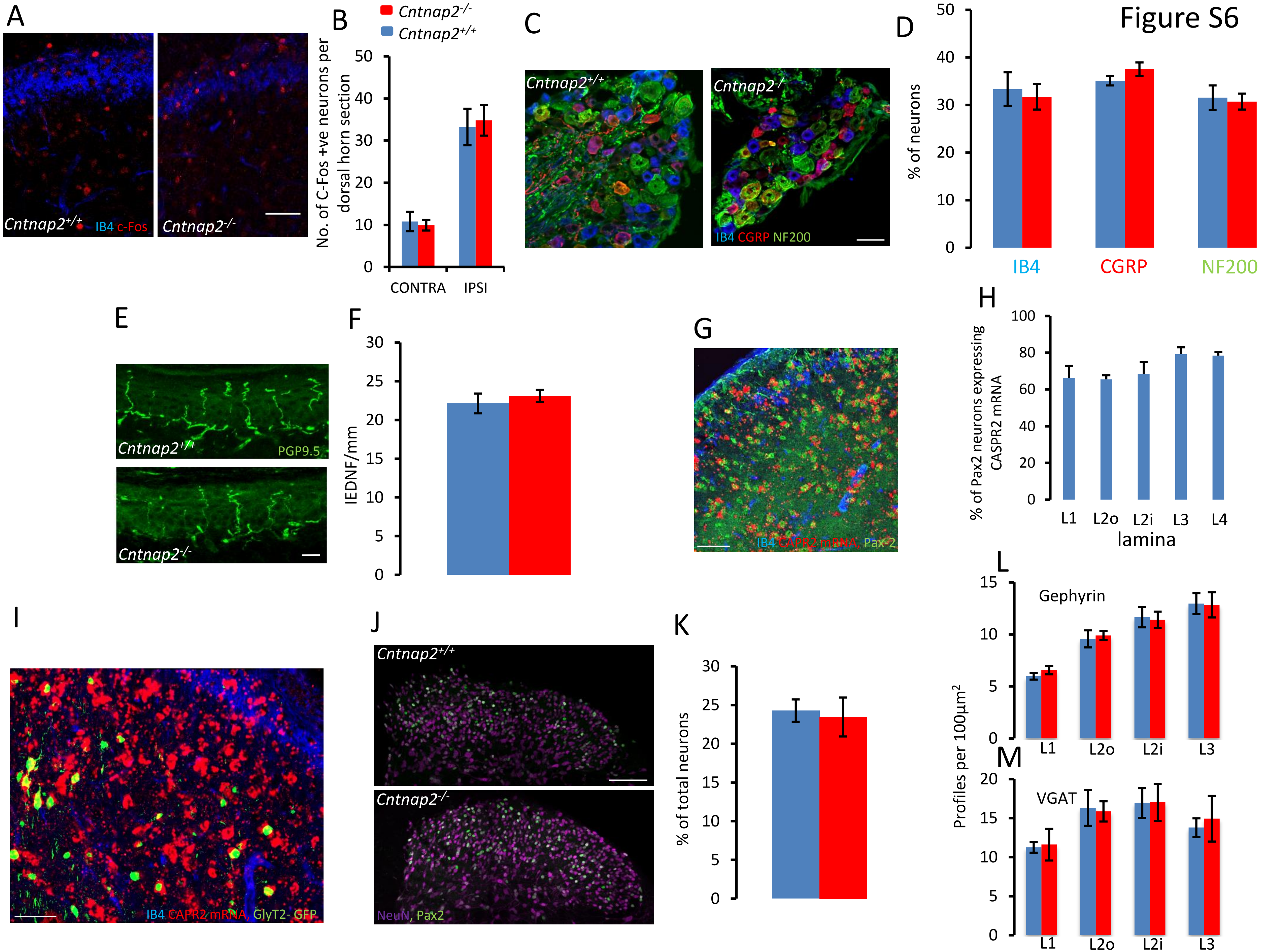
Figure S6
A
Cntnap2+/+
IB4 c-Fos
Cntnap2-/-
B
Cntnap2-/-
Cntnap2+/+
No. of C-Fos +ve neurons per dorsal horn section
CONTRA
IPSI
C
Cntnap2+/+
Cntnap2-/-
IB4 CGRP NF200
D
% of neurons
IB4
CGRP
NF200
E
Cntnap2+/+
PGP9.5
Cntnap2-/-
F
IEDNF/mm
G
IB4 CAPR2 mRNA, Pax-2
H
% of Pax2 neurons expressing CASPR2 mRNA
L1
L2o
L2i
L3
L4
lamina
I
IB4 CAPR2 mRNA, GlyT2- GFP
J
Cntnap2+/+
Cntnap2-/-
NeuN, Pax2
K
% of total neurons
L
Gephyrin
M
VGAT
Profiles per 100µm2

Figure S7

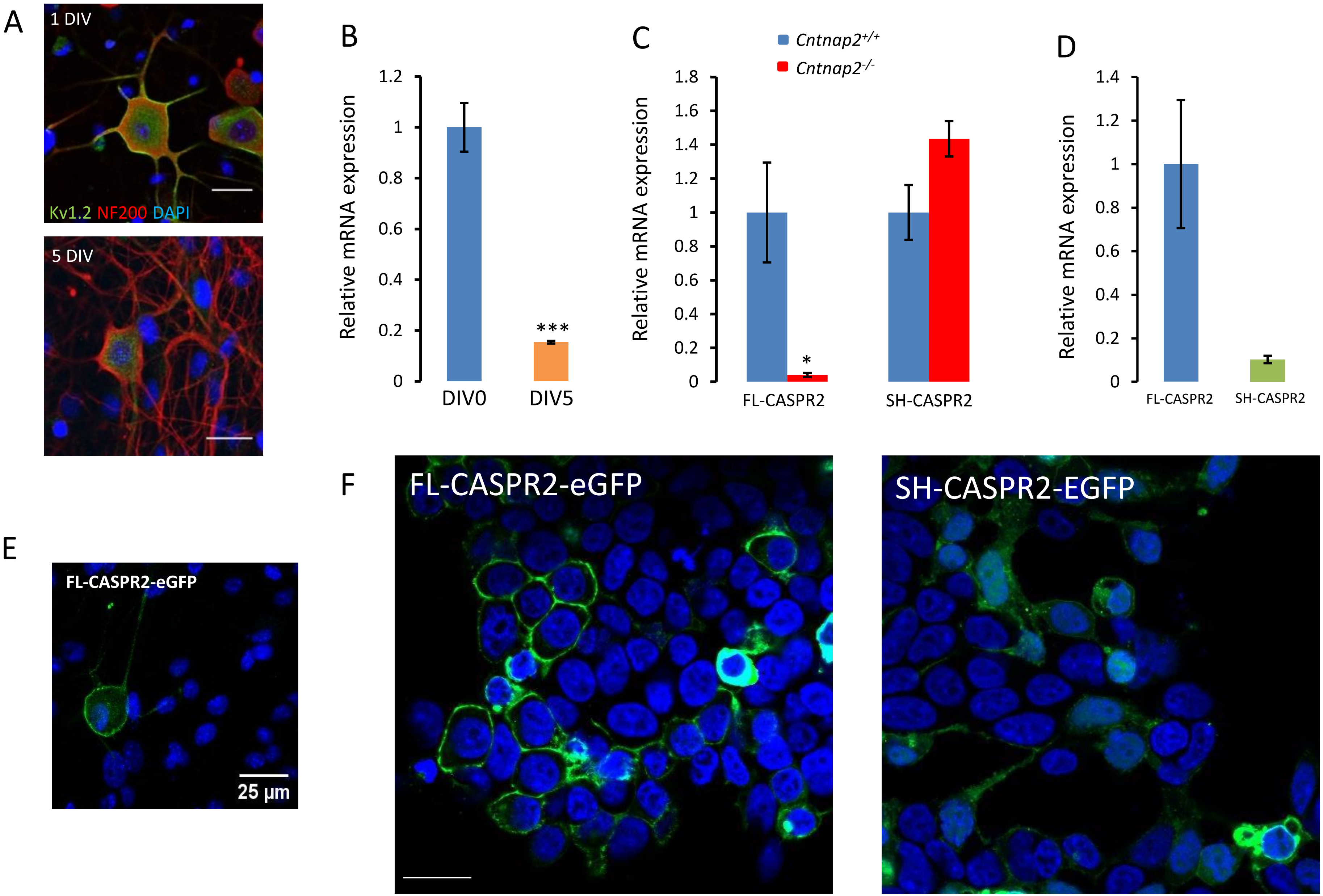

A
1 DIV
Kv1.2 NF200 DAPI
5 DIV
B
Relative mRNA expression
1.2
1
0.8
0.6
0.4
0.2
0
***
DIV0
DIV5
C
Cntnap2+/+
Cntnap2-/-
Relative mRNA expression
1.8
1.6
1.4
1.2
1
0.8
0.6
0.4
0.2
0
*
FL-CASPR2
SH-CASPR2
D
Relative mRNA expression
1.4
1.2
1
0.8
0.6
0.4
0.2
0
FL-CASPR2
SH-CASPR2
E
FL-CASPR2-eGFP
25 µm
F
FL-CASPR2-eGFP
SH-CASPR2-EGFP

Figure S8

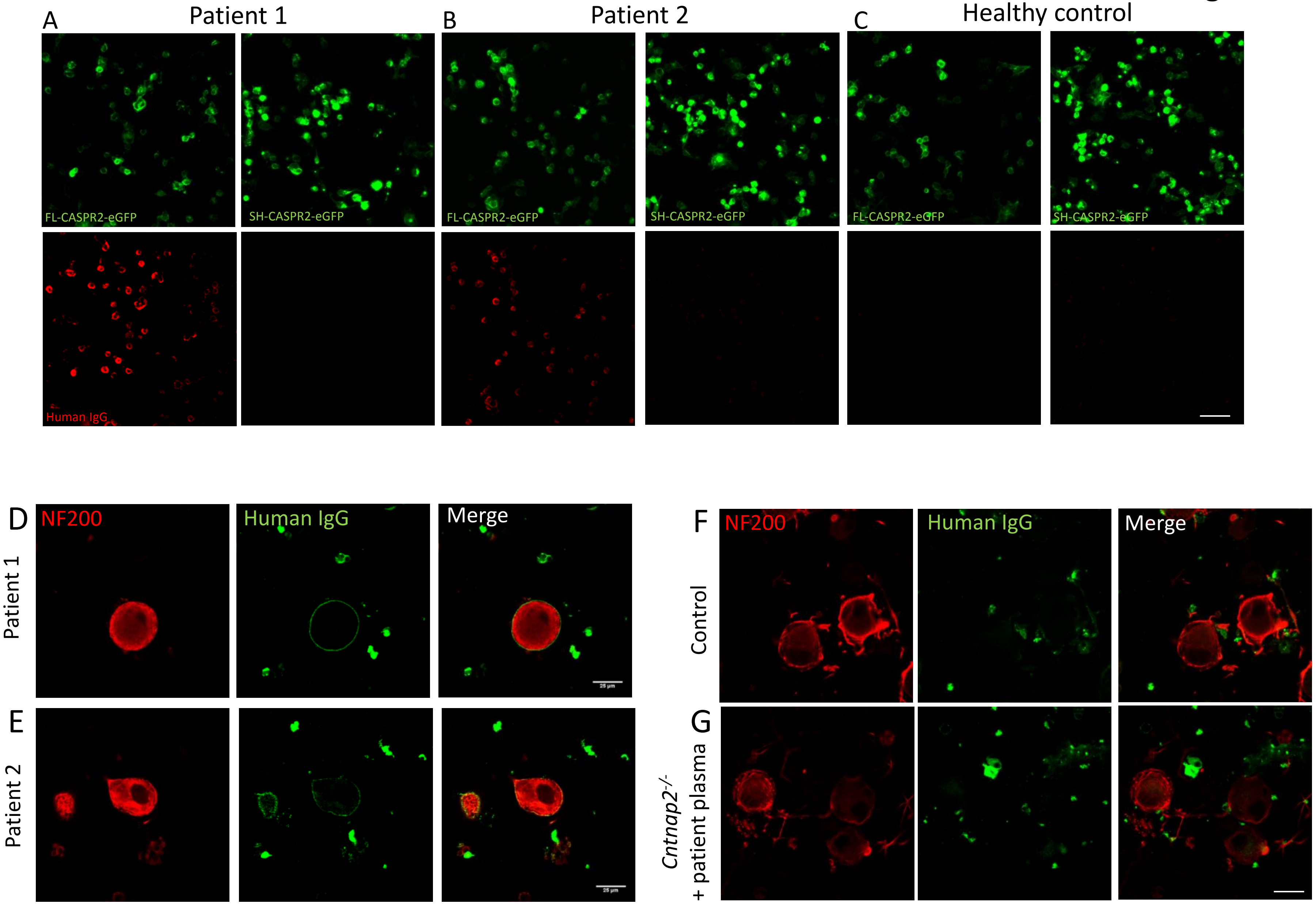

A
Patient 1
B
Patient 2
C
Healthy control
FL-CASPR2-eGFP
SH-CASPR2-eGFP
FL-CASPR2-eGFP
SH-CASPR2-eGFP
FL-CASPR2-eGFP
SH-CASPR2-eGFP
Human IgG
D
NF200
Human IgG
Merge
Patient 1
E
Patient 2
F
NF200
Human IgG
Merge
Control
G
Cntnap2$^{-/-}$
+ patient plasma

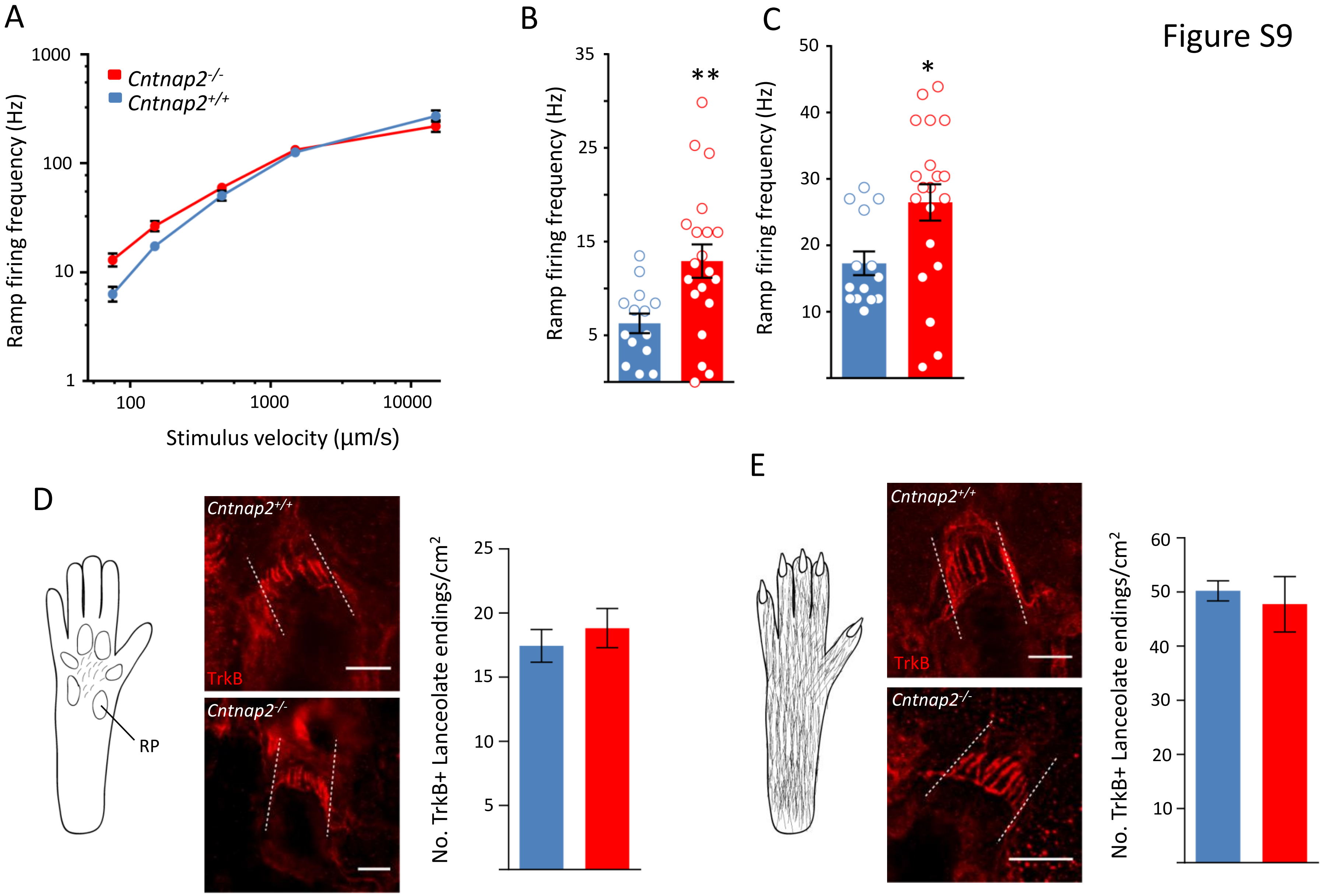

A
B
C
Figure S9
Cntnap2-/-
Cntnap2+/+
Ramp firing frequency (Hz)
1000
100
10
1
100
1000
10000
Stimulus velocity (µm/s)
35
25
15
5
**
50
40
30
20
10
*
D
E
RP
Cntnap2+/+
Cntnap2-/-
TrkB
No. TrkB+ Lanceolate endings/cm2
25
20
15
10
5
60
50
40
30
20
10

Figure S10

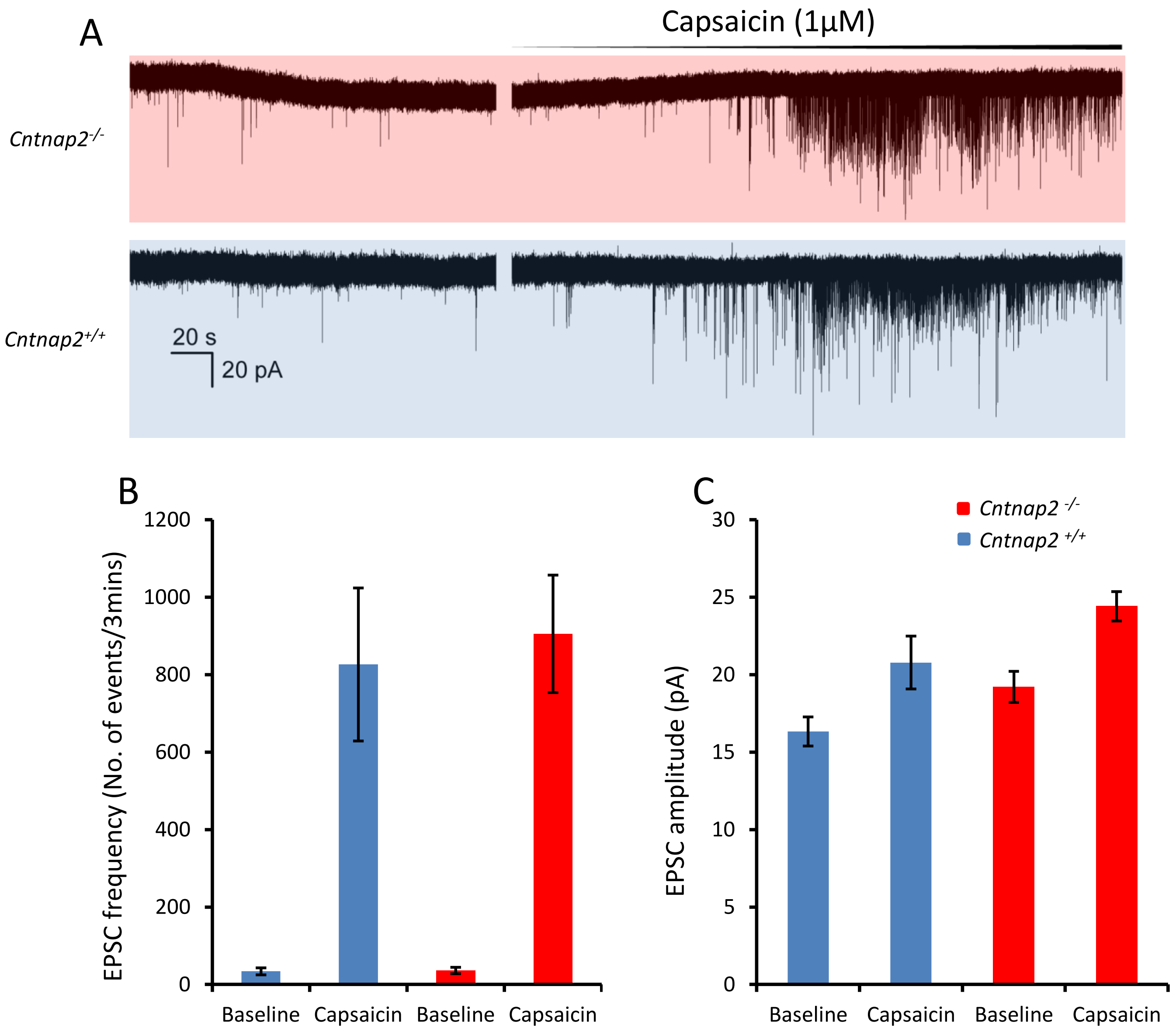
A
Capsaicin (1μM)
Cntnap2-/-
Cntnap2+/+
20 s
20 pA
B
EPSC frequency (No. of events/3mins)
1200
1000
800
600
400
200
0
Baseline
Capsaicin
Baseline
Capsaicin
C
Cntnap2 -/-
Cntnap2 +/+
EPSC amplitude (pA)
30
25
20
15
10
5
0
Baseline
Capsaicin
Baseline
Capsaicin

Supplementary Figure 1 Mice treated with patient IgG do not develop sickness and display normal motor activity. Related to Figure 1

(A and D) Weight measurements over time for mice treated with IgG from CASPR2-Ab positive patients or healthy control.

(B and E) Locomotor activity was assessed using the open field test in mice treated with IgG from CASPR2-Ab positive patients or healthy control. An area under the curve analysis of the number of boxes entered within a 3 minute period was used to incorporate data taken from multiple time points across the dosing regime.

(C and F) Rearing behaviour measured during the open field test, data shown as area under the curve to incorporate data taken from multiple time points across the dosing regime.

For A-C, n=8, for D-F n=9. All data shown as mean ± SEM

Supplementary Figure 2 Passive transfer of patient CASPR2-Abs does not cause gross inflammation or damage to the mouse peripheral nerve. Related to Figure 2

(A) Sciatic nerve section from a mouse treated in vivo with Patient IgG. CASPR (red) is used to mark the paranode and there is no specific binding to nodal regions of human IgG (green). Scale bar 10µm.

(B) Sciatic nerve section taken from WT mouse, permeabilised with Triton-X to allow access to the JXP and subsequently treated with Patient IgG. Clear binding of human IgG to the JXP can be seen using IgG from a CASPR2-Ab positive patient. Scale bar 10µm.

(C and D) Representative images of sciatic nerve (C) and DRG (D) sections from mice treated with either control or Patient 1 IgG. Nissl or DAPI in blue, the macrophage marker IBA1 shown in red. Quantification shows no difference between the numbers of IBA1 positive cells between treatment groups. Data shown as the average number of IBA1 positive cells per image, 3 images per animal, n=5. Scale bar 50µm

(E and F) Representative images of DRG sections from mice treated with either control or Patient 1 IgG stained for the neutrophil marker Ly6G (red, E) and the T cell marker CD3 (red, F). The number of positive cells was very low and no difference was found between groups. Data shown as the average number of cells per DRG section, 3-4 images per animal, n=4. Scale bar 25 µm.

(G) Representative images of mouse glabrous skin. PGP9.5 (green) was used to mark nerve fibres, DAPI shown in blue. Quantification showed no difference in the number of IEDNFs between treatment groups, 3 images per animal, n=5. Scale bar 25µm

(H) Representative images of mouse DRG sections immunostained for the injury marker ATF3 (red). Quantification showed a small but significant increase in ATF3 positive neurons in those mice treated with patient 1 IgG versus controls. The number of ATF3 positive cells shown as a percentage of nissl (blue) positive neurons, 3-4 images per animal, n=5. Scale bar 50µm.

For H Students t test, $^{**}p<0.01$ versus Control IgG group. All data shown as mean ± SEM

Supplementary Figure 3 Passive transfer of patient CASPR2-Abs does not cause a cellular inflammatory response in the CNS. Related to Figure 2

(A and B) Representative images of spinal cord stained for the neutrophil marker Ly6G (red) from mice treated with either control, Patient 1 (A) or Patient 2 IgG (B). No neutrophils were detected in the spinal cord, scale bar 50 µm.

(C and D) Very few CD3 (red) positive cells (a marker of T cells) were detected in spinal cord from control or Patient IgG treated mice (C and D, Patient 1 and 2 IgG groups respectively). No difference was found in the number of T cells between treatment groups. Data shown as the average number of CD3 positive cells per section, 3-4 images per animal, n=4. Scale bar 25 µm.

(E and F) Representative images of GFAP (green) intensity, used as an indicator of astrocyte reactivity, in spinal cord sections from mice treated with control versus Patient 1 (E) and Patient 2 IgG (F). No significant difference was seen between treatment groups. Data shown as the average intensity of GFAP staining per section, 3-4 images per animal, n=4. Scale bar 50 µm.

(G and H) Representative images of microglia, stained using the marker IBA1 (red), in spinal cord sections taken from mice treated with control versus Patient 1 (G) or Patient 2 (H) IgG. Quantification shows a small but significant increase in microglia number in the spinal cord from mice treated with Patient 1 IgG versus control. No difference was seen for Patient 2 IgG. Data shown as the average number of IBA1 positive cells per section, 3-4 images per animal, n=4. Scale bar 50 µm.

(I) Representative images of CD68 (a marker of microglia activation, green) and IBA1 (red) colocalisation in the somatosensory cortex of mice treated with control or Patient IgG. DAPI is shown in blue, scale bar 20 µm.

(J and M) No difference was found in the density of CD68/IBA1 positive microglia in layer I, Layers II-IV and Layers V-VI of the somatosensory cortex in mice treated with Patient 1 (J) or Patient 2 IgG (M) versus control, n=4.

(K, L, N & O) Morphological analysis of microglia showed no difference between treatment groups. There was no difference in the number of processes per cell between microglia in control mice and mice treated with either Patient 1 (K) or Patient 2 (N) IgG. And there was no difference in the soma size of microglia in control versus Patient 1 (L) or Patient 2 (O) treated mice.

For G Students t test, *p<0.05 versus Control IgG group. All data shown as mean ± SEM

Supplementary Figure 4 Analysis of peripheral nerve ultrastructure and nodes in mice treated with patient CASPR2-Abs. Related to Figure 2

(A&B) Scatter plots showing the G ratio plotted against axon diameter of individual axons from sural nerve taken from mice treated with either Patient 1 IgG (A) or Patient 2 IgG (B) versus control IgG, n=3, > 250 axons counts per group.

(C-F) on average no difference was seen for G ratios (C&E) or the axon diameter (D&F) between groups treated with either patient or control IgG, n=3 mice per group.

(G) Representative EM images of sural nerve from mice treated with Patient 1, Patient 2 or control IgG. Scale bar 10µm.

(H and K) Quantification of the number of bilateral CASPR positive nodes between mice treated with patient or control IgG. Data shown as number of nodes per 1000μm^2 area.

(I and L) Quantification of the number of CASPR2 positive nodes as a percentage of nodes with CASPR staining between treatment groups.

(J and M) Quantification of the number of Kv1.1 positive nodes as a percentage of nodes with CASPR staining between treatment groups.

(N) High power representative images of single nodes as marked by CASPR (red) from the mouse sciatic nerve. Kv1.1 (green, top) and CASPR2 (green, bottom) staining is reduced in mice treated with patient 1 IgG. Scale bar 10µm

(O) Quantification shows a significant reduction in the area of Kv1.1 immunostaining in the patient 1 IgG group versus control.

(H-J and O) n=5 animals, (K-M) n=4 animals. 3-6 images per animal.

For O Students t test , *p<0.05 versus control IgG group. All data shown as mean ± SEM

Supplementary Figure 5 CASPR2 expression in DRG neurons. Related to Figure 3

(A) Light microscope image of ISH for CASPR2 mRNA in WT L4 mouse DRG. Scale bar 100µm.
(B) Correlation between the intensity of CASPR2 mRNA signal versus DRG neuron cell size. Data obtained from L4 and 5 DRG from WT mice (n=3). A total of 1437 cells were analysed. Individual data points shown.
(C) Combined immunofluorescence with ISH for DRG neuron markers (IB4, CGRP, NF200 and TH) and CASPR2 mRNA.
(D) Average intensity of CASPR2 mRNA signal in different populations of DRG neurons from WT mouse L4 and 5 DRG (3-4 images per animal, n=3). Data shown as mean ±SEM

Supplementary Figure 6 No gross anatomical changes found within the sensory nervous system of *Cntnap2$^{-/-}$* mice. Related to Figure 3

(A) Representative images of spinal cord from both *Cntnap2$^{+/+}$* and *Cntnap2$^{-/-}$* mice taken ipsilateral to formalin (5%) injection into the hind paw stained with the neuronal activity marker c-fos (red). IB4 binding shown in blue. Scale bar 50µm.

(B) Quantification shows a clear increase in the number of c-fos positive cells in the ipsilateral dorsal horn versus contralateral following formalin injection. However no difference was seen between genotypes. Data shown as the average of 5 images per animal, *Cntnap2$^{+/+}$* (n=5) and *Cntnap2$^{-/-}$* (n=6).

(C) Representative images of DRG from both *Cntnap2$^{+/+}$* and *Cntnap2$^{-/-}$* mice, immunostained for the sensory neuronal markers IB4 (blue), CGRP (red) and NF200 (green). Scale bar 50µm

(D) Quantification of sensory neurons expressing IB4, CGRP and NF200. No difference was seen between genotypes. Data shown as the average of 2-4 images per animal, *Cntnap2$^{+/+}$* (n=4) and *Cntnap2$^{-/-}$* (n=5).

(E) Representative images of mouse glabrous skin immunostained for PGP9.5 to mark free nerve endings in the epidermis. Scale bar 25µm

(F) Quantification of IEDNF showed no differences between genotypes (3 images per animal, n=6 for both genotypes).

(G) Representative image of a WT dorsal horn of the spinal cord showing immunostaining for Pax2 (green), binding of IB4 (blue), and ISH for CASPR2 mRNA (red). Scale bar 50µm.

(H) Quantification of the percentage of Pax2 positive neurons that also express CASPR2 mRNA from different lamina within the dorsal horn of the spinal cord of WT mice (3-5 images per animal, n=3).

(I) Image from Glycine transporter 2-GFP reporter mice showing CASPR2 mRNA (red) expression in inhibitory interneurons in deep dorsal horn. IB4 binding shown in blue. Scale bar 50µm.

(J) Representative images of the dorsal horn of the mouse spinal cord. Immunostaining for the pan-neuronal marker NeuN (purple) and the inhibitory interneuron marker Pax2 (green) is shown. Scale bar 100µm

(K) Quantification of the percentage of Pax2 immunoreactive neurons shows no difference between genotypes, n=4.

(L and M) Quantification of gephyrin (a postsynaptic marker of inhibitory synapses, L) or VGAT (presynaptic marker of inhibitory synapses, M) positive puncta in *Cntnap2*$^{+/+}$ and *Cntnap2*$^{-/-}$ mice (n=4).

All data shown as mean ±SEM

Supplementary Figure 7 Expression of FL- and SH-CASPR2 mRNA in mouse DRG and overexpression *in vitro*. Related to Figure 5

(A) Representative image of Kv1.2 expression (green) in cultured WT DRG neurons after 1 and 5 days in vitro (DIV). NF200 in red, DAPI in blue. Scale bar 25µm.

(B) qPCR showing that CASPR2 mRNA expression is significantly reduced in mouse DRG neurons after 5 DIV, n=3 independent cultures.

(C) qPCR showing that the FL-CASPR2 is lost in DRG taken from *Cntnap2*$^{-/-}$. The mRNA for SH-CASPR2 is still expressed, n=4 per genotype.

(D) In lumbar DRG from WT mice, the mRNA of FL-CASPR2 is more greatly expressed (~10 times) than the shorter isoform, n=4.

(E) Representative image showing membrane localisation of the FL-CASPR2 tagged with eGFP in cultured mouse DRG neurons.

(F) Representative images of HEK cells transfected with plasmids containing either FL-CASPR2 or SH-CASPR2 tagged with eGFP. Membrane localisation of eGFP seen in the HEK cells transfected with FL-CASPR2, but not the shorter isoform. Scale bar 25µm.

For B and C Students t test, *p<0.05, ***p<0.001 versus either DIV0 (B) or *Cntnap2*$^{+/+}$ group (C). All data shown as mean ±SEM.

Supplementary Figure 8 Patient CASPR2-Abs bind WT mouse DRG neurons, but do not bind to the short CASPR2 isoform or DRG neurons from *Cntnap2*$^{-/-}$ mice. Related to Figure 5 and 8

(A-C) CBA using HEK cells overexpressing either FL-CASPR2 or SH-CASPR2 tagged to eGFP. Immunoreactivity for human IgG (red) shown only on cells overexpressing FL-CASPR2, but not SH-CASPR2 when treated with both patient 1 and patient 2 plasma (A and B). No human IgG immunostaining was seen in HEK cells overexpressing either isoform of CASPR2 for the healthy control plasma (C). Scale bar 50µm.

(D and E) Cultured mouse WT DRG neurons show membrane immunostaining for human IgG (green) following treatment of live cells with both Patient 1 (A) and Patient 2 (B) plasma. These cells were mainly NF200 positive (red).

(F) No binding of IgG from healthy control plasma on DRG neurons

(G) Patient IgG from plasma does not bind to cultured DRG neurons from *Cntnap2*$^{-/-}$ mice. Scale bar 25µm.

Supplementary Figure 9 Genetic ablation of FL-CASPR2 causes D-hair hyperexcitability at slow velocities with no anatomical changes at the lanceolate endings. Related to Figure 6

(A) The stimulus response curves of both *Cntnap2*$^{+/+}$ and *Cntnap2*$^{-/-}$ D-hairs to a large range of increasing stimulus velocities (*Cntnap2*$^{+/+}$ n=14, *Cntnap2*$^{-/-}$ n=20 units recorded from 16 *Cntnap2*$^{+/+}$ and 15 *Cntnap2*$^{-/-}$ mice).

(B&C) Ramp firing frequency was significantly increased at stimulation velocities of 75µm/s (B) and 150µm/s (C) (where each point is a single recording), indicating that D-hairs show significant hyperexcitability compared to *Cntnap2*$^{+/+}$ controls.

(D&E) Using whole-mount IHC, TrkB+ Lanceolate endings were visualised and quantified in both hind paw glabrous (D) and hind paw hairy (E) skin. There was no differences in the number of TrkB+ D-hairs in *Cntnap2*$^{-/-}$ or *Cntnap2*$^{+/+}$ mice in either skin type. White dashed lines denote hair follicle orientation, scale bar 20µm, (*Cntnap2*$^{-/-}$ n=3 animals, (D) 87 TrkB+ Lanceolate endings, (E) 197 TrkB+ Lanceolate endings: *Cntnap2*$^{+/+}$ n=3 animals, (D) 40 TrkB+ Lanceolate endings, (E) 220 TrkB+ Lanceolate endings). RP=running pad.

For B and C students t-test*p<0.05, **p<0.01 versus *Cntnap2*$^{+/+}$. All data shown as mean ±SEM.

Supplementary Figure 10 No change in spontaneous or capsaicin evoked EPSCs in dorsal horn neurons from *Cntnap2*$^{-/-}$ mice. Related to Figure 7

(A) Representative voltage-clamp traces showing spontaneous and capsaicin (1μM) evoked EPSCs in lamina II dorsal horn neurons from *Cntnap2*$^{-/-}$ (top) and control mice (bottom).
(B) Quantification of the frequency of EPSCs recorded for 3 minutes at baseline and during capsaicin application. A clear increase in EPSC frequency is seen in both *Cntnap2*$^{-/-}$ and *Cntnap2*$^{+/+}$ mice following capsaicin treatment. However, there was no difference in the frequency of capsaicin-evoked EPSCs between genotypes.
(C) Quantification of the amplitude of EPSCs recorded at baseline and during capsaicin application. There was no difference in the amplitude of spontaneous or capsaicin-evoked EPSCs between genotypes.
Data was taken from 6 *Cntnap2*$^{-/-}$ (n=15 cells) and 7 *Cntnap2*$^{+/+}$ mice (n=16 cells). All data shown as the mean±SEM.

Table S1 qPCR analysis of gene expression in sciatic nerve and DRG from control versus Patient IgG treated mice. Related to Figure 2

Gene name	Sciatic nerve		DRG	
	**Control IgG**	**Patient IgG**	**Control IgG**	**Patient IgG**
Ccl2^	1.0±0.25	0.54±0.08	1.0±0.14	0.99±0.06
Ifnb1	1.0±0.19	0.60±0.20	ND	ND
Il18	1.0±0.37	0.92±0.35	1.0±0.21	0.97±0.24
Il1B	ND	ND	ND	ND
Il6	1.0±0.73	0.87±0.52	1.0±0.53	1.56±0.58
Tnf	ND	ND	ND	ND

Samples were pooled from patient 1 and 2 IgG treated mice and controls to reduce variability. No significant differences were seen in the expression of cytokines and chemokines between treatment groups, n=6 for sciatic nerve control IgG, n=7 for all other groups. ND indicates that the transcript was not detected. Expression was normalised to control values and the fold change is shown for the Patient IgG group. ^qPCR performed using SYBR green. All other qPCR performed using Taqman probes. All data shown as the mean±SEM.

Table S2. EM analysis of sural nerve in mice treated with either healthy control or patient IgG. Related to Figure 2

Parameter	Control IgG	Patient 1 IgG	Control IgG	Patient 2 IgG
No. of myelinated fibres x1000/mm^2	38.9±4.4	40.6±3.2	33.9±6.3	39.0±11.1
No. of unmyelinated fibres x1000/mm^2	152.4±44.4	139±14.7	172.7±27.4	104.4±16.6
No. of remak bundles x1000/mm^2	14.5±3.9	13.6±1.4	12.1±2.0	8.1±0.05
Average no. of C-fibres/remak bundle	10.3±0.5	10.2±0.3	13.6±0.5	12.3±2.0
Schwann cell nuclei x1000/mm^2	1.1±0.03	0.9±0.45	1.7±0.2	1.6±0.1

3 animals used per group. No significant difference between treatment groups. All data shown as mean±SEM.

Table S3 Locomotor activity in *Cntnap2*$^{-/-}$ mice. Related to Figure 3

Behavioural test	*Cntnap2*$^{+/+}$	No. of mice	*Cntnap2*$^{-/-}$	No. of mice
Beam test – Percentage of correct steps	87.8±2.6%	6	83.4±3.6%	4
Accelerating RotaRod (4-32rpm)	179.2±12.3	13	216.9±27.4	8
Constant speed RotaRod (28rpm)	53.2±12.1	13	138.2±39.6*	8
Open field – No. of grid boxes entered in 3 minute period	131.7±14.3	13	148.7±17.4	8

Student's t test *p<0.05 versus *Cntnap2*$^{+/+}$ group. All data shown as mean±SEM.

Table S4 Expression levels of pain-related genes are not changed in the DRG of *Cntnap2*$^{-/-}$ mice. Related to Figure 3

Gene	*Cntnap2*$^{+/+}$	*Cntnap2*$^{-/-}$
Atf3^	1.0±0.08	1.0±0.1
Bdnf	1.0±0.05	0.83±0.07
Cana2d1	1.0±0.04	1.0±0.06
Cntn2^	1.0±0.07	1.2±0.05
Hcn2	1.0±0.11	1.1±0.07
Kcna1^	1.0±0.13	1.4±0.15
Kcna2^	1.0±0.07	1.2±0.17
Kcns1	1.0±0.06	0.96±0.02
Ngf	1.0±0.09	1.0±0.08
Ngfr	1.0±0.1	0.97±0.06
Ntrk1	1.0±0.04	0.98±0.07
Ntrk2	1.0±0.05	0.99±0.06
Ntrk3	1.0±0.03	1.0±0.03
Oprm1	1.0±0.03	0.91±0.03
Pdyn	1.0±0.1	1.2±0.08
Ptgs2	1.0±0.4	0.52±0.09
P2rx3	1.0±0.1	1.0±0.09
Scn3a	1.0±0.3	0.86±0.12
Scn9a	1.0±0.08	0.92±0.03
Scn10a	1.0±0.04	0.95±0.02
Scn11a	1.0±0.07	0.97±0.05
Trpa1	1.0±0.03	0.96±0.08
Trpm8^	1.0±0.1	0.96±0.08
Trpv1	1.0±0.08	0.89±0.07
VGF	1.0±0.1	1.0±0.4

Relative gene expression from mouse Lumbar DRG, n=4 per genotype. No statistically significant differences found between genotypes, students t test. ^qPCR performed using SYBR green. All other qPCR performed using Taqman probes. All data shown as mean±SEM.

Table S5 Biophysical properties in cultured DRG neurons from *Cntnap2*$^{-/-}$ are normal. Related to Figure 5

	Small (<25µm)		Medium (25-35 µm)		Large (>35 µm)	
	+/+	*-/-*	*+/+*	*-/-*	*+/+*	*-/-*
**R_{Input} (MΩ)**	327.11±11.43	335.42±16.01	98.33±10.32	96.76±10.34	57.89±17.90	53.22±13.87
**RMP (mV)**	-49.74±1.73	-48.97±1.81	-61.87±0.85	-59.19±1.45	-61.93±2.16	-63.83±0.70
**Capacitance (pF)**	23.39±0.93	21.87±1.35	31.75±1.18	31.10±2.22	63.50±4.59	70.23±5.25
**No. of cells**	47	49	20	20	12	13

No statistical difference found between genotypes. All data shown as mean±SEM

Table S6. Stimulus response and mechanical latency in primary afferent fibres in *Cntnap2*$^{+/+}$ and *Cntnap2*$^{-/-}$ mice. Related to Figure 6

		RAM		SAM		D-Hair		A-M		C-M		C-MH	
Cntnap2		+/+	-/-	+/+	-/-	+/+	-/-	+/+	-/-	+/+	-/-	+/+	-/-
n		23	19	14	22	14	20	25	20	20	19	15	13
Conduction velocity (m/s)		13.91 ±0.54	13.88 ±0.59	14.02 ±0.75	14.19 ±0.55	5.66 ±0.73	7.07 ±0.41	5.15 ±0.47	5.49 ±0.44	0.86 ±0.05	0.73 ±0.04	0.51 ±0.02	0.43 ±0.11
Mechanical threshold (mN)		10.08 ±2.79	10.40 ±2.26	12.22 ±1.72	9.76 ±2.27	1.31 ±0.40	0.67 ±0.20	49.91 ±2.70	49.20 ±2.93	66.49 ±8.97	70.72 ±12.49	114.0 ±27.58	109.60 ±21.97
Stimulus-Response	Velocity µm/s	Firing frequency (Hz)											
	75	5.45 ±1.94	3.03 ±0.80	14.50 ±3.41	14.50 ±2.48	6.26 ±1.04	12.91 ±1.77						
	150	10.65 ±2.65	8.04 ±1.76	16.59 ±4.40	19.28 ±3.68	17.28 ±1.78	26.45 ±2.73						
	450	27.93 ±4.45	20.09 ±3.33	29.85 ±6.54	34.59 ±5.40	50.33 ±5.40	59.49 ±4.56						
	1,500	74.61 ±9.39	59.17 ±8.64	67.09 ±11.27	69.47 ±9.23	125.76 ±13.41	132.41 ±8.83						
	15,000	264.24 ±23.20	261.92 ±20.76	209.74 ±18.56	192.34 ±16.93	270.86 ±34.35	218.89 ±25.75						
Stimulus-Response	Force mN	Firing frequency (Hz)											
	14			3.33 ±1.32	3.92 ±1.38			2.24 ±0.79	1.67 ±0.79	4.45 ±0.93	4.10 ±0.70	1.61 ±0.60	2.62 ±0.75
	49			9.73 ±3.50	11.18 ±3.69			7.16 ±2.083	5.54 ±2.18	10.05 ±1.83	9.45 ±1.47	4.43 ±1.54	5.70 ±1.36
	96			13.42 ±3.69	12.74 ±2.55			12.15 ±2.20	9.45 ±2.03	13.81 ±2.29	15.44 ±2.56	7.14 ±2.07	7.14 ±1.43
	190			20.17 ±4.52	13.70 ±2.29			21.20 ±2.40	19.72 ±2.78	20.57 ±2.60	26.31 ±3.41	9.96 ±2.10	9.93 ±1.93
	292			13.56 ±3.53	10.98 ±1.77			26.00 ±2.75	23.04 ±2.03	22.36 ±3.44	25.15 ±3.02	11.35 ±2.61	9.38 ±2.10
		µm/s											
Mechanical latency	Stim 1	475.70 ±95.56	461.70 ±90.56	413.77 ±43.84	332.63 ±39.91	161.30 ±42.13	95.87 ±37.54	92.61 ±74.25	76.56 ±62.61	115.48 ±62.88	116.24 ±57.16	186.08 ±61.92	357.15 ±154.36
	Stim 2	224.82 ±39.46	205.38 ±40.18	235.06 ±23.69	223.85 ±29.78	73.70 ±19.14	52.00 ±15.61	76.40 ±58.14	18.74 ±3.45	40.08 ±12.00	60.58 ±36.56	115.31 ±77.30	69.52 ±18.02
	Stim 3	60.10 ±10.71	65.80 ±9.65	75.83 ±6.33	63.84 ±8.98	13.93 ±3.22	15.38 ±5.01	30.43 ±9.25	22.56 ±4.60	21.99 ±3.03	22.10 ±2.20	131.40 ±64.65	38.18 ±6.61
	Stim 4	18.00 ±2.92	18.35 ±2.31	22.85 ±1.98	23.53 ±2.72	5.39 ±1.10	6.74 ±1.32	27.73 ±3.60	25.27 ±4.75	26.27 ±5.03	24.63 ±2.20	80.66 ±36.34	39.05 ±6.16
	Stim 5	3.22 ±0.23	3.35 ±0.21	3.76 ±0.24	4.71 ±0.50	2.08 ±0.23	2.75 ±0.27	28.92 ±3.35	28.00 ±4.85	32.26 ±4.31	37.28 ±4.64	60.17 ±11.19	42.54 ±6.50

Data taken from 16 *Cntnap2*$^{+/+}$ and 15 *Cntnap2*$^{-/-}$ mice. Note: adaptation properties were unchanged between genotypes for AMs and SAMs during the Hold phase of the Ramp hold protocol at 190mN and 292mN. All data shown as mean±SEM.

Table S7. Primary and Secondary antibodies and fluorescent dyes used for histology. Related to STAR methods.

Antigen	Company	Host/Fluorophore	Dilution
ATF3	Santa Cruz	Rabbit	1:500
CGRP	Enzo Life Sciences	Sheep	1:400
CGRP	Peninsula Laboratories	Rabbit	1:1000
IB4 target	Sigma	Conjugated to biotin	1:100
PGP9.5	Ultraclone	Rabbit	1:1000
NF200	Sigma	Mouse	1:250
NF200	Abcam	Chicken	1:10000
CASPR2	Abcam	Rabbit	1:100
CASPR	Gift – Bhat MA	Guinea pig	1:1500
IBA1	Wako	Rabbit	1:1000
Kv1.1	Gift – Trimmer J	Rabbit	1:200
Kv1.2	UC Davis/NIH NeuroMab Facility	Mouse	1:100
Beta-III-tubulin	Sigma	Mouse	1:1000
Pax2	Thermo Fisher Scientific	Rabbit	1:200
NeuN	Abcam	Rabbit	1:1000
NeuN	Millipore	Chicken	1:500
TrkB	R&D Systems	Goat	1:250
Tyrsosine hydroxylase	Millipore	Sheep	1:200
Gephyrin	Frontier Institute	Rabbit	1:200
Vgat	Frontier Institute	Goat	1:200
c-fos	Santa Cruz	Rabbit	1:500
CD3	Abcam	Rabbit	1:500
CD68	BioRad	Rat	1:400
Ly6G	R&D Systems	Rat	1:100
GFAP	Dako	Rabbit	1:500
GFP	Abcam	Chicken	1:1000
Rabbit IgG	Thermo Fisher Scientific	Alexa 488	1:1000
Rabbit IgG	Jackson ImmunoResearch	Cy3	1:500

	Labs		
Rabbit IgG	Thermo Fisher Scientific	Pacific Blue	1:500
Rabbit IgG	Thermo Fisher Scientific	546	1:500
Sheep IgG	Jackson ImmunoResearch Labs	Cy3	1:500
Streptavidin	Thermo Fisher Scientific	Alexa 405	1:500 and 1:100
Streptavidin	Thermo Fisher Scientific	Pacific blue	1:500
Mouse IgG	Thermo Fisher Scientific	Alexa 488	1:1000
Mouse IgG	Thermo Fisher Scientific	Pacific Blue	1:500
Guinea pig IgG	Thermo Fisher Scientific	Alexa 568	1:500
Human IgG	Thermo Fisher Scientific	Alex 488	1:1000
Human IgG	Thermo Fisher Scientific	Alex 546	1:1000
Nissl	Thermo Fisher Scientific	NeuroTrace 530/615	1:100
Sheep IgG	Thermo Fisher Scientific	Alexa 488	1:1000
Rat IgG	Thermo Fisher Scientific	Alexa 488	1:500
Rat IgG	Thermo Fisher Scientific	Alexa 546	1:500
Chicken IgG	Thermo Fisher Scientific	Alexa 488	1:1000
Chicken IgG	Thermo Fisher Scientific	Alexa 546	1:1000

Table S8. Sequences or assay IDs for primers used in qPCR. Related to STAR methods.

Gene name	Forward primer (5'-3')	Reverse primer (5'-3')	Assay ID
Trpm8	CAGCCTGGGGTCCAGAATTT	AATGATACGAGGCCACAGCC	-
Aft3	GTGCCTGCAGAAAGAGTCAGA	GAGGTTCCTCTCGTCTTCCG	-
FL-Cntnap2	GCTCCCTCACCACTGACTTC	GCAAGTTTCCTGTGGGCTTG	-
SH-Cntnap2	ATTCCAGCATCTGGCGACAA	CCTTGGGATGCAGGTGGATT	-
Kcna1	TTACCCTGGGCACGGAGATA	ACACCCTTACCAAGCGGATG	-
Kcna2	CATCTGCAAGGGCAACGTCAC	CCTTTGGAAGGAAGGAGGCA	-
Cntn2	CCCAGTATCAAAGCCCGGTT	ACAGGGTTCCCAAAGGCAAA	-
Ccl2	CTGTAGTTTTTGTCACCAAGCTCA	GTGCTGAAGACCTTAGGGCA	
18s	GGACCAGAGCGAAAGCATTTG	GCCAGTCGGCATCGTTTATG	-
Hprt1	GTCCTGTGGCCATCTGCCTAG	TGGGGACGCAGCAACTGACA	-
Gapdh	TGTGTCCGTCGTGGATCTGA	TTGCTGTTGAAGTCGCAGGAG	-
Hcn2	-	-	Mm00468538_m1
Kcns1	-	-	Mm00492824_m1
P2rx3	-	-	Mm00523699_m1
Scn3a	-	-	Mm00658167_m1
Scn9a	-	-	Mm00450762_s1
Scn10a	-	-	Mm00501467_m1
Scn11a	-	-	Mm00449377_m1
Trpa1	-	-	Mm01227437_m1
Trpv1	-	-	Mm01246302_m1
Cacna2d1	-	-	Mm00486607_m1
Bdnf	-	-	Mm04230607_s1
Ngf	-	-	Mm00443039_m1
Ngfr	-		Mm01309635_m1
Ntrk1	-	-	Mm01219406_m1
Ntrk2	-	-	Mm00435422_m1
Ntrk3	-	-	Mm00456222_m1
Vgf	-	-	Mm01204485_s1
Oprm1	-	-	Mm00440568_m1
Pdyn	-	-	Mm00457573_m1
Ptgs2	-	-	Mm00478374_m1
Ifnb1			Mm00439552_s1
Il18			Mm00434225_m1
Il1B			Mm01336189_m1
Il6			Mm99999064_m1
Tnf			Mm99999068_m1
18s	-	-	Hs99999901_s1
Hprt1	-	-	Mm00446968_m1
Gapdh	-	-	Mm99999915_g1

Data S1 Patient 2 case history. Related to Figure 1&2

A 71 year old man presented with a 2 year history of burning pain in the feet; when walking. He described the sensation as if walking on glass. He subsequently developed paraesthesia of the feet as well as paraestheia and clumsiness of the hands. He denied muscle weakness but had noted that his balance had deteriorated. He had developed a dry mouth and impotence but denied any other autonomic symptoms. He had noted that his speech had become slurred and at times noted difficulty swallowing. He complained of cognitive symptoms and in particular short term memory loss. He also developed new episodes of brief alterations in consciousness which lasted 10 seconds, were associated with a sensation he described as 'goosebumps' and reduced awareness of his surroundings. These occurred up to 10 times a day and were thought to potentially represent complex partial seizures. He had a past medical history of type II diabetes mellitus diagnosed 5 years ago which was well controlled on metformin 500mg TDS with no diabetic retinopathy on retinal screening and no micro-albuminuria. His pain had not responded to amitriptyline but he reported some improvement on Gabapentin 600mg TDS. On pain screening questionnaires he scored 4 on the DN4 and 25 on PainDETECT consistent with a diagnosis of neuropathic pain. The pain severity score on the Brief Pain Inventory score was 6.83 and pain interference score was 4.25. On a body map he labelled the pain as affecting his hands and feet.

On clinical examination blood pressure was 120 mmHg systolic and 62 mmHg diastolic with no postural drop. Eye movements were normal. He had a mild dysarthria and broad based gait with a positive Romberg's sign. There was no muscle wasting, power was normal throughout. All deep tendon reflexes were preserved other than the ankle jerks which were absent. On sensory examination pin prick, light touch and proprioception were normal. Vibration sense was impaired in the toes. Thermal thresholds (cool detection, warm detection and thermal sensory limen) were normal.

Nerve conduction studies showed normal sensory and motor conduction and on EMG there was no evidence of neuromyotonia. A skin biopsy was taken from 10cm above the left lateral malleolus and intra-epidermal nerve fibre density was normal relative to age and gender matched controls (4.7 fibres/mm). MRI of the brain and spinal cord was normal with no evidence of cerebellar atrophy and no features suggestive of limbic encephalitis. A barium swallow demonstrated laryngeal penetration of liquids and occasionally solids.

A Dendrotoxin assay for anti-voltage gated potassium channel complex antibodies was strongly positive (2689 pmol/l), with the presence of antibodies directed against CASPR2 but not LGI1. The patient was treated with prednisolone and 5 plasma exchanges. The titre of anti-voltage gated potassium channel complex antibody dropped to 602pmol/l (CASPR2 antibodies remained positive). The episodes of altered consciousness resolved and his balance improved. There was also some improvement in the burning pain and paraesthesia although these symptoms did not completely resolve. He was managed with ongoing immunosuppression with mycophenolate 1g BD.
