# Supplementary material for: Immune or Genetic-Mediated Disruption of CASPR2 Causes Pain Hypersensitivity Due to Enhanced Primary Afferent Excitability
Source: Neuron. 2018 Feb 21;97(4):806–822.e10. doi: 10.1016/j.neuron.2018.01.033 (PMC6011627; doi:10.1016/j.neuron.2018.01.033)
Supplement: Document S2. Article plus Supplemental Information [file mmc2.pdf]

Article

Neuron

Immune or Genetic-Mediated Disruption of CASPR2 Causes Pain Hypersensitivity Due to Enhanced Primary Afferent Excitability

Highlights

- Treatment with human CASPR2-Abs causes mechanical hypersensitivity in mice
- CASPR2-Abs remain PNS restricted and enhance pain behavior without neural injury
- Pain behavior is increased in *Cntnap2*$^{-/-}$ mice (lacking full-length CASPR2)
- CASPR2 regulates DRG cell excitability via Kv1 channel trafficking to the membrane

Authors

John M. Dawes, Greg A. Weir, Steven J. Middleton, ..., Gary R. Lewin, Angela Vincent, David L. Bennett

Correspondence

david.bennett@ndcn.ox.ac.uk

In Brief

Dawes et al. show that CASPR2, the target of an autoimmune pain disorder, regulates sensory function through tuning of primary afferent excitability. Human autoantibodies to CASPR2 cause pain-related hypersensitivity and enhance DRG cell excitability through reduced Kv1 channel function.

Dawes et al., 2018, Neuron *97*, 806–822
February 21, 2018 © 2018 The Author(s). Published by Elsevier Inc.
https://doi.org/10.1016/j.neuron.2018.01.033

Neuron
Article

Immune or Genetic-Mediated Disruption of CASPR2 Causes Pain Hypersensitivity Due to Enhanced Primary Afferent Excitability

John M. Dawes,[1] Greg A. Weir,[1] Steven J. Middleton,[1] Ryan Patel,[2] Kim I. Chisholm,[3] Philippa Pettingill,[1] Liam J. Peck,[1] Joseph Sheridan,[1] Akila Shakir,[1] Leslie Jacobson,[1] Maria Gutierrez-Mecinas,[4] Jorge Galino,[1] Jan Walcher,[5] Johannes Kühnemund,[5] Hannah Kuehn,[1] Maria D. Sanna,[1] Bethan Lang,[1] Alex J. Clark,[1] Andreas C. Themistocleous,[1] Noboru Iwagaki,[4] Steven J. West,[1] Karolina Werynska,[1] Liam Carroll,[1] Teodora Trendafilova,[1] David A. Menassa,[1] Maria Pia Giannoccaro,[1] Ester Coutinho,[1] Ilaria Cervellini,[1] Damini Tewari,[3] Camilla Buckley,[1] M. Isabel Leite,[1] Hendrik Wildner,[6] Hanns Ulrich Zeilhofer,[6,7] Elior Peles,[8] Andrew J. Todd,[4] Stephen B. McMahon,[3] Anthony H. Dickenson,[2] Gary R. Lewin,[5] Angela Vincent,[1] and David L. Bennett[1,9,*]
[1]Nuffield Department of Clinical Neurosciences, University of Oxford, Oxford OX3 9DU, UK
[2]Department of Neuroscience, Physiology and Pharmacology, University College London, London WC1E 6BT, UK
[3]Neurorestoration Group, Wolfson Centre for Age-Related Diseases, King's College London, London SE1 1UL, UK
[4]Institute of Neuroscience and Psychology, College of Medical, Veterinary and Life Sciences, University of Glasgow, Glasgow G12 8QQ, UK
[5]Molecular Physiology of Somatic Sensation, Max Delbrück Center for Molecular Medicine, Berlin, Germany
[6]Institute of Pharmacology and Toxicology, University of Zurich, Winterthurerstrasse 190, 8057 Zürich, Switzerland
[7]Institute of Pharmaceutical Sciences, Swiss Federal Institute of Technology (ETH) Zurich, Vladimir-Prelog-Weg 10, 8093 Zurich, Switzerland
[8]Department of Molecular Cell Biology, Weizmann Institute of Science, Rehovot 76100, Israel
[9]Lead Contact
*Correspondence: david.bennett@ndcn.ox.ac.uk
https://doi.org/10.1016/j.neuron.2018.01.033

SUMMARY

Human autoantibodies to contactin-associated protein-like 2 (CASPR2) are often associated with neuropathic pain, and CASPR2 mutations have been linked to autism spectrum disorders, in which sensory dysfunction is increasingly recognized. Human CASPR2 autoantibodies, when injected into mice, were peripherally restricted and resulted in mechanical pain-related hypersensitivity in the absence of neural injury. We therefore investigated the mechanism by which CASPR2 modulates nociceptive function. Mice lacking CASPR2 (*Cntnap2*$^{-/-}$) demonstrated enhanced pain-related hypersensitivity to noxious mechanical stimuli, heat, and algogens. Both primary afferent excitability and subsequent nociceptive transmission within the dorsal horn were increased in *Cntnap2*$^{-/-}$ mice. Either immune or genetic-mediated ablation of CASPR2 enhanced the excitability of DRG neurons in a cell-autonomous fashion through regulation of Kv1 channel expression at the soma membrane. This is the first example of passive transfer of an autoimmune peripheral neuropathic pain disorder and demonstrates that CASPR2 has a key role in regulating cell-intrinsic dorsal root ganglion (DRG) neuron excitability.

INTRODUCTION

Autoantibodies against contactin-associated protein-like 2 (CASPR2-Abs) have been linked to a number of clinical syndromes. These include neuromyotonia, in which there is clinical and electrophysiological evidence of excessive motor unit activity due to enhanced motor axon excitability; Morvan's syndrome, in which neuromyotonia is associated with autonomic and CNS dysfunction (particularly insomnia); and limbic encephalitis, which is characterized by cognitive impairment and epilepsy (Irani and Vincent, 2016). A common feature described in patients seropositive for CASPR2-Abs is the presence of neuropathic pain, and in some patients this was the sole presenting symptom (Irani et al., 2012; Klein et al., 2012; Lancaster et al., 2011). Furthermore, immunosuppression to reduce levels of CASPR2-Ab can lead to a reduction in neuropathic pain (Klein et al., 2012). Whether CASPR2 has a direct role in nociceptive signaling and the mechanisms by which such CASPR2-Abs could drive neuropathic pain are unknown.

CASPR2 is a neuronal adhesion molecule of the neurexin superfamily that is known to form a protein complex with shaker-type voltage-gated potassium channels (such as Kv1.1 and Kv1.2) (Horresh et al., 2008). CASPR2 is, therefore, one of a group of proteins that form the voltage-gated potassium channel complex (VGKCC) that also includes LGI1 and contactin-2 (Irani et al., 2010). Antibodies to the VGKCC are not directed against Kv1 channels themselves but to proteins with which they form a complex (Irani et al., 2010). Of the components of this complex, it is particularly antibodies to CASPR2 that have been associated with neuropathic pain (Irani et al., 2012; Klein et al., 2012).

 © 2018 The Author(s). Published by Elsevier Inc.
This is an open access article under the CC BY license (http://creativecommons.org/licenses/by/4.0/).

The extracellular domain of CASPR2 binds to Contactin-2 and is required for the correct longitudinal localization of Kv1 channels to the juxtaparanode (JXP) of myelinated axons (Poliak et al., 2003). In mice lacking CASPR2 or Contactin-2, Kv1 channels were no longer clustered at the JXP. The functional implications of this mislocalization have hitherto been unclear because, in the naive state, Kv1 channels at the JXP are electrically isolated from the node of Ranvier and paranode by compact myelin, and axonal excitability was reported to be unaltered in these mice (Poliak et al., 2003). Following injury to myelin or the distal axon, however, Kv1 channels (and CASPR2) are redistributed to the paranode and can suppress axonal hyperexcitability (Calvo et al., 2016; Rasband et al., 1998). Kv1.1 and Kv1.2 also have important roles in modulating somatic excitability of DRG neurons (Gold et al., 1996; Hao et al., 2013; Zhao et al., 2013). However, although CASPR2 is known to regulate longitudinal clustering of Kv1 channels along myelinated fibers, its function in trafficking Kv1 channels to the dorsal root ganglion (DRG) soma membrane and the effects on excitability are unknown.

CASPR2 has also been identified as a synaptic protein with a role in synapse development/maintenance and has recently been linked to neurodevelopmental processes (Rodenas-Cuadrado et al., 2014): humans with homozygous loss-of-function mutations in *CNTNAP2* (the gene encoding CASPR2) develop epilepsy and developmental delay with a number of core features of autistic spectrum disorder (ASD) (Strauss et al., 2006). Mice lacking CASPR2 have been shown to develop autistic traits at a behavioral level, associated with deficits in the migration of cortical inhibitory interneurons (Peñagarikano et al., 2011). Somatosensory abnormalities have recently been recognized as a common feature of ASDs (Cascio, 2010) and are now part of diagnostic criteria. Given the reported association of CASPR2-Abs with pain as well as the increasing recognition of somatosensory abnormalities as a core feature of ASDs, we undertook a detailed characterization of the role of CASPR2 in the regulation of sensory function.

RESULTS

Patient CASPR2-Abs Cause Pain-Related Hypersensitivity in Mice

To investigate the potential pathogenicity of CASPR2-Abs, we injected mice with patient-derived purified IgG (obtained from two CASPR2-Ab-positive patients with neuropathic pain who had received plasma exchange treatment) and assessed pain-related behavior. Patient 1 had Morvan's syndrome with typical features of neuromyotonia, dysautonomia, pain, and severe insomnia. He improved considerably with plasma exchange (that reduces circulating antibody levels by >80%) (Liguori et al., 2001). Patient 2 presented with cerebellar ataxia and neuropathic pain that was particularly localized to the feet; there was, however, no clinical or electrophysiological evidence of neuromyotonia (patient 2 information shown in Data S1). The antibodies in patient 1 were originally identified by radioimmunoprecipitation of VGKC complexes from rabbit brain tissue, but were then shown to be directed against CASPR2 using a live cell-based assay (CBA) (Irani et al., 2010). Both patients 1 and 2 had very high titers of CASPR2 IgG in their sera, plasmas, and purified IgG preparations (1:62,500 or higher; Figure 1A). Antibodies to LGI1, the other main VGKC complex protein, were only just detectable (1:20) in patient 1 IgG and negative in patient 2 IgG.

We gave mice systemic injections of purified patient IgG or IgG from a healthy control for either 14 or 22 days (dosing and behavioral testing regime is shown in Figure 1B). At the end of the experiment, the CASPR2-IgG-treated mice had very high CASPR2 titers (maximal binding at 1:100, titrating out to 1:12,500 or higher). No LGI1 antibodies were detected in the mice. Over the course of the experiment, there was no significant weight loss compared to baseline or between groups (Figures S1A and S1D). Mice treated with purified IgG from patient 1 developed a significant delayed-onset mechanical hypersensitivity when compared to control IgG-treated mice, beginning after 11 days of injections (withdrawal threshold to von Frey hair of 0.58 ± 0.04 g control IgG versus 0.41 ± 0.09 g patient 1 IgG) (Figure 1C), with a greater effect seen after 14 days (0.6 ± 0.05 g control IgG versus 0.31 ± 0.05 g patient 1 IgG) (Figure 1C). Mice treated with purified IgG from patient 2 also developed a delayed-onset mechanical hypersensitivity, which was significantly different from the control IgG group 15 days after the initial injection (0.6 ± 0.1g control IgG versus 0.32 ± 0.07 g patient 2 IgG) (Figure 1E). Although a significant reduction in thermal withdrawal thresholds was seen for mice treated with patient 2 IgG at day 15 (Figure 1F), in general thermal thresholds were similar to those of control mice (Figures 1D and 1F). We found no difference between treatment groups in spontaneous locomotor activity or rearing behavior in the open field test (Figures S1B and S1E, and S1C and S1F, respectively). We did not observe any spontaneous nocifensive behavior such as licking, biting, or paw-lifting.

Patient CASPR2-Abs Bind *In Vivo* but Do Not Cause Overt Inflammation or Substantial Damage to the Nervous System

Using anti-human IgG antibodies to detect bound IgG, we assessed CASPR2-Ab deposition in tissue taken from the mice. No immunoreactivity for human IgG was found in the spinal cord (Figure 2A), suggesting that patient IgG did not cross the blood-cord barrier. We did see some human IgG deposited in the sciatic nerve (Figure S2A), but did not see any specific binding to the JXP. A previous study using patient CASPR2-Abs also did not see binding to axons in intact nerve, suggesting that Abs are unable to cross the tight junction at the paranode to access their target within the JXP (Manso et al., 2016). This is in stark contrast to permeabilized frozen nerve sections, where clear binding in the JXP can be seen (Figure S2B). At the level of the DRG, however, CASPR2 is more easily accessible on the neuronal soma and clear binding of human IgG can be seen on the surface of sensory neurons (Figure 2B); this suggests that the ability of CASPR2 to increase pain-related behavior in mice could be due to its action in the peripheral nervous system, particularly at the level of the DRG.

CASPR2-Ab could cause neuropathic pain as a consequence of neuro-inflammation. To address this, we assessed cellular infiltration of neutrophils, macrophages, and lymphocytes, using the markers Ly6G, IBA1, and CD3, respectively, in the DRG. There was no difference in any of these markers between control

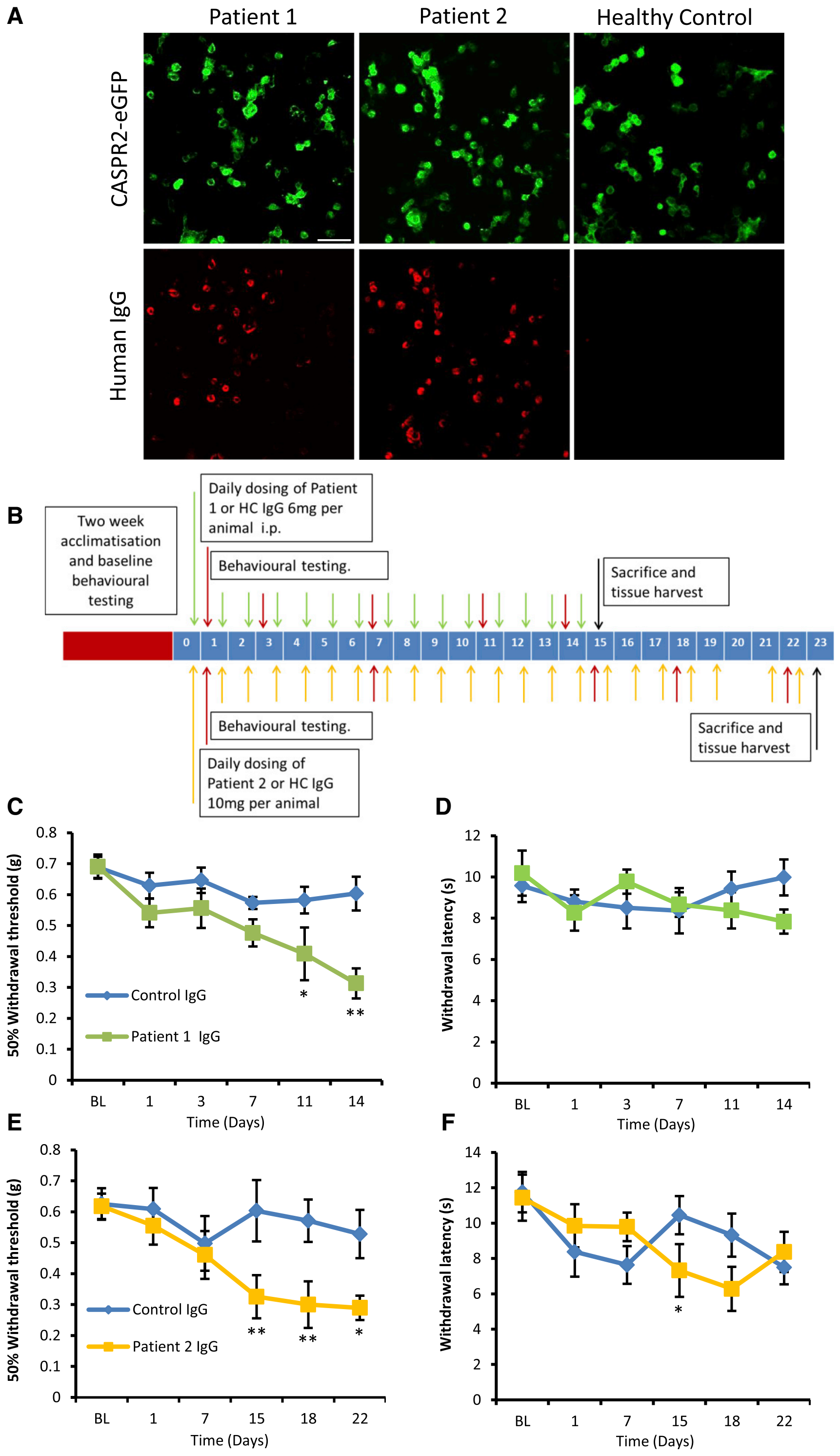

Figure 1. Passive Transfer of Human CASPR2-Abs Causes Pain-Related Hypersensitivity in Mice

(A) CBA showing binding of antibodies from patient plasma using an anti-human IgG secondary antibody (red) to HEK cells transfected with human CASPR2-EGFP. No binding is seen using plasma from a healthy control subject. Scale bar, 50 μm.

(B) Dosing regime and behavioral time course for passive transfer of WT mice with purified IgG from CASPR2-Ab-positive patients.

(C–F) Using von Frey hairs, mice treated with patient 1 and 2 IgG develop a significant mechanical pain-related hypersensitivity (C and E, respectively) when compared to mice treated with IgG from a healthy control subject. Mice did not, however, develop a clear thermal hypersensitivity using the Hargreaves testing method (D and F). For (C) and (D), n = 8, and for (E) and (F), n = 9.

Data shown as mean ± SEM, $^*p < 0.05$, $^{**}p < 0.01$ versus control IgG group. See also Figure S1.

saw no significant differences between animals treated with patient IgG versus control (Table S1).

Similarly, we did not observe a significant inflammatory response within the spinal cord. There were no Ly6G-positive cells in the spinal cord (Figures S3A and S3B). Counts for CD3-positive cells were low and no difference was found between treatment groups (Figures S3C and S3D). We also saw no evidence of astrocytosis as measured using GFAP (Figures S3E and S3F). Although we observed a small but significant increase in the density of IBA1-positive microglia in the spinal cord in mice treated with patient 1 IgG (Figure S3G), we did not see any differences for patient 2-treated mice (Figure S3H). Recent work has shown that exposure of mice *in utero* to CASPR2-Abs can cause microglia activation in specific brain regions (Coutinho et al., 2017). Therefore, we assessed microglia in the primary somatosensory cortex, but saw no difference in microglia in terms of both cell density and morphology (Figures S3I–S3O). These data, coupled with lack of weight loss (Figures S1A and S1D), suggest that patient autoantibodies did not cause a gross inflammatory response.

To investigate possible Ab-mediated neural injury, we measured intra-epidermal nerve fiber density (IENFD) in the paw that did not differ between animals treated with IgG from either healthy control or CASPR2-Ab-positive patients (Figures 2G and S2G). This is in line with patient data describing normal IENFD density (Data S1). Activating transcription factor 3 (ATF3) is upregulated by sensory neurons

and patient IgG-treated mice (Figures 2D–2F and S2D–S2F). Additionally, there was no difference in IBA1-positive cells in the sciatic nerve between treatment groups (Figures 2C and S2C). We also measured the expression of a number of cytokines and chemokines in the DRG and nerve using qPCR and

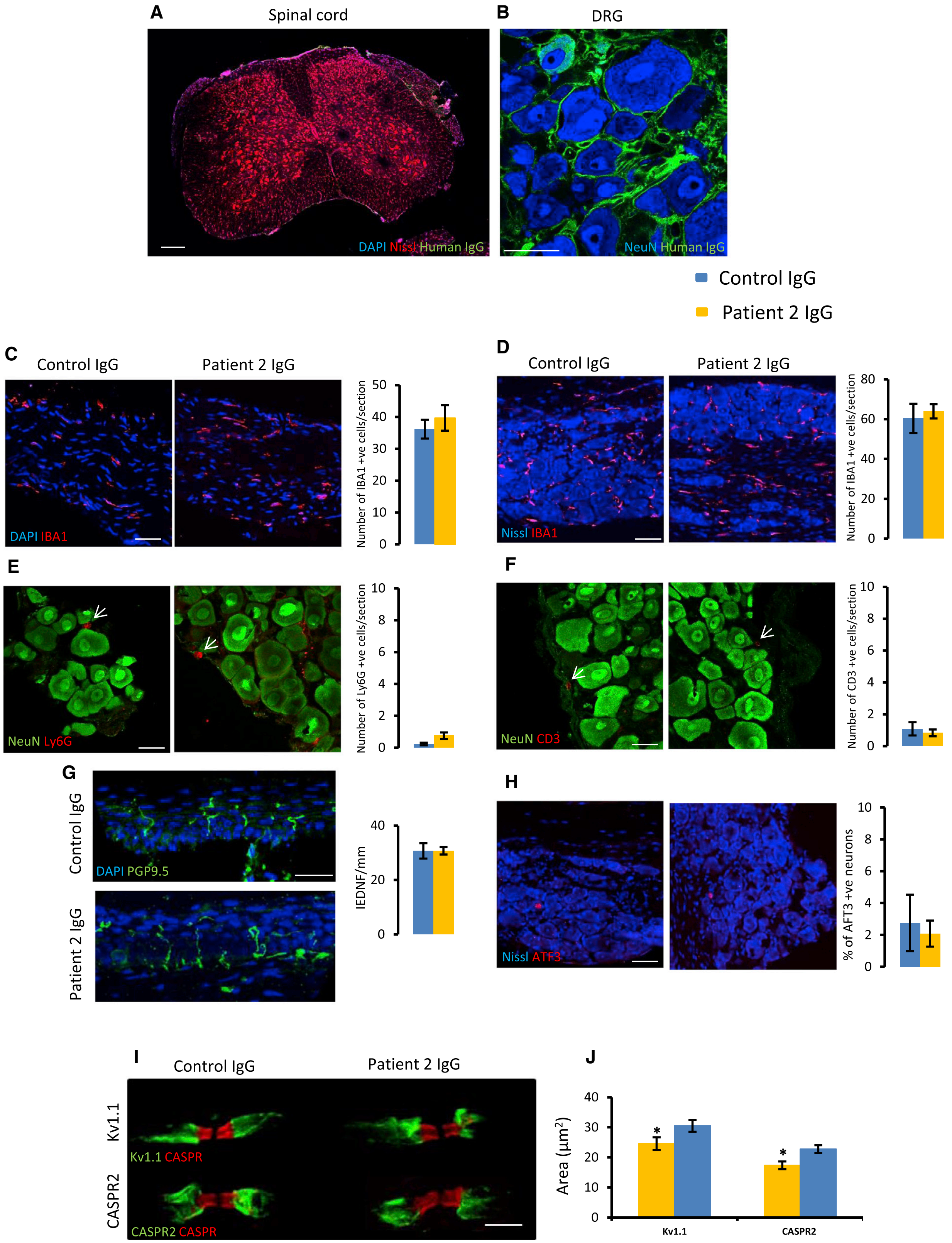

(legend on next page)

following injury (Tsujino et al., 2000). No difference was found in DRG neuronal ATF3 expression from control and patient 2 IgG-treated mice (Figure 2H); a small but significant increase in ATF3 immunoreactive DRG profiles was found in mice treated with IgG from patient 1 (Figure S2H) and may explain the small increase in microglial density observed in Figure S3G. We also assessed the structure of peripheral nerve in more detail using electron microscopy (EM). We saw no evidence of autoantibody-mediated demyelination. G ratios were the same between the control and patient IgG-treated groups (Figures S4A–S4C and S4E), and there were no significant differences in axon diameter or the total number of axons (Figures S4D, S4F, and S4G; Table S2). We also studied the organization of nodal sub-domains. No differences were seen in the total number of nodes (Figures S4H and S4K), or in those nodes containing CASPR2 or Kv1.1 comparing between treatment groups (Figures S4I, S4J, S4L, and S4M). However, when CASPR2 and Kv1.1 immunostaining was assessed there was a significant reduction in the immunopositive area in those animals treated with patient IgG when compared to controls (Figures 2I, 2J, S4N, and S4O). Therefore, it seems that the pain-related phenotype seen in mice treated with patient IgG was not due to any gross structural injury or inflammation of the peripheral or CNS. The patient autoantibodies did, however, reduce the levels of CASPR2 and Kv1.1 protein found clustered in the JXP of axons. This reduction was observed despite the fact that we did not detect direct binding of patient-IgG to the JXP *in vivo*; we did observe patient-IgG binding at the level of the neuronal cell body within the DRG that could deplete the total protein available. Given the lack of Ab-mediated injury, we hypothesized that CASPR2-Ab may regulate sensory function by reducing CASPR2 protein levels leading to increased excitability in peripheral sensory neurons.

CASPR2 Expression in DRG Neurons

Because CASPR2-Abs were restricted to the periphery and clearly deposited in the DRG, we initially analyzed the expression of CASPR2 within sensory neuron sub-types. RNA *in situ* hybridization (ISH) showed that >99% of mouse primary sensory neurons from lumbar DRG (defined as those cells that had a signal greater than the mean background signal plus 2 SDs) expressed CASPR2 mRNA (Figure S5A). It was clear, however, that sensory neurons expressed CASPR2 to varying degrees. Large diameter DRG cells expressed higher levels of CASPR2 than small diameter DRG cells, consistent with its known expression within myelinated nerve fibers (Poliak et al., 2003) (Figure S5B). A combination of ISH and immunohistochemistry analyses showed that the highest level of CASPR2 expression was in cells that express NF200 (a marker of myelinated afferents including Aβ and Aδ afferent mechanoreceptors) (Figures S5C and S5D). Peptidergic and non-peptidergic small diameter afferents (principally C-fiber nociceptors) can be identified through expression of CGRP and binding of the lectin IB4, respectively. Both of these populations clearly expressed CASPR2, albeit at a lower level than the NF200 population (Figures S5C and S5D). Very low levels of expression were seen in neurons immunopositive for tyrosine hydroxylase (TH), a marker of non-nociceptive C-fibers (Figures S5C and S5D).

CASPR2 Regulates Pain-Related Hypersensitivity in Mice

We next investigated whether a loss of CASPR2 might alter pain-related behavior using mice that no longer express the full-length (FL) version of CASPR2 (*Cntnap2*$^{-/-}$) (Poliak et al., 2003). Although a short isoform lacking the majority of the extracellular domain is still expressed in these mice (Figure 5F) (Chen et al., 2015), we found that the loss of FL-CASPR2 resulted in pain-related hypersensitivity (Figure 3). *Cntnap2*$^{-/-}$ mice were hypersensitive to von Frey hair application, demonstrating a significantly lower withdrawal threshold compared to wild-type (WT) littermates (*Cntnap2*$^{+/+}$ 0.64 ± 0.06 g versus *Cntnap2*$^{-/-}$ 0.37 ± 0.04 g; Figure 3A). *Cntnap2*$^{-/-}$ mice were also hypersensitive to noxious pinprick application, which causes a rapid reflex withdrawal response mediated by Aδ fibers (Arcourt et al., 2017) (Figure 3B). We also assessed the response to dynamic mechanical stimuli, but saw no differences between genotypes (Figure 3C). There was no difference in withdrawal latency to a radiant heat source (Hargreaves method) or the hot plate set at 50°C when comparing *Cntnap2*$^{-/-}$ and control mice (Figures 3D and 3E). However, when the hotplate was set at 53°C there was a significant difference between groups, with *Cntnap2*$^{-/-}$ mice having a shorter latency to response (*Cntnap2*$^{+/+}$ 10.0 ± 0.5 s versus *Cntnap2*$^{-/-}$ 8.3 ± 0.4 s; Figure 3F). This suggests that a loss of CASPR2 results in hypersensitivity to supra-threshold noxious heat. Sensitivity to cold temperatures, measured with a thermal preference paradigm, was unchanged between genotypes (Figure 3G). We also tested mechanical and

Figure 2. Patient CASPR2-Abs Bind *In Vivo* but Do Not Cause Gross Inflammation or Nerve Damage

(A and B) Representative image of a spinal cord (A) and DRG (B) section from a mouse treated with patient IgG. No deposition of human IgG (green) seen in the spinal cord (A). In the DRG, human IgG binds to sensory neurons (B). Scale bars, 200 μm (A), 50 μm (B).

(C and D) Representative images of sciatic nerve (C) and DRG (D) sections from mice treated with either control or patient 2 IgG stained for IBA1 (red). Quantification shows no difference between treatment groups; n = 4 mice. Scale bar, 50 μm.

(E and F) Representative images of DRG sections from mice treated with either control or patient 2 IgG stained for Ly6G (red, E) and CD3 (red, F). The number of positive cells was very low, and no difference was found between groups; n = 4 mice. Scale bar, 25 μm.

(G) Representative images of mouse glabrous skin. PGP9.5 (green) was used to mark nerve fibers. No difference was seen in the IENFD between treatment groups; n = 5 mice. Scale bar, 25 μm.

(H) Representative images of mouse DRG sections stained for the injury marker ATF3 (red). Quantification showed no difference between treatment groups; n = 4 mice. Scale bar, 50 μm.

(I and J) High-power representative images of single nodes (marked by CASPR [red]) from mouse sciatic nerve (I). Kv1.1 (green, top) and CASPR2 (green, bottom) staining is reduced in mice treated with patient 2 IgG. Quantification shows a significant reduction in the area of both Kv1.1 and CASPR2 staining in the patient IgG group versus control (J); n = 4 mice.

Data shown as mean ± SEM, *p < 0.05 versus control IgG group. See also Figures S2–S4.

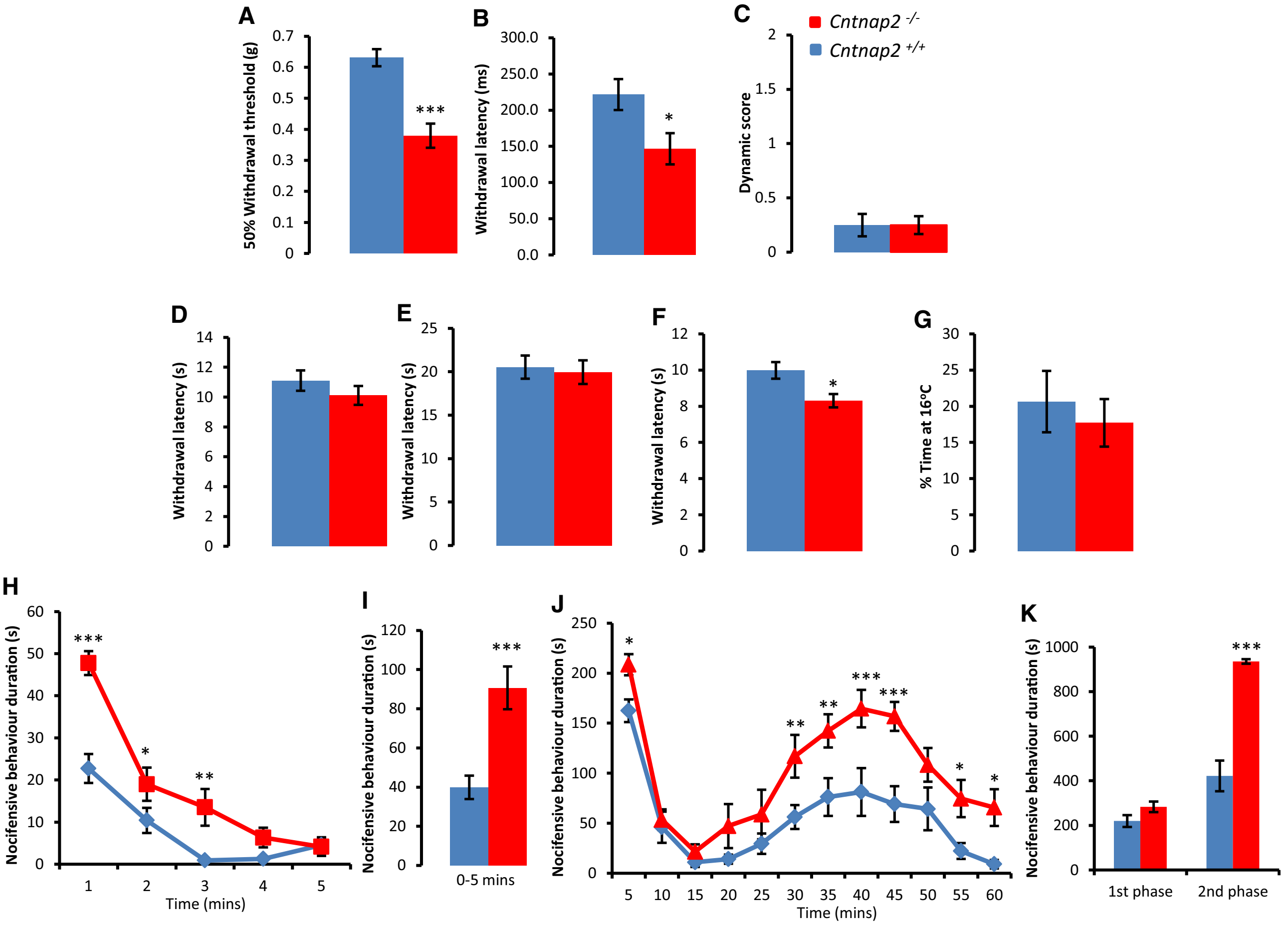

Figure 3. CASPR2 Regulates Pain-Related Hypersensitivity in Mice

(A) Using von Frey hairs, *Cntnap2*$^{-/-}$ (n = 15) mice display hypersensitivity to mechanical stimuli when compared to WT littermates (*Cntnap2*$^{+/+}$, n = 20).

(B) Withdrawal latency to pinprick application is significantly reduced in *Cntnap2*$^{-/-}$ (n = 14) compared to WT littermates (*Cntnap2*$^{+/+}$, n = 12).

(C) No difference in dynamic allodynia measured following brush application to the hindpaw between genotypes (*Cntnap2*$^{-/-}$, n = 8; *Cntnap2*$^{+/+}$, n = 6).

(D–F) *Cntnap2*$^{-/-}$ mice (n = 8) do not display heat hypersensitivity to threshold stimuli as measured by the Hargreaves test (D) or to the hot plate set at 50°C (E). However, when using the hot plate set at 53°C (F), *Cntnap2*$^{-/-}$ mice have a reduced latency to withdrawal compared to *Cntnap2*$^{+/+}$ mice (n = 13).

(G) In comparison to WT littermates, *Cntnap2*$^{-/-}$ mice do not display cold hypersensitivity as measured by the thermal preference test; n = 7 in both groups.

(H and I) In response to an intraplantar injection of capsaicin (1.5 μg), the duration of pain-related behavior is significantly greater in *Cntnap2*$^{-/-}$ (n = 11) versus *Cntnap2*$^{+/+}$ (n = 12) mice over a 5-min period (I), but particularly in the first minute (H).

(J and K) In comparison to control mice, *Cntnap2*$^{-/-}$ mice have an increased response to 5% formalin. This difference is significant in the first 5 min after injection of formalin (J), as well as in the second phase of the behavioral response (K); n = 8 for both groups.

Data shown as mean ± SEM, *p < 0.05, **p < 0.01, ***p < 0.001 versus *Cntnap2*$^{+/+}$ group. See also Figures S5 and S6.

thermal sensitivity in mice heterozygous for the loss of FL-CASPR2 (*Cntnap2*$^{+/-}$). Thresholds were not significantly altered when compared to *Cntnap2*$^{+/+}$ using von Frey hairs, the Hargreaves method, or the hot plate set at 53°C (*Cntnap2*$^{+/-}$ 0.58 ± 0.04 g, 11.1 ± 0.43 s, 8.9 ± 0.7 s, respectively; n = 7).

Sensitivity to chemical algogens was also assessed. In *Cntnap2*$^{-/-}$ mice, intraplantar injection of capsaicin produced a significantly augmented pain response versus WT (Figures 3H and 3I). Loss of FL-CASPR2 also resulted in enhanced nocifensive responses during the formalin test, both in the first (0–15 min) and second phase (15–60 min) post-injection (Figures 3J and 3K). The initial response (0–5 min) in *Cntnap2*$^{-/-}$ was significantly greater than in mice expressing FL-CASPR2 (*Cntnap2*$^{+/+}$ 162.5 ± 11.3 s versus *Cntnap2*$^{-/-}$ 208.5 ± 10.5 s; Figure 3J). This difference subsided, but became evident again in the second phase (Figures 3J and 3K). Assessment of c-*fos* in the spinal cord did not reveal any difference between genotypes (Figures S6A and S6B), suggesting that the increased behavioral response in the second phase may be driven by increased primary afferent activity (Fischer et al., 2014). Without the application of a chemical algogen, no nocifensive behavior was seen in *Cntnap2*$^{-/-}$ mice. We also studied proprioception and motor behavior using the beam test, accelerating rotarod, and open field test (Table S3). There were no significant differences in *Cntnap2*$^{-/-}$ mice compared to controls (Table S3). However, mice lacking

FL-CASPR2 had a significantly longer latency to fall on a constantly moving rotarod (Table S3), suggesting they also display some degree of motor hyperactivity in line with previous findings (Peñagarikano et al., 2011).

Using immunohistochemical markers, we saw no differences in the populations of sensory neuron subtypes within the DRG or IENFD (where the majority of fibers are nociceptors) from *Cntnap2*$^{-/-}$ mice compared to control (Figures S6C–S6F). Given previous reports of a reduced number of cortical inhibitory interneurons in *Cntnap2*$^{-/-}$ mice (Peñagarikano et al., 2011), we looked at CASPR2 expression within the spinal cord and saw that many Pax2-positive neurons (a marker of inhibitory interneurons) expressed CASPR2 (Figures S6G and S6H). We also confirmed its expression within inhibitory interneurons using glycine transporter 2-EGFP reporter mice (Zeilhofer et al., 2005) (Figure S6I). Quantification of Pax2 interneurons in the dorsal horn of the spinal cord, however, showed that numbers were unchanged between genotypes (Figures S6J and S6K) and that there was no difference in the number of inhibitory synapses (Figures S6L and S6M). We also assessed gene expression in the DRG using qPCR and found no difference in the transcription of a number of pain-related genes (Table S4). Therefore, *Cntnap2*$^{-/-}$ mice display pain-related hypersensitivity with no major anatomical or transcriptional changes at the level of the DRG or spinal cord.

CASPR2 Regulates Sensory Neuronal Excitability and Membrane Kv1 Channel Expression

We next used *in vivo* intracellular calcium imaging as a measure of primary sensory neuron activity to see whether CASPR2 impacted neuronal excitability at the level of the cell soma. *Cntnap2*$^{-/-}$ mice and WT littermates were given intrathecal injections of an AAV9 delivery vector encoding the calcium indicator GCaMP6. DRG neuronal activity was indicated by a change in fluorescent signal from baseline levels that occurred following the application of sensory stimuli to the hindpaw (Figure 4A). For our analysis, we separated neuronal responses by cell profile area and found that on average both small- (<500 μm^2) and medium (500–1,000 μm^2)-sized DRG neurons from *Cntnap2*$^{-/-}$ mice exhibited larger increases in intracellular calcium following sensory stimulation. Medium-sized DRG neurons were significantly hyper-responsive to mechanical stimulation, both brush and noxious pinch, applied to the hindpaw when compared to controls (Figures 4B and 4C). Small-sized DRG neurons from *Cntnap2*$^{-/-}$ were also more responsive to pinch application, as well as noxious heat (Figure 4D). We also measured the activity of sensory neurons following capsaicin application and observed that a population of medium-sized neurons from *Cntnap2*$^{-/-}$ was activated by capsaicin in contrast to zero cells from WT mice (Figure 4E). Calculation of the median cell profile size of a capsaicin responder showed this had shifted from 234.8 μm^2 in controls to 438.8 μm^2 in *Cntnap2*$^{-/-}$ mice with a statistically significant shift in the population distribution ($p < 0.0001$, Kolmogorov-Smirnov test). We also looked at spontaneous activity in DRG neurons prior to application of sensory stimuli and found that there was no difference between genotypes (*Cntnap2*$^{+/+}$ 8.5% ± 2.6% versus *Cntnap2*$^{-/-}$ 7.3% ± 1.1%, $p > 0.05$, Student's t test). These findings are in agreement with the behavioral phenotype (Figure 3), suggesting that CASPR2 can regulate excitability at the level of the DRG, and we therefore used patch-clamp analysis to assess neuronal excitability of dissociated DRG cells.

In line with *in vivo* calcium imaging, we found that both small- (diameter <25 μm) and medium-sized DRG neurons (diameter 25–35 μm) from *Cntnap2*$^{-/-}$ mice had significantly lower rheobases than controls (*Cntnap2*$^{+/+}$ 172.6 ± 14.9 versus *Cntnap2*$^{-/-}$ 133.4 ± 10.9 and *Cntnap2*$^{+/+}$ 633.6 ± 66.6pA versus *Cntnap2*$^{-/-}$ 446.5 ± 52.1 pA, respectively) (Figure 5A) consistent with enhanced excitability. This was not true of large (>35 μm) diameter neurons (Figure 5A). When measuring firing in response to longer (500 ms) current injections of increasing magnitude, we noted that small- and medium-sized *Cntnap2*$^{-/-}$ neurons activated at lower thresholds and that medium neurons fired more action potentials in response to supra-threshold stimulation compared to *Cntnap2*$^{+/+}$ neurons (Figure 5B). Resting biophysical properties in these neurons were normal (Table S5).

Kv1 channels contribute to I_{KD}, a slowly inactivating voltage-dependent potassium current that activates at potentials more negative than the action potential threshold (Madrid et al., 2009), positioning these channels as important regulators of neuronal excitability. Due to their interaction with CASPR2, we hypothesized that effects on these channels may underlie the hyperexcitability phenotype observed in *Cntnap2*$^{-/-}$ neurons. We focused on potassium currents in medium diameter DRG neurons as the hyperexcitability was most apparent in these neurons. *Cntnap2*$^{+/+}$ neurons displayed a slowly inactivating outward current upon membrane potential depolarization that was highly sensitive to α-dendrotoxin (DTX, a selective Kv1 channel inhibitor) and therefore represents I_{KD} carried partially by Kv1 channels (Madrid et al., 2009). I_{KD} was significantly reduced in medium diameter *Cntnap2*$^{-/-}$ neurons compared control (Figure 5C). When tested at +40 mV, the DTX-sensitive component of this current was also reduced in *Cntnap2*$^{-/-}$ neurons (Figure 5C) (*Cntnap2*$^{+/+}$ 143.8 ± 17.1 pA/pF versus *Cntnap2*$^{-/-}$ 78.6 ± 10.8 pA/pF, $p < 0.01$, Student's t test), suggestive of decreased Kv1 current. One possibility for the decrease in sensitivity to DTX is that CASPR2 is important not only for the longitudinal movement of these potassium channels along axons, but also their trafficking to the cell membrane, something it has been shown to do for other ion channels (Varea et al., 2015). We therefore assessed the expression of Kv1.2 in culture and found clear membrane staining of Kv1.2 in DRG neurons from *Cntnap2*$^{+/+}$ mice that is lost following ablation of FL-CASPR2 (Figure 5D). Profile plots show the intensity of Kv1.2 immunoreactivity across the cell body and were used to define positive membrane expression (Figure 5D). Quantification of these showed that 25.8% ± 3.9% of total DRG neurons from control mice were defined as having Kv1.2 membrane expression (Figure 5E). In agreement with electrophysiological findings, Kv1.2 expression was significantly reduced to 11.3% ± 2.5% of DRG neurons in *Cntnap2*$^{-/-}$ mice (Figure 5E). Note that the mRNA expression of *Kcna2* (and *Kcna1*) in DRG did not differ between *Cntnap2*$^{+/+}$ and *Cntnap2*$^{-/-}$ mice (Table S4).

We also investigated whether CASPR2 overexpression could reverse hyperexcitability in medium DRG neurons. We observed that in comparison to acutely cultured WT DRG neurons (1 day *in vitro* [DIV]), WT neurons cultured for 5 DIV lost their Kv1.2

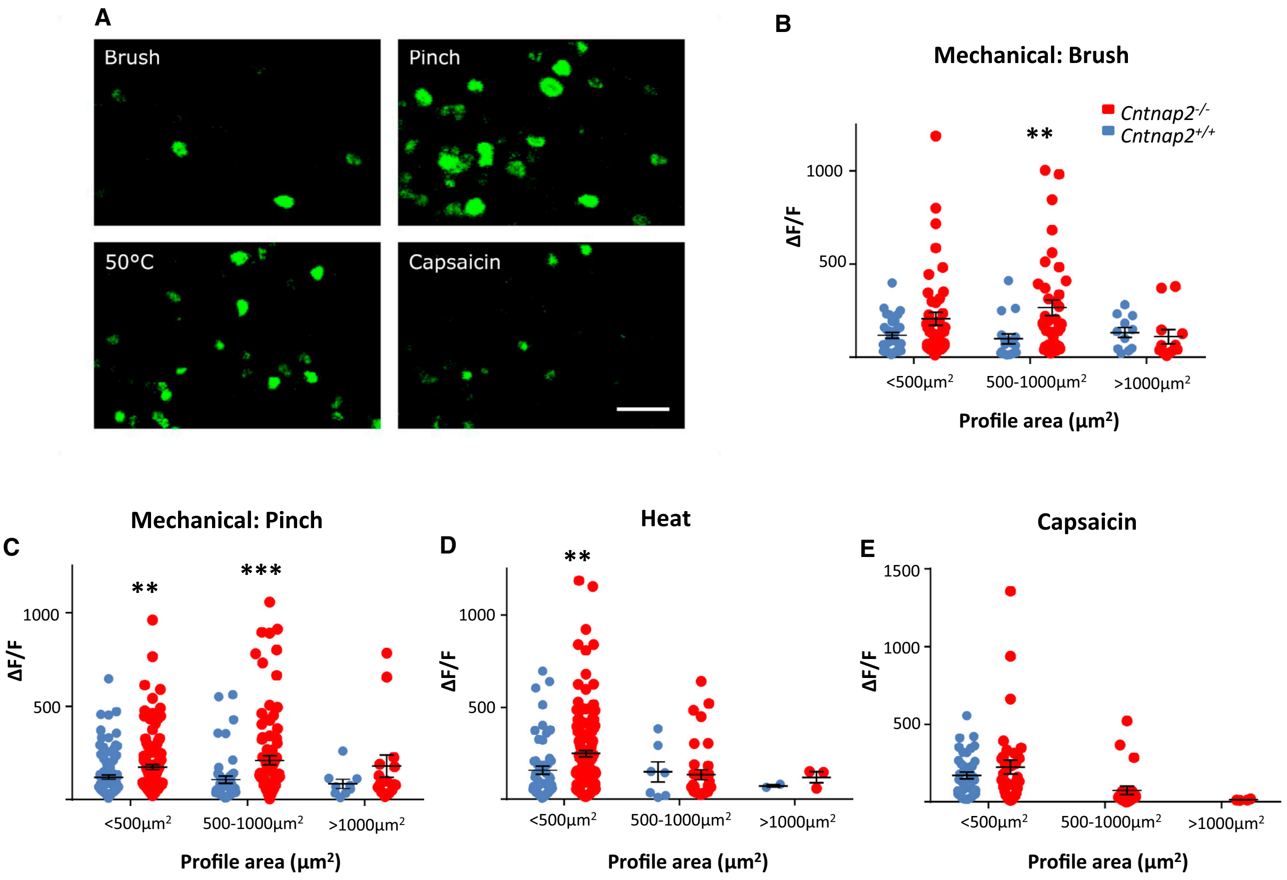

Figure 4. *In Vivo* Calcium Imaging Shows that DRG Neurons from *Cntnap2*$^{-/-}$ Mice Are Hyper-Responsive to Mechanical and Chemical Stimuli

(A) Representative images of GCaMP6 fluorescence as a measure of intracellular calcium following stimulation to the hindpaw. Scale bar, 100 μm.

(B) In comparison to control, DRG neurons from *Cntnap2*$^{-/-}$ mice had a significantly greater response to brush stimulation applied to the glabrous skin. This was particularly true of medium-sized cells (500–1,000 μm^2; *Cntnap2*$^{+/+}$, n = 33 cells; *Cntnap2*$^{-/-}$, n = 46 cells).

(C) In response to noxious pinch stimulation, there was also a significant increase in the response of DRG neurons from *Cntnap2*$^{-/-}$ mice in both small- and medium-sized neurons (<500 μm^2 *Cntnap2*$^{+/+}$, n = 106 cells; *Cntnap2*$^{-/-}$, n = 132 cells; 500–1,000 μm^2 *Cntnap2*$^{+/+}$, n = 46 cells; *Cntnap2*$^{-/-}$, n = 93 cells).

(D) In response to noxious heat stimulation (50°C), there was a significant increase in the response of DRG neurons from *Cntnap2*$^{-/-}$ mice in small DRG neurons (<500 μm^2 *Cntnap2*$^{+/+}$, n = 54 cells; *Cntnap2*$^{-/-}$, n = 143 cells).

(E) No statistically significant difference was seen in the response of small cells to capsaicin application (<500 μm^2; *Cntnap2*$^{+/+}$, n = 41 cells; *Cntnap2*$^{-/-}$, n = 36 cells). Note the number of medium-sized responders: *Cntnap2*$^{+/+}$ mice = 0, *Cntnap2*$^{-/-}$ mice = 23.

Cells analyzed from four *Cntnap2*$^{+/+}$ and five *Cntnap2*$^{-/-}$ mice. Data shown as mean ± SEM, **$p < 0.01$, ***$p < 0.001$ versus *Cntnap2*$^{+/+}$ group.

membrane expression (Figure S7A) and became hyperexcitable, with a significant reduction in their rheobase (Figure 5G); interestingly, we also noted reduced mRNA expression of *Cntnap2* over the same time course (Figure S7B). We therefore set out to rescue this phenotype by overexpressing CASPR2. In addition to FL-CASPR2, we studied the effects of the shorter isoform (SH-CASPR2) that lacks most of the extracellular domain (Figure 5F). qPCR showed that SH-CASPR2 is still expressed in DRG from *Cntnap2*$^{-/-}$ mice, but expression in WT DRG is 10-fold lower than that of the main FL-CASPR2 isoform (Figures S7C and S7D). DRG cells were electroporated with plasmids containing either FL-CASPR2 or SH-CASPR2, both tagged with EGFP in the cytoplasmic domain, or an EGFP plasmid control. Overexpression of FL-CASPR2-EGFP resulted in membranous EGFP staining in a subset of DRG neurons (Figure S7E). At 5 DIV, SH-CASPR2-EGFP overexpression had no effect on the rheobase (Figure 5G). By contrast, FL-CASPR2-EGFP overexpressing cells had a significantly higher rheobase than EGFP-expressing cells at the same time point (EGFP 255.8 ± 32.5 pA versus FL-CASPR2-EGFP 605.4 ± 88.9 pA; Figure 5G), and this effect was lost with DTX treatment, suggesting that it was due to enhanced activity of Kv1 channels (Figure 5I). Overexpression of SH-CASPR2 had no effect on I_{KD} (Figure 5H), whereas I_{KD} was greater in the FL-CASPR2-EGFP versus EGFP cells after 5 DIV and was similar to the levels seen after 1 DIV (Figure 5H). Again, the effect was significantly prevented by DTX (Figure 5J). The lack of effect of SH-CASPR2 is most likely due to its diminished ability to be expressed at the membrane (Figure S7F). Interestingly, patient CASPR2-Ab only bound to the FL-CASPR2 isoform, and not the SH-CASPR2 isoform (Figures S8A–S8C). These data therefore suggest that

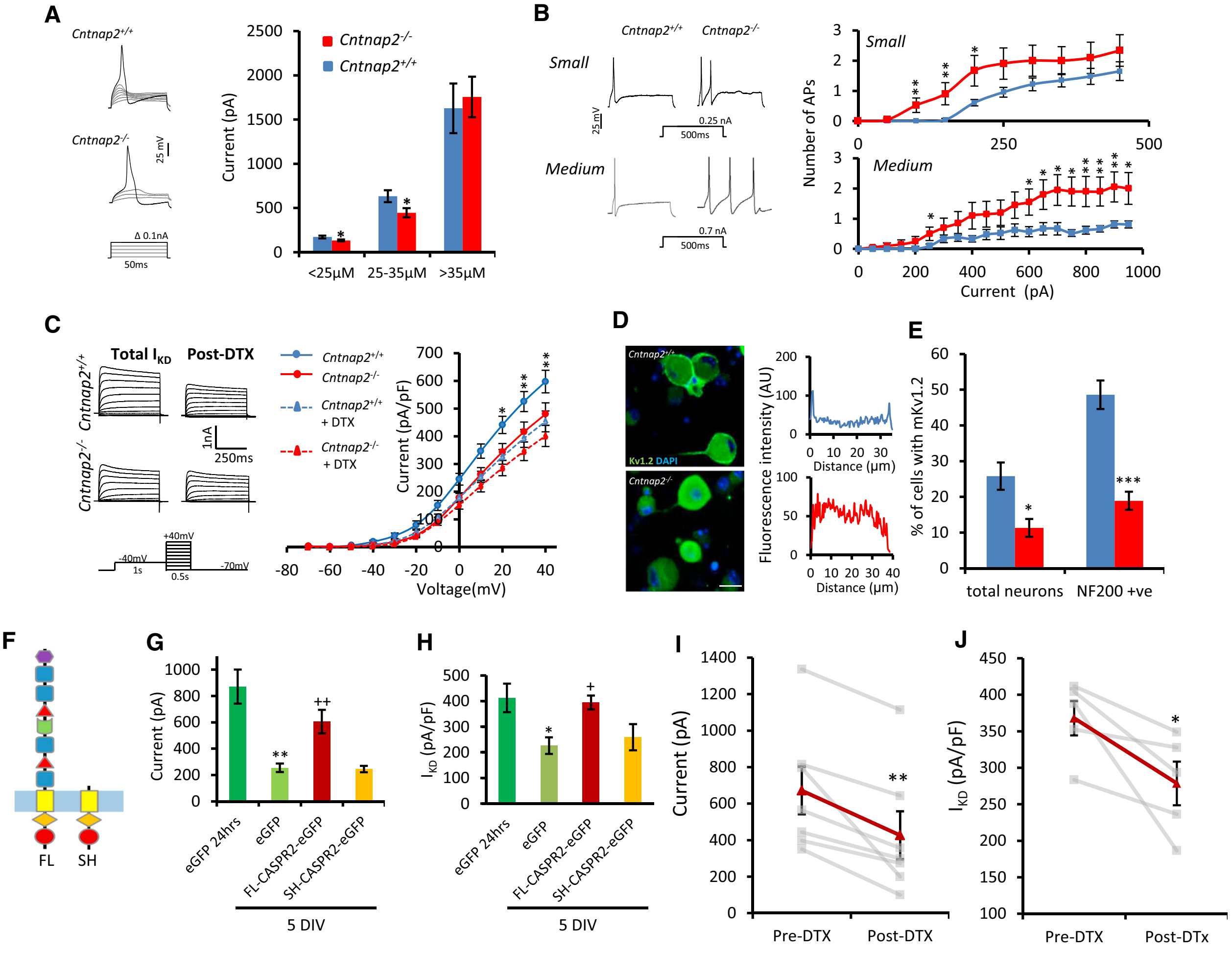

Figure 5. CASPR2 Regulates the Excitability of DRG Neurons

(A) Representative traces showing action potential firing to short incremental current injection in medium (25–35 μm) diameter neurons. Small (<25 μm; *Cntnap2*$^{+/+}$, n = 47; *Cntnap2*$^{-/-}$,n = 45 cells) and medium (25–35 μm; *Cntnap2*$^{+/+}$, n = 20; *Cntnap2*$^{-/-}$, n = 21 cells) diameter DRG neurons cultured from *Cntnap2*$^{-/-}$ mice have a significantly reduced rheobase when compared to neurons from control mice. There were no differences between genotype in large diameter neurons (*Cntnap2* $^{+/+}$, n = 11; *Cntnap2*$^{-/-}$, n = 13 cells).

(B) Representative traces showing action potential firing to supra-threshold prolonged current injection in small (<25 μm) and medium (25–35 μm) diameter neurons. Quantification across a range of current steps showed that both small (*Cntnap2*$^{+/+}$, n = 23 cells; *Cntnap2*$^{-/-}$, n = 21 cells) and medium (*Cntnap2*$^{+/+}$, n = 20; *Cntnap2*$^{-/-}$, n = 21 cells) diameter *Cntnap2*$^{-/-}$ neurons display increased firing frequency in comparison to *Cntnap2*$^{+/+}$ neurons.

(C) Example traces from medium diameter neurons of outward currents evoked by depolarizing pulses. I_{KD} was measured pre- and post-application of 100 nM DTX. Current voltage relationships for I_{KD} demonstrating increased current in *Cntnap2*$^{+/+}$neurons (n = 12 cells) compared to *Cntnap2*$^{-/-}$neurons (n = 14 cells) that was not present following DTX treatment.

(D) Representative images showing Kv1.2 membrane staining in DRG neurons from *Cntnap2*$^{-/-}$ and *Cntnap2*$^{+/+}$ mice. Scale bar, 25 μm. Profile plots were used to define membrane staining.

(E) Cultured DRG neurons from *Cntnap2*$^{-/-}$ mice have less Kv1.2 membrane staining when compared to control neurons. β-III-tubulin used to mark all neurons (n = 4 coverslips from two independent experiments).

(F) Diagram highlighting the difference in the extracellular domain between the full-length (FL) and the short (SH) CASPR2 isoform.

(G) After 5 DIV, there is a significant reduction in rheobase (EGFP 1 DIV [n = 12 cells] versus EGFP 5 DIV [n = 16 cells]) for WT neurons transfected with a plasmid containing EGFP only. FL-CASPR2-EGFP (n = 25 cells) overexpression at 5 DIV restores the rheobase to 1DIV measurements. However, overexpression of the short isoform (SH-CASPR2-EGFP) does not affect rheobase values (n = 9 cells).

(H) After 5 DIV, there is a significant reduction in I_{KD} (EGFP 1 DIV versus EGFP 5 DIV) for WT neurons transfected with a plasmid containing EGFP only (EGFP 1 DIV, n = 16 cells; EGFP 5 DIV, n = 13 cells). FL-CASPR2-EGFP overexpression at 5 DIV (n = 12 cells) restores the I_{KD} to 1 DIV levels. Overexpression of SH-CASPR2-EGFP did not affect I_{KD} (n = 12 cells).

(I and J) The restoration of rheobase (I) and I_{KD} (J), due to the overexpression of FL-CASPR2-EGFP at 5 DIV is reduced by the application of DTX. Gray lines show individual cells before and 5 min after the application of 100 nM DTX. Red lines show the average (rheobase, n = 7 cells; I_{KD}, n = 5 cells).

Data shown as mean ± SEM. For (A)–(C), *p < 0.05, **p < 0.01, ***p < 0.001 versus *Cntnap2*$^{+/+}$ group. For (G) and (H), *p < 0.05, **p < 0.01 versus EGFP 24 hr and +p < 0.05, ++p < 0.01 versus EGFP 5 DIV. For (I) and (J), *p < 0.05, **p < 0.01 versus pre-DTX.

See also Figure S7.

the FL isoform of CASPR2 has a cell-autonomous effect on a population of small-/medium-sized DRG cells through its regulation of Kv1 channel membrane expression.

CASPR2 Is Required for Normal D-Hair Primary Afferent Excitability

In order to study the effects of CASPR2 on the transduction properties of peripheral sensory terminals in the hindpaw glabrous skin, we used the tibial nerve-skin nerve preparation (Milenkovic et al., 2014) and, given the behavioral findings from both the CASPR2-Ab-treated and *Cntnap2*$^{-/-}$ mice, focused on mechanotransduction. Evoked responses to mechanical and electrical stimuli were assessed. We recorded from Aβ low-threshold rapidly adapting and slowly adapting mechanoreceptors (RAM and SAM, respectively), Aδ low-threshold mechanoreceptors (D-hair afferents; note that these are present *in plantar* paw skin; see Discussion), Aδ mechano-nociceptors (A-Ms), C-mechano-nociceptors (C-Ms), and polymodal C-mechano-heat-nociceptors (C-MHs) (Figure 6A). Consistent with previous findings in *Cntnap2*$^{-/-}$ mice (Poliak et al., 2003), we did not find changes in the conduction velocities of any primary afferent subtype in mice lacking FL-CASPR2 compared to *Cntnap2*$^{+/+}$ mice (Figure 6B). When using an increasing velocity or an increasing force protocol (STAR Methods), we observed no changes in the stimulus-response functions of RAMs, SAMs, A-Ms, C-Ms, or C-MHs (Figure 6C; Table S6). We did, however, find markedly enhanced excitability in D-hair primary afferents. The mechanical stimulation protocol for D-hairs consisted of a ramp and hold phase and the typical response of a D-hair from *Cntnap2*$^{+/+}$ and *Cntnap2*$^{-/-}$ mice is shown in Figure 6D. As D-hairs characteristically respond only to moving stimuli, we initially analyzed the velocity stimulus-response function during the ramp phase only (Figure S9A). D-hair primary afferents are exquisitely sensitive to slow-moving stimuli (Lechner and Lewin, 2013). *Cntnap2*$^{-/-}$ D-hairs show significant hyperexcitability to slow stimulus velocities compared to *Cntnap2*$^{+/+}$ D-hairs (Figures 6E, S9B, and S9C). We next analyzed the firing frequency of D-hairs during the entire stimulus. Compared to *Cntnap2*$^{+/+}$mice, D-hairs from *Cntnap2*$^{-/-}$ mice showed markedly impaired adaption and continued to fire during the static phase of the stimulus at velocities of 75 μm/s (Figure 6F) and 150 μm/s (Figure 6G). It is unusual for D-hair mechanoreceptors to fire during the hold phase of the stimulus. However, the *Cntnap2*$^{-/-}$ D-hair firing frequency was strikingly and significantly increased during the stimulus hold phase compared to *Cntnap2*$^{+/+}$ D-hairs (Figures 6H and 6I). On average, only 24.29% ± 6.0% of WT D-hairs responded during the hold phase of the stimulus while 60.0% ± 7.95% of *Cntnap2*$^{-/-}$ D-hairs responded (Figure 6J). Finally, to rule out a developmental role of CASPR2 on target innervation, we assessed D-hair innervation in both glabrous and hairy skin (Figures S9D and S9E). TrkB is expressed by low-threshold mechanosensitive afferents and can be used to mark D-hair lanceolate endings in the skin (Li et al., 2011). We saw no differences between genotypes in the number of TrkB-positive lanceolate endings in both glabrous and hairy skin (Figures S9D and S9E). The loss of CASPR2, therefore, alters the stimulus response of a surprisingly selective population of primary afferent terminals, the D-hairs, resulting in enhanced responses to low-threshold mechanical stimuli and markedly impaired adaption.

Loss of CASPR2 Leads to Dorsal Horn Hyperexcitability

Given the integration of sensory inputs at spinal level and potential synaptic role of CASPR2, we investigated the role of CASPR2 in dorsal horn processing of nociceptive stimuli. We performed *in vivo* extracellular recordings from wide dynamic range (WDR) dorsal horn neurons. Neurons were characterized from depths corresponding to the deep dorsal horn laminae (*Cntnap2*$^{+/+}$, 650 ± 23 μm; *Cntnap2*$^{-/-}$, 602 ± 89 μm) and responded to mechanical and thermal stimuli in an intensity-dependent manner.

The reduced mechanical withdrawal thresholds observed in behavioral assays were supported by enhanced neuronal response to punctate mechanical stimuli in *Cntnap2*$^{-/-}$ mice compared to *Cntnap2*$^{+/+}$, most notably to noxious intensities of stimulation (Figure 7A). Heat-evoked responses were less affected by loss of CASPR2 with heat hypersensitivity only observed at supramaximal noxious intensities of stimulation (Figure 7B). This is consistent with the behavioral findings (Figure 3). Although there was a trend toward increased neuronal responses in *Cntnap2*$^{-/-}$ mice, we did not observe a statistically significant difference compared to control in response to dynamic brush stimulation of the receptive field (Figure 7C). No differences were observed between genotypes following application of innocuous (acetone) or noxious (ethyl chloride) evaporative cooling (Figure 7D). Receptive field maps were produced in response to a pinch stimulus applied to the skin; neurons characterized in *Cntnap2*$^{-/-}$ mice exhibited similar receptive field sizes to neurons from WT littermates (Figure 7E). Electrical stimulation of the receptive field revealed reduced thresholds in *Cntnap2*$^{-/-}$ mice for the activation of A- and C-fibers (Figure 7F). A train of supra-threshold electrical stimuli (3xC-fiber threshold) was delivered to the receptive field, and neurons from *Cntnap2*$^{+/+}$ and *Cntnap2*$^{-/-}$ mice exhibited comparable levels of wind-up (the potentiated response and measure of postsynaptic hyperexcitability) (Figures 7G–7I). The cumulative total of neuronal events evoked by both A- and C-fibers was increased in *Cntnap2*$^{-/-}$ mice compared to *Cntnap2*$^{+/+}$ (Figure 7G). Furthermore, the input (the non-potentiated response more indicative of pre-synaptic events) and post-discharge, a property of spinal neurons, were both elevated in *Cntnap2*$^{-/-}$ mice (Figure 7G). These data demonstrate hyperexcitability within the pain-signaling pathway at the level of the spinal cord in *Cntnap2*$^{-/-}$; the modality-specific changes, coupled with alterations in input, but not wind-up, suggest a pre- rather than post-synaptic locus of action for CASPR2 in regulating dorsal horn hyperexcitability. To further address this, we have used spinal cord slice preparations and recorded from dorsal horn neurons in Lamina II. We did not see any evidence of altered synaptic function due to loss of FL-CASPR2. There was no significant difference in the frequency or amplitude of either spontaneous or capsaicin-evoked excitatory postsynaptic currents (EPSCs) between genotypes (Figures S10A–S10C). These findings, coupled with primary afferent hyperexcitability seen in Figures 4, 5, and 6, strongly suggest that the increased response measured in WDR neurons from *Cntnap2*$^{-/-}$ mice has a peripheral locus of action.

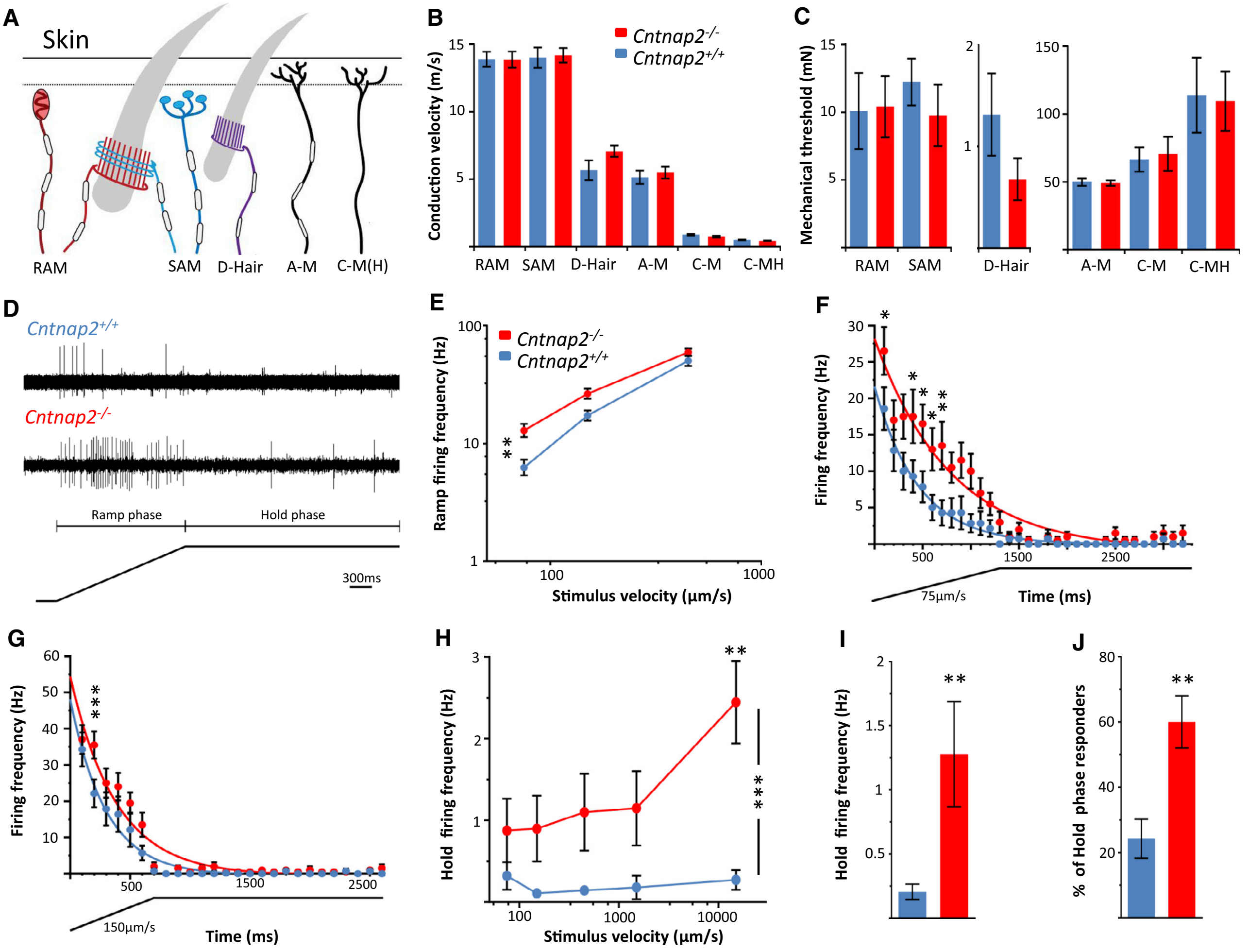

Figure 6. Genetic Deletion of FL-CASPR2 Results in Hyper-Excitable D-Hair Primary Afferents

(A) Diagram illustrating the cutaneous mechanoreceptor sensory endings that were identified and recorded from using the *ex vivo* skin nerve preparation

(B and C) Conduction velocity (B) and mechanical thresholds (C) were recorded from mechanoreceptors and nociceptors. No differences were observed between genotypes. See Table S6 for number of recorded units.

(D) Example trace of evoked AP response of both *Cntnap2*$^{+/+}$ (top) and *Cntnap2*$^{-/-}$ (bottom) D-hairs following a mechanical stimulus consisting of a ramp phase and hold phase.

(E) The stimulus response curve showing that *Cntnap2*$^{-/-}$ D-hairs have a significantly higher ramp firing frequency compared to *Cntnap2*$^{+/+}$ D-hairs at slow stimulus velocities.

(F and G) Whole-stimulus (ramp and hold) firing frequency was analyzed every 100 ms to assess D-hair adaptation. At stimulus velocities 75 μm/s (F) and 150 μm/s (G), *Cntnap2*$^{-/-}$ D-hairs elicited increased firing frequencies and significantly less firing adaptation compared to *Cntnap2*$^{+/+}$ D-hairs. Note ramp hold stimulus below x axis.

(H) D-hair firing frequency was analyzed during the hold phase of each stimulus only. *Cntnap2*$^{-/-}$ D-hairs have a significantly higher firing frequency during the hold phase than control D-hairs.

(I) The average hold firing frequency to a stimulus (independent of velocity) was significantly increased in *Cntnap2*$^{-/-}$ D-hairs.

(J) The average percentage of D-hairs that responded to a stimulus during the hold phase was significantly higher in *Cntnap2*$^{-/-}$ mice compared to controls. For (E)–(J), *Cntnap2*$^{+/+}$ n = 14, *Cntnap2*$^{-/-}$ n = 20 units recorded from 16 *Cntnap2*$^{+/+}$ and 15 *Cntnap2*$^{-/-}$ mice. Data shown as mean ± SEM. *p < 0.05, **p < 0.01, ***p < 0.001 versus *Cntnap2*$^{+/+}$ group.

See also Figure S9.

Patient CASPR2-Abs Cause a Loss of Kv1 Channel Membrane Expression and Hyperexcitability in Sensory Neurons

Finally, we used cultured mouse DRG neurons to see if plasma from CASPR2-Ab-positive patients could affect the properties of these cells in a similar manner to that seen following genetic ablation of FL-CASPR2. At the time of plating, DRG neurons were treated with complement-deactivated plasmas from the healthy control subject, patient 1, or patient 2. Initially, we confirmed the ability of patient Abs to bind sensory neurons *in vitro*. Using the anti-human IgG antibody, we saw membrane staining in patient plasma-treated cells, but not in those treated

CellPress

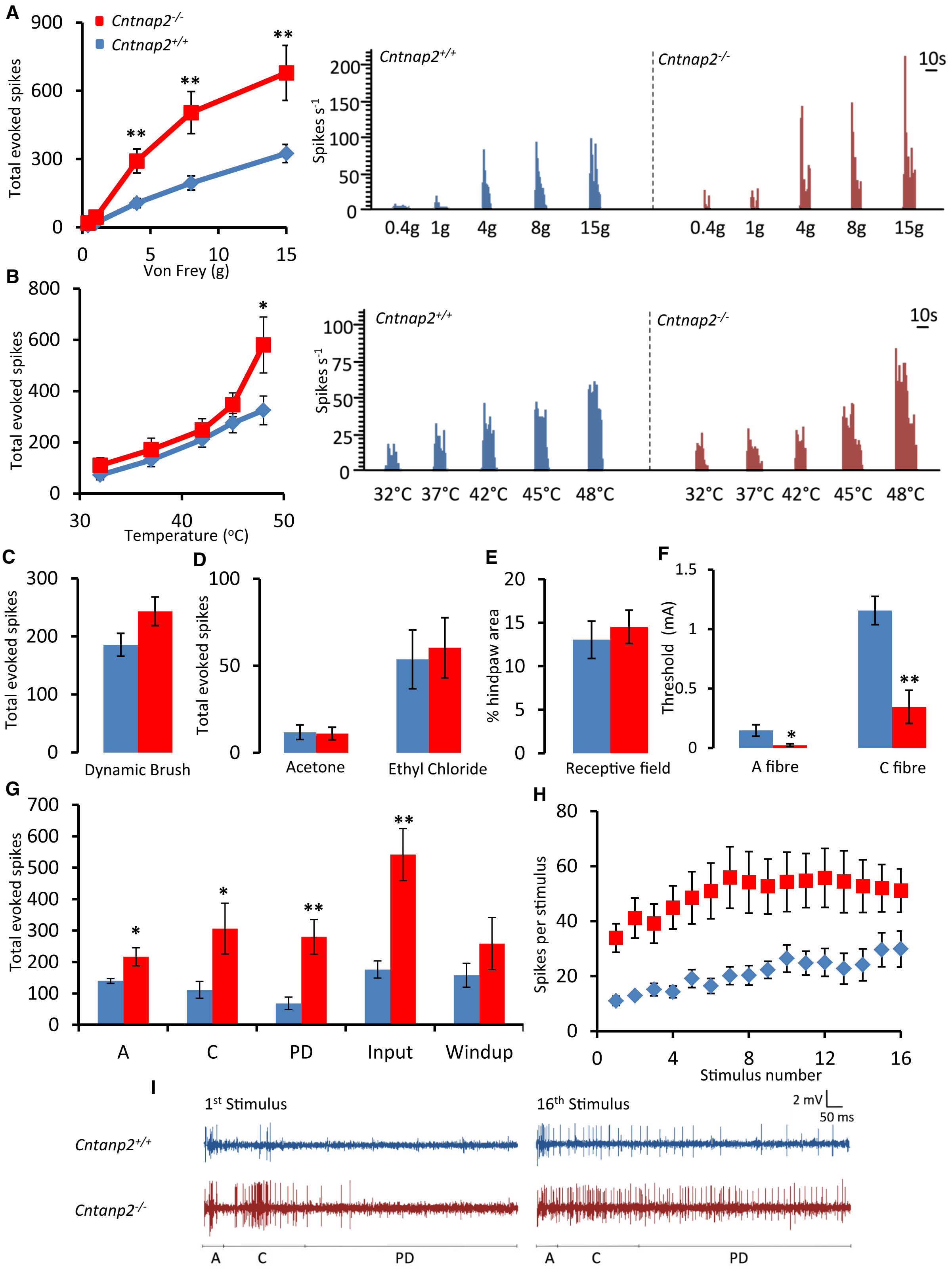

(legend on next page)

with healthy control plasma (Figures S8D–S8F). Furthermore, we confirmed the specificity of these patient autoantibodies for the FL version of CASPR2 by using DRG neurons cultured from *Cntnap2*$^{-/-}$ mice. No binding of patient IgG was seen on DRG neurons lacking FL-CASPR2 (Figure S8G). Patient Ab binding was predominantly seen in NF200-positive cells. In line with experiments carried out on neurons from *Cntnap2*$^{-/-}$, we went on to look at the membrane expression of Kv1.2 in cells treated with plasma for 24 hr at 37°C. Similar to that seen following genetic ablation of FL-CASPR2, there was a significant reduction in the membrane expression of Kv1.2 on DRG neurons treated with patient plasma versus control (control, 35.4% ± 6.4%; patient 1, 12.7% ± 3.6%; patient 2, 17.2% ± 3.5%) (Figures 8A–8C). DRG neurons treated with plasma from both patient 1 and 2 also had a significant reduction in rheobase compared to control (Figure 8D). Furthermore, DRG neurons treated with patient 1 plasma displayed significantly increased repetitive firing in response to supra-threshold stimuli compared to control cells, although this effect was not seen in cells treated with plasma from patient 2 (Figure 8E). These findings suggest that, as in genetic ablation studies, patient CASPR2-Abs increase the excitability of DRG neurons due to reduced Kv1 channel function, and the behavioral hypersensitivity is mediated via enhanced neuronal excitability rather than destructive or pro-inflammatory effects.

DISCUSSION

The immune system is increasingly recognized as making an important contribution to persistent pain states (McMahon et al., 2015). Hitherto, studies of the immune system in neuropathic pain have focused on immune cells and cytokines while the role of autoantibodies in persistent pain is less well established. Autoantibodies against the VGKCC and in particular to CASPR2 have been linked to neuropathic pain and furthermore in some cases pain has been shown to improve following immunotherapy (Klein et al., 2012). These clinical findings, in combination with the phenotype seen in *Cntnap2*$^{-/-}$ mice, suggest that CASPR2-Abs contribute to pain in these patients. We used passive transfer of purified IgG from two patients with high titers of CASPR2-Abs and immunotherapy-responsive pain into mice and found that IgG was deposited in the peripheral, but not central, nervous system (consistent with a peripheral mode of action). Both IgG preparations induced mechanical pain-related hypersensitivity in mice confirming pathogenicity of these antibodies in driving pain-related behavior.

Autoimmune neuropathies such as Guillain-Barre syndrome (Ruts et al., 2012) are associated with a high incidence of neuropathic pain that occurs as a consequence of small fiber injury. We therefore considered whether CASPR2-Abs caused pain-related hypersensitivity as a consequence of direct injury to sensory neurons. We found that the IENFD was unchanged, and there was no evidence of axon loss or demyelination on detailed nerve morphometry. Autoantibodies from patients with rheumatoid arthritis and complex regional pain syndrome can cause abnormal pain behavior in mice through modulation of the inflammatory response and altered production of immune mediators (Tékus et al., 2014; Wigerblad et al., 2016). We did not, however, find evidence of a significant inflammatory response within the peripheral or CNS in mice treated with CASPR2-Abs, and we hypothesized that CASPR2-Abs altered sensory function through the novel mechanism of an acquired channelopathy.

We examined the expression of CASPR2 in sensory neurons and used *Cntnap2*$^{-/-}$ mice to study the effects of CASPR2 levels on pain-related behavior and primary afferent excitability. A recent study assessing a CASPR2 reporter line during embryonic development reported CASPR2 expression in neural circuits sub-serving multiple sensory modalities including somatosensory afferents projecting to footpad skin and the dorsal horn of the spinal cord (Gordon et al., 2016). In adulthood, *Cntnap2* mRNA was broadly expressed by DRG neurons: it is present in small diameter DRG cells with unmyelinated axons with higher expression in those small diameter DRG cells that are presumptive nociceptors (IB4- and CGRP-positive) versus C low-threshold mechanoreceptors (TH-positive) (Li et al., 2011). CASPR2 is expressed at higher levels in medium- to large-sized cells that are NF200 positive. To address the role of CASPR2 in sensory function, we initially studied the response of *Cntnap2*$^{-/-}$ mice, which lack the FL version of CASPR2 (FL-CASPR2), to a diverse range of sensory stimuli. *Cntnap2*$^{-/-}$ mice displayed robust hypersensitivity to mechanical stimuli, enhanced response to supra-threshold noxious heat, and increased nocifensive behavior to the chemical algogens capsaicin and formalin.

Humans with homozygous loss-of-function mutations in *CNTNAP2* demonstrate a number of core features of ASD (Strauss et al., 2006), and somatosensory abnormalities are increasingly recognized in ASD patients (Cascio, 2010). *Cntnap2*$^{-/-}$ mice have been shown to have a number of behavioral features consistent with autism (Peñagarikano et al., 2011). A number of other autism-related genes (*Mecp2*, *Shank3*, or

Figure 7. Increased Activity of Lamina V/VI Dorsal Horn Neurons to Sensory Stimuli *Cntnap2*$^{-/-}$ Mice

(A and B) Evoked neuronal responses to punctate mechanical stimuli (A) are significantly greater in *Cntnap2*$^{-/-}$ (n = 10 cells) compared to *Cntnap2*$^{+/+}$ (n = 11 cells) mice. *Cntnap2*$^{-/-}$ mice also display increased neuronal responses to heat stimuli (B). Histogram traces represent typical single unit responses.

(C and D) No significant differences were seen between evoked neuronal responses to dynamic brush (C) or innocuous (acetone) and noxious (ethyl chloride) evaporative cooling (D).

(E) No differences were seen in the size of the receptive field.

(F) WDR neurons in *Cntnap2*$^{-/-}$ mice display a significantly reduced threshold for both A- and C-fibers following electric stimulation.

(G) *Cntnap2*$^{-/-}$ display an increased neuronal response following electrical stimulation.

(H and I) No difference in the degree of windup was seen between genotypes (H). Representative single-unit traces also shown for the first and last stimulus for both *Cntnap2*$^{-/-}$ and *Cntnap2*$^{+/+}$ (I).

Data shown as mean ± SEM, *p < 0.05, **p < 0.01 versus *Cntnap2*$^{+/+}$ group. Cells recorded from 7 mice per genotype.

See also Figure S10.

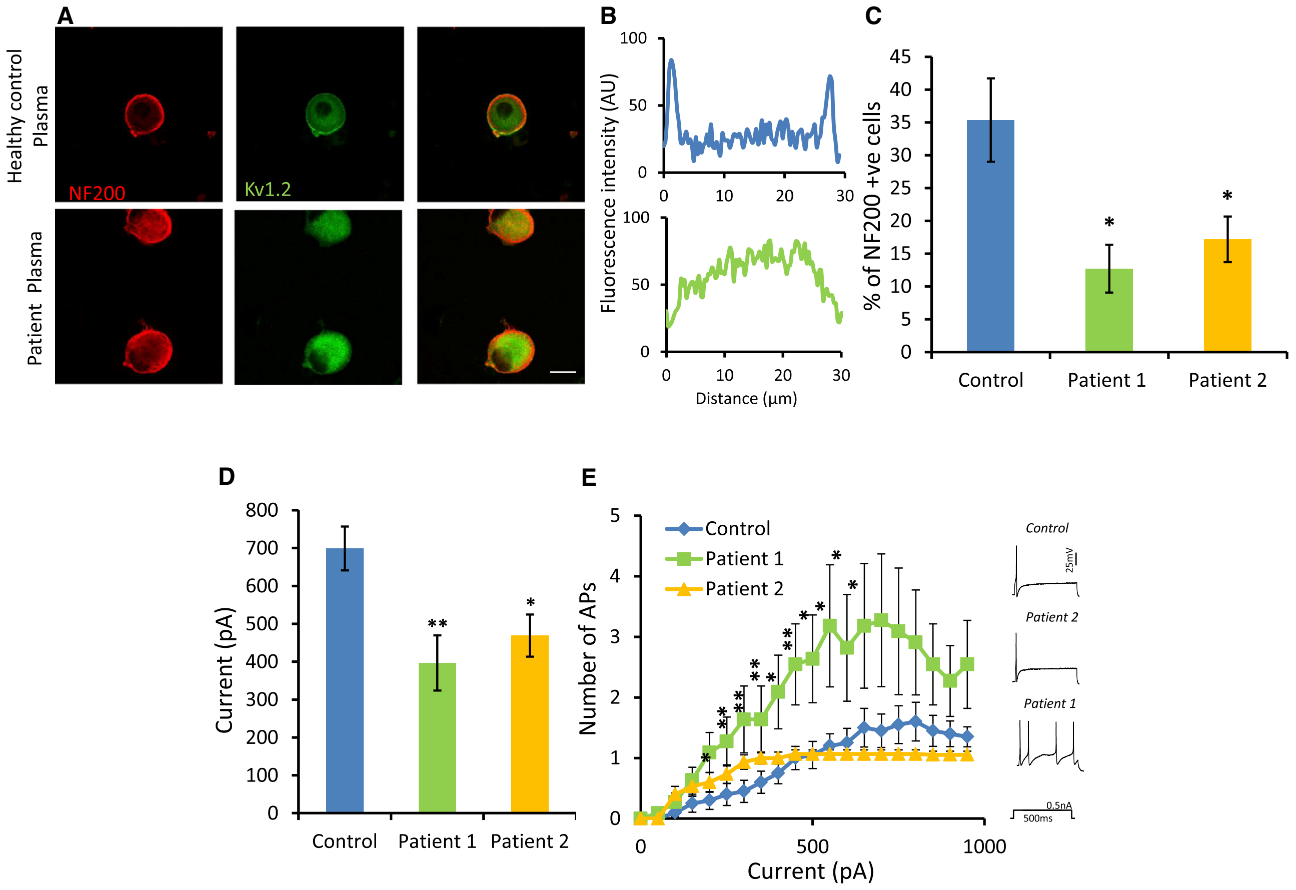

Figure 8. Patient CASPR2-Abs Reduce Kv1 Membrane Expression on DRG Neurons and Increase Their Excitability

(A) Representative images showing that Kv1.2 (green) membrane staining is decreased in NF200-positive (red) DRG neurons treated with plasma from CASPR2-Ab-positive patients when compared to control. Scale bar, 25 μm.

(B) Profile plots showing fluorescent intensity for Kv1.2 immunostaining across the cell. The profile suggests that most of the Kv1.2 is internal following treatment with patient plasma.

(C) Quantification of Kv1.2 membrane staining showing a significant reduction in those cells treated with CASPR2-Ab-positive patient plasma (n = 4 coverslips from two independent experiments).

(D) Medium-sized DRG neurons treated with patient plasma (patient 1, n = 12; patient 2, n = 16 cells) had a significantly reduced rheobase when compared to cells treated with plasma from healthy control (n = 20 cells).

(E) DRG neurons treated with patient 1 (n = 10 cells), but not patient 2 (n = 15 cells), plasma display increased firing frequency in response to prolonged (500 ms) graded inputs of current (0–950 pA) in comparison to controls (n = 20 cells). Representative traces are show for each group.

Data shown as mean ± SEM,$^*p < 0.05$, $^{**}p < 0.01$ versus control group. See also Figure S8.

Fmr1) have recently been shown to contribute to sensory disorders via specific roles within primary sensory neurons (Han et al., 2016; Orefice et al., 2016). Given that genetic or immune-mediated ablation of CASPR2 could cause behavioral hypersensitivity, we examined the role of CASPR2 in regulating excitability within DRG cells.

Using *in vivo* calcium imaging of populations of DRG cells, we found an enhanced response (in both small- and medium-sized cells representing both C and Aδ fibers) to a range of sensory stimuli including noxious heat, brush, pinch, and the algogen capsaicin in *Cntnap2*$^{-/-}$ mice. These data were in agreement with patch-clamp analysis of dissociated DRG cells in which small- and medium-sized neurons demonstrated a reduced rheobase, indicating enhanced excitability. When examining the peripheral terminals of DRG cells in *Cntnap2*$^{-/-}$ mice, we focused on mechanical stimulus-response function given that this was the most striking behavioral finding. We observed changes in mechanosensitivity in a surprisingly selective population of primary afferents, the D-hair mechanoreceptors, which may indicate that CASPR2 has differential effects on DRG cells dependent on the compartment examined (i.e., soma versus peripheral terminal). D-hairs are Aδ low-threshold afferents that form lanceolate terminal endings around hair follicles (Li et al., 2011), arise from medium-sized neurons, and show high expression of Kv1.1 (Shin et al., 2003). Although thought to be present only in hairy skin, we do find D-hair afferents also in the glabrous skin of plantar hindpaw. D-hairs are the most mechanically sensitive of all primary afferents (Lewin et al., 1992). In the *Cntnap2*$^{-/-}$ mouse, they show increased firing frequency both in the ramp and hold phases of the mechanical stimulus. Thus,

adaption that is normally very prominent in these afferents was impaired. In the "naive state," it was proposed that D-hairs are involved in priming central mechanisms prior to stimulus detection from other mechanoreceptors (Lechner and Lewin, 2013). Recent direct evidence for this arises from experiments in which optogenetic activation of TrkB expressing afferents (many of which are D-hairs) results in lowering of mechanical pain thresholds (Peng et al., 2017). Furthermore, in the context of pathological pain, D-hairs can contribute to the increased pain-related behavior (Li et al., 2017; Ventéo et al., 2016).

Given the integration of sensory inputs at the level of the spinal dorsal horn, we undertook extracellular recordings of WDR neurons. There was an enhanced response in *Cntnap2*$^{-/-}$ mice, particularly to mechanical and noxious heat stimulation. These neurons also demonstrated a lower response threshold to A-fiber strength and C-fiber strength electrical stimulation. There was no significant difference in the "wind up" properties of these neurons; whereas the response to the initial sensory input was significantly greater suggesting that the locus of action could be pre- rather than a postsynaptic event. Consistent with this, we did not observe any change in capsaicin-evoked synaptic responses in recording from lamina II dorsal horn neurons in *Cntnap2*$^{-/-}$ mice. We subsequently investigated the mechanism by which CASPR2 can regulate primary afferent excitability.

CASPR2 is known to associate with the shaker-type VGKCs. In *Cntnap2*$^{-/-}$ mice, Kv1 channels no longer cluster at the JXP, and we found that treatment of mice with human CASPR2-Abs led to a reduction in the JXP expression of CASPR2 and Kv1.1. Using *Cntnap2*$^{-/-}$ mice, Poliak et al. (2003) reported that there was no change in the conduction velocity or the refractory period. In agreement with this, when using single-unit recordings, we also found no change in the conduction velocity of sensory afferents in *Cntnap2*$^{-/-}$ mice. The lack of effect of altered Kv1 channel distribution in axons is a likely consequence of the fact that in normal circumstances juxtaparanodal Kv1 channels are electrically insulated from the node of Ranvier by compact myelin and do not modulate conduction characteristics (Rasband et al., 1998). We therefore investigated the function of CASPR2 in other neuronal compartments: DRG terminals and soma in which we do find a cell-autonomous effect of CASPR2 on neuronal excitability.

In recordings from cultured DRG neurons, we found that a loss of the FL-CASPR2 isoform resulted in enhanced excitability particularly of medium-sized DRG cells. Reduced expression of Kv1.1 and 1.2 has been associated with increased DRG cell excitability and enhanced behavioral responses to both noxious mechanical and thermal stimuli (Chi and Nicol, 2007; Hao et al., 2013; Zhao et al., 2013). We found a large reduction in the DTX-sensitive outward current in neurons from *Cntnap2*$^{-/-}$ mice, indicating a reduction in the function of Kv1 channels. WT neurons become hyper-excitable after 5 days in culture co-incident with reduced CASPR2 expression. This hyper-excitability could be reversed in an isoform-specific manner: overexpression of FL-CASPR2, but not of the short isoform, suppressed this hyper-excitability in a Kv1 channel-dependent manner. Kv1.1 is known to be highly expressed by D-hair afferents (Shin et al., 2003) that may partly explain why this specific population of afferents showed such enhanced mechanosensitivity of their terminals in the absence of FL-CASPR2. We also observed enhanced excitability in small DRG neurons from *Cntnap2*$^{-/-}$ mice, although this was less marked than that seen in medium-sized cells. The relative expression of Kv1 α subunits differs in distinct DRG neuronal populations with higher expression of Kv1.1 and 1.2 in medium and large DRG neurons and higher expression of Kv1.4 in small cells (Rasband et al., 2001). To summarize, CASPR2 is not only important for the longitudinal movement of Kv1 channels to the JXP domain of myelinated axons (Poliak et al., 2003), but FL-CASPR2 dynamically regulates the excitability of small- and particularly medium-sized DRG neurons. It is required for the expression of Kv1 channels at the soma membrane and in its absence these neurons become hyper-excitable.

We observed deposition of human IgG in the DRG (but not in spinal cord), and we therefore investigated whether CASPR2-Abs were able to directly affect soma excitability. IgG from both patients bound DRG neurons and did indeed increase their excitability. Binding of IgG, which is divalent, causes target internalization, which is one of the major pathological mechanisms in neurological autoimmune conditions (Ludwig et al., 2017). We found a reduction in Kv1 channel surface expression on DRG neurons following plasma treatment, consistent with this proposed mechanism. These autoantibodies, therefore, contribute to clinical pain by directly targeting neuronal molecules that regulate cell excitability. Although both patient IgGs reduced rheobase of DRG neurons, only patient 1 IgG enhanced repetitive firing in response to a supra-threshold stimulus. Given that both antibodies reduce surface expression of Kv1 channels, this difference is not easy to explain; however, multiple epitopes within CASPR2 have been shown to be recognized by CASPR2 autoantibodies that could contribute to heterogeneity in their functional effects (Olsen et al., 2015).

Gene ablation of CASPR2 leads to enhanced pain-related behavior in response to noxious mechanical, thermal stimuli, or algogens. This occurs as a consequence of increased DRG soma excitability due to impaired Kv1 channel function, increased mechanosensitivity of D-hair terminals, and is also associated with hyper-excitability at a spinal level. This is a further example of an ASD-linked gene mutation causing altered sensory function due to altered transduction/transmission within DRG neurons. We show for the first time that passive transfer of human CASPR2-Abs to WT mice can enhance neuronal excitability in a cell-autonomous manner and produce a peripheral neuropathic pain state as a consequence of an acquired channelopathy. This demonstrates antibody pathogenicity and, given the potential reversibility of excitability changes, provides a rationale for the identification of CASPR2-Abs in chronic pain patients and the appropriate use of immunotherapy.

STAR★METHODS

Detailed methods are provided in the online version of this paper and include the following:

- KEY RESOURCES TABLE
- CONTACT FOR REAGENT AND RESOURCE SHARING
- EXPERIMENTAL MODEL AND SUBJECT DETAILS

- Mouse lines and animal care
- Cell culture
- METHOD DETAILS
  - IgG purification
  - Passive transfer
  - Behavioral studies
  - Histology
  - RNA isolation and cDNA synthesis
  - Quantitative Real Time PCR
  - Electroporation
  - Plasmid construction
  - Cell based assay
  - Treatment of DRG neurons with human plasma
  - Quantification of membrane Kv1.2
  - Electrophysiology
  - AAV injections for DRG GCaMP6 expression
  - *In vivo* calcium imaging of DRG
- QUANTIFICATION AND STATISTICAL ANALYSIS

SUPPLEMENTAL INFORMATION

Supplemental Information includes ten figures, eight tables, and one data file and can be found with this article online at https://doi.org/10.1016/j.neuron.2018.01.033.

ACKNOWLEDGMENTS

We thank Professor Rocco Liguori for providing plasma from patient 1. We also thank Professor Manzoor A. Bhat for the CASPR antibody and Professor J. Trimmer for the Kv1.1 antibody. The project was supported by a strategic award from the Wellcome Trust (102645/Z/13/Z) and a research grant from the MRC (MR/M02394X/1). D.L.B. is a Wellcome senior clinical scientist (202747/Z/16/Z). S.J.M., L.J.P., and T.T. are students in the OXION programme, also funded by the Wellcome Trust.

AUTHOR CONTRIBUTIONS

Conceptualization, D.L.B., A.V., and J.M.D.; Methodology, D.L.B., A.V., J.M.D., G.A.W., G.R.L., S.J.M., A.H.D., R.P., A.J.T., N.I., S.B.M., K.I.C., and D.A.M.; Investigation, J.M.D., G.A.W., G.R.L., S.J.M., J.W., J.K., R.P., N.I., K.I.C., D.T., D.A.M., M.P.G., E.C., J.S., L.J.P., M.G.-M., M.D.S., K.W., S.J.W., A.S., I.C., J.G., L.C., T.T., H.K., A.C.T., C.B., M.I.L., and A.J.C.; Resources, P.P., L.J., B.L., H.U.Z., H.W., and E.P.; Writing – Original Draft, D.L.B. and J.M.D.; Writing – Review & Editing, D.L.B., J.M.D., A.V., H.U.Z., S.B.M., A.J.T., and G.R.L.; Visualization, J.M.D., G.A.W., S.J.M., and T.T.; Supervision, D.L.B., A.V., and J.M.D.; Funding Acquisition, D.L.B. and J.M.D.

DECLARATION OF INTERESTS

The University of Oxford and A.V. hold a patent for CASPR2 and LGI1 antibodies that is licensed to Euroimmun AG, Germany. A.V. receives a proportion of royalties. A.V., D.L.B., J.M.D., and G.A.W. have filed a patent relating to gene therapy to suppress excess neuronal activity in pain and epilepsy (PCT/GB2017/052909).

Received: August 8, 2017
Revised: December 4, 2017
Accepted: January 12, 2018
Published: February 8, 2018

REFERENCES

Arcourt, A., Gorham, L., Dhandapani, R., Prato, V., Taberner, F.J., Wende, H., Gangadharan, V., Birchmeier, C., Heppenstall, P.A., and Lechner, S.G. (2017). Touch receptor-derived sensory information alleviates acute pain signaling and fine-tunes nociceptive reflex coordination. Neuron *93*, 179–193.

Calvo, M., Richards, N., Schmid, A.B., Barroso, A., Zhu, L., Ivulic, D., Zhu, N., Anwandter, P., Bhat, M.A., Court, F.A., et al. (2016). Altered potassium channel distribution and composition in myelinated axons suppresses hyperexcitability following injury. eLife *5*, e12661.

Carter, R.J., Morton, J., and Dunnett, S.B. (2001). Motor coordination and balance in rodents. Curr. Protoc. Neurosci. *Chapter 8*. Unit 8.12.

Cascio, C.J. (2010). Somatosensory processing in neurodevelopmental disorders. J. Neurodev. Disord. *2*, 62–69.

Chaplan, S.R., Bach, F.W., Pogrel, J.W., Chung, J.M., and Yaksh, T.L. (1994). Quantitative assessment of tactile allodynia in the rat paw. J. Neurosci. Methods *53*, 55–63.

Chen, N., Koopmans, F., Gordon, A., Paliukhovich, I., Klaassen, R.V., van der Schors, R.C., Peles, E., Verhage, M., Smit, A.B., and Li, K.W. (2015). Interaction proteomics of canonical Caspr2 (CNTNAP2) reveals the presence of two Caspr2 isoforms with overlapping interactomes. Biochim. Biophys. Acta *1854*, 827–833.

Cheng, L., Duan, B., Huang, T., Zhang, Y., Chen, Y., Britz, O., Garcia-Campmany, L., Ren, X., Vong, L., Lowell, B.B., et al. (2017). Identification of spinal circuits involved in touch-evoked dynamic mechanical pain. Nat. Neurosci. *20*, 804–814.

Chi, X.X., and Nicol, G.D. (2007). Manipulation of the potassium channel Kv1.1 and its effect on neuronal excitability in rat sensory neurons. J. Neurophysiol. *98*, 2683–2692.

Coutinho, E., Menassa, D.A., Jacobson, L., West, S.J., Domingos, J., Moloney, T.C., Lang, B., Harrison, P.J., Bennett, D.L.H., Bannerman, D., and Vincent, A. (2017). Persistent microglial activation and synaptic loss with behavioral abnormalities in mouse offspring exposed to CASPR2-antibodies in utero. Acta Neuropathol. *134*, 567–583.

Fischer, M., Carli, G., Raboisson, P., and Reeh, P. (2014). The interphase of the formalin test. Pain *155*, 511–521.

Gold, M.S., Shuster, M.J., and Levine, J.D. (1996). Characterization of six voltage-gated K+ currents in adult rat sensory neurons. J. Neurophysiol. *75*, 2629–2646.

Gordon, A., Salomon, D., Barak, N., Pen, Y., Tsoory, M., Kimchi, T., and Peles, E. (2016). Expression of Cntnap2 (Caspr2) in multiple levels of sensory systems. Mol. Cell. Neurosci. *70*, 42–53.

Han, Q., Kim, Y.H., Wang, X., Liu, D., Zhang, Z.-J., Bey, A.L., Lay, M., Chang, W., Berta, T., Zhang, Y., et al. (2016). SHANK3 deficiency impairs heat hyperalgesia and TRPV1 signaling in primary sensory neurons. Neuron *92*, 1279–1293.

Hao, J., Padilla, F., Dandonneau, M., Lavebratt, C., Lesage, F., Noël, J., and Delmas, P. (2013). Kv1.1 channels act as mechanical brake in the senses of touch and pain. Neuron *77*, 899–914.

Hargreaves, K., Dubner, R., Brown, F., Flores, C., and Joris, J. (1988). A new and sensitive method for measuring thermal nociception in cutaneous hyperalgesia. Pain *32*, 77–88.

Horresh, I., Poliak, S., Grant, S., Bredt, D., Rasband, M.N., and Peles, E. (2008). Multiple molecular interactions determine the clustering of Caspr2 and Kv1 channels in myelinated axons. J. Neurosci. *28*, 14213–14222.

Irani, S.R., and Vincent, A. (2016). Voltage-gated potassium channel-complex autoimmunity and associated clinical syndromes. Handb. Clin. Neurol. *133*, 185–197.

Irani, S.R., Alexander, S., Waters, P., Kleopa, K.A., Pettingill, P., Zuliani, L., Peles, E., Buckley, C., Lang, B., and Vincent, A. (2010). Antibodies to Kv1 potassium channel-complex proteins leucine-rich, glioma inactivated 1 protein and contactin-associated protein-2 in limbic encephalitis, Morvan's syndrome and acquired neuromyotonia. Brain *133*, 2734–2748.

Irani, S.R., Pettingill, P., Kleopa, K.A., Schiza, N., Waters, P., Mazia, C., Zuliani, L., Watanabe, O., Lang, B., Buckley, C., and Vincent, A. (2012). Morvan syndrome: clinical and serological observations in 29 cases. Ann. Neurol. *72*, 241–255.

Klein, C.J., Lennon, V.A., Aston, P.A., McKeon, A., and Pittock, S.J. (2012). Chronic pain as a manifestation of potassium channel-complex autoimmunity. Neurology *79*, 1136–1144.

Lancaster, E., Huijbers, M.G.M., Bar, V., Boronat, A., Wong, A., Martinez-Hernandez, E., Wilson, C., Jacobs, D., Lai, M., Walker, R.W., et al. (2011). Investigations of caspr2, an autoantigen of encephalitis and neuromyotonia. Ann. Neurol. *69*, 303–311.

Lechner, S.G., and Lewin, G.R. (2013). Hairy sensation. Physiology (Bethesda) *28*, 142–150.

Lewin, G.R., Ritter, A.M., and Mendell, L.M. (1992). On the role of nerve growth factor in the development of myelinated nociceptors. J. Neurosci. *12*, 1896–1905.

Li, L., Rutlin, M., Abraira, V.E., Cassidy, C., Kus, L., Gong, S., Jankowski, M.P., Luo, W., Heintz, N., Koerber, H.R., et al. (2011). The functional organization of cutaneous low-threshold mechanosensory neurons. Cell *147*, 1615–1627.

Li, Y., Tatsui, C.E., Rhines, L.D., North, R.Y., Harrison, D.S., Cassidy, R.M., Johansson, C.A., Kosturakis, A.K., Edwards, D.D., Zhang, H., and Dougherty, P.M. (2017). Dorsal root ganglion neurons become hyperexcitable and increase expression of voltage-gated T-type calcium channels (Cav3.2) in paclitaxel-induced peripheral neuropathy. Pain *158*, 417–429.

Liguori, R., Vincent, A., Clover, L., Avoni, P., Plazzi, G., Cortelli, P., Baruzzi, A., Carey, T., Gambetti, P., Lugaresi, E., and Montagna, P. (2001). Morvan's syndrome: peripheral and central nervous system and cardiac involvement with antibodies to voltage-gated potassium channels. Brain *124*, 2417–2426.

Ludwig, R.J., Vanhoorelbeke, K., Leypoldt, F., Kaya, Z., Bieber, K., McLachlan, S.M., Komorowski, L., Luo, J., Cabral-Marques, O., Hammers, C.M., et al. (2017). Mechanisms of autoantibody-induced pathology. Front. Immunol. *8*, 603.

Madrid, R., de la Peña, E., Donovan-Rodriguez, T., Belmonte, C., and Viana, F. (2009). Variable threshold of trigeminal cold-thermosensitive neurons is determined by a balance between TRPM8 and Kv1 potassium channels. J. Neurosci. *29*, 3120–3131.

Manso, C., Querol, L., Mekaouche, M., Illa, I., and Devaux, J.J. (2016). Contactin-1 IgG4 antibodies cause paranode dismantling and conduction defects. Brain *139*, 1700–1712.

McMahon, S.B., La Russa, F., and Bennett, D.L.H. (2015). Crosstalk between the nociceptive and immune systems in host defence and disease. Nat. Rev. Neurosci. *16*, 389–402.

Milenkovic, N., Zhao, W.-J., Walcher, J., Albert, T., Siemens, J., Lewin, G.R., and Poulet, J.F.A. (2014). A somatosensory circuit for cooling perception in mice. Nat. Neurosci. *17*, 1560–1566.

Olsen, A.L., Lai, Y., Dalmau, J., Scherer, S.S., and Lancaster, E. (2015). Caspr2 autoantibodies target multiple epitopes. Neurol. Neuroimmunol. Neuroinflamm. *2*, e127.

Orefice, L.L.L., Zimmerman, A.L.L., Chirila, A.M.M., Sleboda, S.J.J., Head, J.P.P., and Ginty, D.D.D. (2016). Peripheral mechanosensory neuron dysfunction underlies tactile and behavioral deficits in mouse models of ASDs. Cell *166*, 299–313.

Peñagarikano, O., Abrahams, B.S., Herman, E.I., Winden, K.D., Gdalyahu, A., Dong, H., Sonnenblick, L.I., Gruver, R., Almajano, J., Bragin, A., et al. (2011). Absence of CNTNAP2 leads to epilepsy, neuronal migration abnormalities, and core autism-related deficits. Cell *147*, 235–246.

Peng, C., Li, L., Zhang, M.-D., Bengtsson Gonzales, C., Parisien, M., Belfer, I., Usoskin, D., Abdo, H., Furlan, A., Häring, M., et al. (2017). miR-183 cluster scales mechanical pain sensitivity by regulating basal and neuropathic pain genes. Science *356*, 1168–1171.

Poliak, S., Salomon, D., Elhanany, H., Sabanay, H., Kiernan, B., Pevny, L., Stewart, C.L., Xu, X., Chiu, S.Y., Shrager, P., et al. (2003). Juxtaparanodal clustering of Shaker-like K+ channels in myelinated axons depends on Caspr2 and TAG-1. J. Cell Biol. *162*, 1149–1160.

Rasband, M.N., Trimmer, J.S., Schwarz, T.L., Levinson, S.R., Ellisman, M.H., Schachner, M., and Shrager, P. (1998). Potassium channel distribution, clustering, and function in remyelinating rat axons. J. Neurosci. *18*, 36–47.

Rasband, M.N., Park, E.W., Vanderah, T.W., Lai, J., Porreca, F., and Trimmer, J.S. (2001). Distinct potassium channels on pain-sensing neurons. Proc. Natl. Acad. Sci. USA *98*, 13373–13378.

Rodenas-Cuadrado, P., Ho, J., and Vernes, S.C. (2014). Shining a light on CNTNAP2: complex functions to complex disorders. Eur. J. Hum. Genet. *22*, 171–178.

Ruts, L., van Doorn, P.A., Lombardi, R., Haasdijk, E.D., Penza, P., Tulen, J.H.M., Hempel, R.J., van den Meiracker, A.H., and Lauria, G. (2012). Unmyelinated and myelinated skin nerve damage in Guillain-Barré syndrome: correlation with pain and recovery. Pain *153*, 399–409.

Shin, J.-B., Martinez-Salgado, C., Heppenstall, P.A., and Lewin, G.R. (2003). A T-type calcium channel required for normal function of a mammalian mechanoreceptor. Nat. Neurosci. *6*, 724–730.

Strauss, K.A., Puffenberger, E.G., Huentelman, M.J., Gottlieb, S., Dobrin, S.E., Parod, J.M., Stephan, D.A., and Morton, D.H. (2006). Recessive symptomatic focal epilepsy and mutant contactin-associated protein-like 2. N. Engl. J. Med. *354*, 1370–1377.

Tékus, V., Hajna, Z., Borbély, É., Markovics, A., Bagoly, T., Szolcsányi, J., Thompson, V., Kemény, Á., Helyes, Z., and Goebel, A. (2014). A CRPS-IgG-transfer-trauma model reproducing inflammatory and positive sensory signs associated with complex regional pain syndrome. Pain *155*, 299–308.

Tsujino, H., Kondo, E., Fukuoka, T., Dai, Y., Tokunaga, A., Miki, K., Yonenobu, K., Ochi, T., and Noguchi, K. (2000). Activating transcription factor 3 (ATF3) induction by axotomy in sensory and motoneurons: A novel neuronal marker of nerve injury. Mol. Cell. Neurosci. *15*, 170–182.

Varea, O., Martin-de-Saavedra, M.D., Kopeikina, K.J., Schürmann, B., Fleming, H.J., Fawcett-Patel, J.M., Bach, A., Jang, S., Peles, E., Kim, E., and Penzes, P. (2015). Synaptic abnormalities and cytoplasmic glutamate receptor aggregates in contactin associated protein-like 2/Caspr2 knockout neurons. Proc. Natl. Acad. Sci. USA *112*, 6176–6181.

Ventéo, S., Laffray, S., Wetzel, C., Rivat, C., Scamps, F., Méchaly, I., Bauchet, L., Raoul, C., Bourinet, E., Lewin, G.R., et al. (2016). Fxyd2 regulates Aδ- and C-fiber mechanosensitivity and is required for the maintenance of neuropathic pain. Sci. Rep. *6*, 36407.

Wendykier, P., and Nagy, J.G. (2010). Parallel Colt. ACM Trans. Math. Softw. *37*, 1–22.

Wigerblad, G., Bas, D.B., Fernades-Cerqueira, C., Krishnamurthy, A., Nandakumar, K.S., Rogoz, K., Kato, J., Sandor, K., Su, J., Jimenez-Andrade, J.M., et al. (2016). Autoantibodies to citrullinated proteins induce joint pain independent of inflammation via a chemokine-dependent mechanism. Ann. Rheum. Dis. *75*, 730–738.

Zeilhofer, H.U., Studler, B., Arabadzisz, D., Schweizer, C., Ahmadi, S., Layh, B., Bösl, M.R., and Fritschy, J.-M. (2005). Glycinergic neurons expressing enhanced green fluorescent protein in bacterial artificial chromosome transgenic mice. J. Comp. Neurol. *482*, 123–141.

Zhao, X., Tang, Z., Zhang, H., Atianjoh, F.E., Zhao, J.-Y., Liang, L., Wang, W., Guan, X., Kao, S.-C., Tiwari, V., et al. (2013). A long noncoding RNA contributes to neuropathic pain by silencing Kcna2 in primary afferent neurons. Nat. Neurosci. *16*, 1024–1031.

STAR★METHODS

KEY RESOURCES TABLE

REAGENT or RESOURCE	SOURCE	IDENTIFIER
Antibodies		
Rabbit anti-AFT3 (C-19)	Santa Cruz	Cat# sc-188, RRID: AB_2258513
Sheep anti-CGRP	Enzo Life Sciences	Cat# BML-CA1137, RRID: AB_2050885
Rabbit anti-CGRP	Peninsula Laboratories	Cat# T-4032, RRID: AB_2313775
Isolectin B_4 (IB4), conjugated to biotin	Sigma-Aldrich	Cat# L2140, RRID: AB_2313663
Rabbit anti-PGP9.5	UltraClone	Discontinued
Mouse anti-NF200	Sigma-Aldrich	Cat# N0142, RRID: AB_477257
Chicken anti-NF200	Abcam	Cat# ab4680, RRID: AB_304560
Rabbit anti-CASPR2	Abcam	Cat# ab33994, RRID: AB_2083506
Guinea pig anti-CASPR	Gift from Bhat MA	N/A
Rabbit anti-IBA1	Wako	Cat# 019-19741, RRID: AB_839504
Rabbit anti-Kv1.1	Gift from Trimmer J	N/A
Mouse anti-Kv1.2	UC Davis/NIH NeuroMab Facility	Cat#75-008, RRID: AB_2296313
Mouse anti-Tubulin III, beta	Sigma-Aldrich	Cat#T8578 RRID: AB_1841228
Rabbit anti-PAX2	Thermo Fisher Scientific	Cat# 71-6000, RRID: AB_2533990
Rabbit anti-NeuN	Abcam	Cat# ab177487, RRID: AB_2532109
Chicken anti-NeuN	Millipore	Cat# ABN91, RRID: AB_11212808
Goat anti-TrkB	R and D Systems	Cat# AF1494, RRID: AB_2155264
Sheep anti-Tyrosine Hydroxylase	Millipore	Cat# AB1542, RRID: AB_90755
Rabbit anti-Gephyrin	Frontier Institute	Cat# Gephyrin-Rb-Af1330, RRID: AB_2571705
Goat anti-VGAT	Frontier Institute	Cat# VGAT-Go-Af620, RRID: AB_2571623
Rabbit anti-c-FOS	Santa Cruz	Cat# sc-52, RRID: AB_2106783
Rabbit anti-CD3	Abcam	Cat# ab16669, RRID: AB_443425
Rat anti-CD68	Bio-Rad	Cat# MCA1957, RRID: AB_322219
Rat anti-Gr-1(Ly-6G)	R and D Systems	Cat# MAB1037, RRID: AB_2232806
Rabbit anti-GFAP	Dako	Cat# Z0334, RRID: AB_10013382
Chicken anti-GFP	Abcam	Cat# ab13970, RRID: AB_300798
Donkey anti-rabbit IgG Alexa 488	Thermo Fisher Scientific	Cat# A-21206, RRID:AB_2535792
Donkey anti-rabbit IgG Cy3	Jackson ImmunoResearch Labs	Cat# 711-166-152, RRID: AB_2313568
Goat anti-rabbit IgG Pacific blue	Thermo Fisher Scientific	Cat# P-10994, RRID: AB_2539814
Donkey anti-rabbit IgG Alexa 546	Thermo Fisher Scientific	Cat# A10040, RRID: AB_2534016
Donkey Anti-sheep IgG Cy3	Jackson ImmunoResearch Labs	Cat# 713-166-147, RRID: AB_2340729
Streptavidin Pacific blue	Thermo Fisher Scientific	Cat# S11222
Streptavidin Alexa 405	Thermo Fisher Scientific	Cat# S32351
Donkey anti-mouse IgG Alexa 488	Thermo Fisher Scientific	Cat# A-21202, RRID: AB_141607
Goat anti-mouse IgG Pacific blue	Thermo Fisher Scientific	Cat# P31582, RRID: AB_10374586
Goat anti-guinea pig IgG Alexa 568	Thermo Fisher Scientific	Cat# A-11075, RRID: AB_2534119
Goat anti-human IgG Alexa 488	Thermo Fisher Scientific	Cat# A-11013, RRID: AB_2534080
Goat anti-human IgG Alexa 546	Thermo Fisher Scientific	Cat# A-21089, RRID: AB_2535745
NeuroTrace 530/615 Red Fluorescent Nissl Stain	Thermo Fisher Scientific	Cat# N21482, RRID: AB_2620170
Donkey anti-sheep IgG Alexa 488	Thermo Fisher Scientific	Cat# A-11015, RRID: AB_2534082
Goat anti-rat IgG Alexa 488	Thermo Fisher Scientific	Cat# A-11006, RRID: AB_2534074
Goat anti-rat IgG Alexa 546	Thermo Fisher Scientific	Cat# A11081, RRID: AB_10563603

(*Continued on next page*)

Continued		
REAGENT or RESOURCE	SOURCE	IDENTIFIER
Goat anti-chicken Alexa 488	Thermo Fisher Scientific	Cat# A-11039, RRID: AB_2534096
Goat anti-chicken Alexa 546	Thermo Fisher Scientific	Cat# A-11040, RRID: AB_2534097
Donkey anti-goat Alexa 546	Thermo Fisher Scientific	Cat# A-11056, RRID: AB_2534103
Donkey Anti-goat Alexa 488	Thermo Fisher Scientific	Cat# A-11055, RRID: AB_142672
Bacterial and Virus Strains		
AAV9.CAG.GCaMP6s.WPRE.SV40	UPENN Vector Core	Cat# AV-1-PV2833
Chemicals, Peptides, and Recombinant Proteins		
α-Dendrotoxin	Alomone labs	Cat#D-350
Capsaicin	Sigma-Aldrich	Cat#M2028
Formaldehyde solution	Sigma-Aldrich	Cat#252549
Acetone	WVR Chemicals	Cat#20066.321
ethyl chloride	Miller Medical Supplies	discontinued
Critical Commercial Assays		
RNAscope 2.5 HD Reagent Kit-RED	Advanced Cell Diagnostics	Cat# 322350
Experimental Models: Organisms/Strains		
Mouse C57BL/6	Biomedical services Oxford University	JAX Mice Stock Number 000664
Mouse *Cntnap2*$^{-/-}$	Peles lab	JAX Mice Stock Number: 017482
Mouse GlyT2-EGFP	Zeilhofer Lab	N/A
Oligonucleotides		
For qPCR primers see Table S8	This paper	N/A
RNAscope Probe- Mm-Cntnap2	Advanced Cell Diagnostics	Cat# 449381
Recombinant DNA		
FL-Human-CASPR2-EGFP	Irani et al., 2010	N/A
SH-Human-CASPR2-EGFP	This paper	N/A
pAcEGFP-C1	Clontech	Cat# 632470
Software and Algorithms		
ImageJ/Fiji	NIH	https://imagej.nih.gov/ij/index.html, https://fiji.sc/
Clampfit 10	Molecular Devices	http://mdc.custhelp.com/app/answers/detail/a_id/18779/~/axon%E2%84%A2-pclamp%E2%84%A2-10-electrophysiology-data-acquisition-%26-analysis-software
LabChart v7.3	ADInstruments	https://www.adinstruments.com/support/software/archive
Spike2	Cambridge electronic design	http://ced.co.uk/
Mini Analysis software	Synaptosoft	http://www.synaptosoft.com/MiniAnalysis/
Prism 7.0	GraphPad Software	https://www.graphpad.com/
SigmaPlot 13	Systat sotware	http://www.sigmaplot.co.uk/

CONTACT FOR REAGENT AND RESOURCE SHARING

Further information and requests for resources should be directed to and will be fulfilled by the Lead Contact, David Bennett (david.bennett@ndcn.ox.ac.uk).

EXPERIMENTAL MODEL AND SUBJECT DETAILS

Mouse lines and animal care

All procedures were carried out in accordance with UK home office regulations and in line with the Animals Scientific Procedures Act 1986 at a licensed facility within the University of Oxford, following institutional review board approval. Animals were group housed in

IVC cages in temperature and humidity controlled rooms where food and water was available *ad libitum*, with a 12 hour light dark cycle. The welfare of all animals was continually assessed throughout all procedures.

Cntnap2$^{-/-}$ mice, were generated by E Peles and have previously been described (Poliak et al., 2003). These mice were maintained on a C57BL/6J background. Heterozygous mice were bred together to obtain both *Cntnap2*$^{-/-}$, *Cntnap2*$^{+/-}$ and *Cntnap2*$^{+/+}$ littermates. Genotyping of offspring was performed by PCR of genomic DNA using primers to detect either the wild-type (5′-TGC TGCTGCCAGCCCAGAACTGG-3′ to 5′-TCAGAGTTGATACCCGAGCGCC-3′) or mutated allele (5′-AGCAGCCGATTGTCTGT TGT-3′ to 5′-CTCACCCAATCTCACAAACAAG-3′) of *Cntnap2*. Glycine transporter 2-eGFP reporter mice have been previously described (Zeilhofer et al., 2005). Both male and female adult *Cntnap2*$^{-/-}$/*Cntnap2*$^{+/-}$/*Cntnap2*$^{+/+}$ mice were used for experimental studies. For human IgG studies male C57BL/6J mice were used. For all behavior and tissue analysis studies, mice were aged between 8-16 weeks. For cell culture mice were aged between 4-8 weeks. This study conforms to the ARRIVE guidelines.

Cell culture

Adult male and female mice of 4-8 weeks of age were sacrificed in a CO_2 chamber. The spinal column was rapidly removed and bisected to reveal the DRG. DRG were taken from all levels and placed directly into Hanks' Balanced Salt solution (HBSS without Ca^{2+} and Mg^{2+}, Invitrogen). DRG were then subjected to enzymatic digestion using collagenase II (12mg/ml, Worthington) and dispase II (14mg/ml, Roche) diluted in HBSS for 1.5 hours at 37°C. DRGs were then washed in HBSS and mechanically dissociated using fire polished pipettes. Dissociated cells were suspended in culture medium (Neurobasal medium, B27, glutamax, GIBCO) supplemented with mouse NGF (50ng/μl, Peprotech) and GDNF (10ng/μl, Peprotech), and plated on to laminin/poly-D Lysine coated coverslips before being incubated at 37°C.

Human embryonic kidney 293 (HEK293) cells were cultured in Dulbeccos's modified Eagles's medium with 10% fetal calf serum (TCS Cellworks Ltd) in 6 well plates at 37°C. Penicillin G and streptomycin were added to the culture medium to prevent infection.

METHOD DETAILS

IgG purification

The IgG fraction was purified from the plasma of both CASPR2-Ab patients and the healthy control using protein G Sepharose beads (Sigma). The plasma was diluted 1:4 with Hartmann's solution and passed through a column containing the Sepharose beads at a flow rate of ~0.5ml/minute. Once the diluted plasma had passed through the column, additional Hartmann's solution was used to wash the beads to ensure no non-specific proteins were present. The IgG was then eluted with 0.1M glycine solution (pH 2.3) and immediately neutralised with 1M Tris (pH 8). Coomassie Plus assay kit (Pierce) was used to determine the protein content of the eluted fractions. Fractions with high protein content were dialysed against Hartmann's solution, concentrated using polyethylene glycol and filter sterilized. IgG concentration was determined by spectrometry. For Patient 1 the purified IgG was concentrated to 15mg/ml, for Patient 2 to 11mg/ml and for healthy control to 15mg/ml and 12.5mg/ml, respectively.

Passive transfer

C57BL/6 male mice (8-12 weeks of age) received daily intraperitoneal injections of sterile purified human IgG for either 2 or 3 weeks. All injections were given between 5-6pm and were therefore carried out subsequent to any behavioral testing that had occurred the same day. For experiments involving patient 1, mice received 6mg of purified IgG daily of either patient or healthy control IgG. For experiments involving patient 2, mice received 10mg of purified IgG daily of either patient or control IgG. Mice were closely observed throughout the dosing regime and their weight monitored. At termination of the injections, blood was taken for serology and tissue taken for histology as described below.

Behavioral studies

The same designated room was used for all behavioral studies and testing was performed at a consistent time of day. Mice were acclimatised to the testing equipment and baseline values were obtained by averaging data from 2-3 sessions. For human IgG studies, baseline values were used to allocate mice into matching groups prior to treatment. Sample sizes were chosen based on a power calculation using historical data relating to mechanical and thermal threshold responses (α error of 0.05 and a power of 80%). It was calculated that groups of 8 would be needed, using the assumption that an effect size of 25% would be biological meaningful. In total 45 *Cntnap2*$^{-/-}$ 7 *Cntnap2*$^{+/-}$ mice and 48 *Cntnap2*$^{+/+}$ littermates were used in behavioral studies (56 males and 44 females). Mechanical hypersensitivity was replicated in a second independent cohort of mice. Male C57BL/6 mice were used for all human IgG studies. In total 17 mice were injected with healthy control IgG, 8 with patient 1 IgG and 9 with patient 2 IgG. For all behavior experiments mice from different experimental groups were assessed at the same time. Mechanical sensitivity was assessed by placing mice in a Perspex box situated on top of a wire mesh. Calibrated Von Frey hairs (Ugo Basile) were then applied to the plantar surface of the hind paw and a reflex withdrawal response was used to calculate the 50% withdrawal threshold (Chaplan et al., 1994). Dynamic mechanical allodynia was assessed using a recently published protocol (Cheng et al., 2017). A small paintbrush (5/0, The Art Shop) was modified by trimming the tip to make it blunt. This was used to gently stroke the plantar surface of the paw. A scoring system was used as follows to determine a dynamic allodynia score, (0) (a none painful response) lifting of the paw for less than 1 s, (1) sustained lifting of the paw or a single flinch, (2) lateral paw lift above the level of the body or a startle like jump and (3)

multiple flinching responses or licking of the affected paw. The response to a clear noxious mechanical stimulus was also assessed using the pin prick test as previously described (Arcourt et al., 2017). A dissecting pin was attached to a 1 g Von Frey filament and applied to the plantar surface of the hind paw to elicit a rapid withdrawal reflex. The latency to withdraw was recorded using an iPhone 6 (Apple) at 240fps (4.16ms per frame) and analyzed using the video editing program Avidemux. Thermal sensitivity was initially assessed using the Hargreave's method (Hargreaves et al., 1988). Here using the Hargreave's apparatus (Ugo Baslie) a radiant heat source was applied to the plantar surface of the hind paw and the latency to withdrawal was used to determine heat sensitivity threshold. Response to a suprathreshold heat stimulus was measured using the hot plate (Ugo Basile) assay. A metallic plate was set so that the surface temperature was at either 50°C or 53°C. Mice were then placed onto the plate and the latency until a response, in this case shaking, licking or biting of the paw, was measured. To assess cold sensitivity a thermal preference paradigm was used. The thermal preference equipment (Ugo Basile) consisted of two plates with a small connecting bridge. The plates were set at either 16°C or room temperature. Mice were then assessed over a 10 minute period and the percentage of time spent at 16°C was calculated. Pilot studies had identified this temperature scenario as optimal (data not shown). For assessment of capsaicin sensitivity, mice received an intraplantar injection of 1.5μg of capsaicin (Sigma) diluted in sterile saline with 1% ethanol and 0.5% tween in a volume of 10 μl. Mice were placed in a Perspex cylinder and the duration of pain-related behavior, biting/licking/paw lifting, was recorded over a 5 minute period. For the formalin test, mice received an intraplantar injection of 20 μl of 5% formalin diluted from formaldehyde solution (Sigma) in sterile saline. Again, mice were placed in a Perspex cylinder and the duration of pain-related behavior, biting/licking/paw lifting, was recorded over a 60 minute period. The behavioral response is biphasic and therefore further comparisons were made by pooling data in the first (0-15mins) and second (15-60mins) phases. Motor behavior was assessed using a RotaRod. Mice were placed on a rotating rod which was either accelerating from 4-32rpm or at a constant speed (28 rpm). The latency until the mouse could no longer stay on the rod was recorded. For the open field test, a black box displaying a grid system on the floor was used. Mice were placed in this box for 3 minutes and the number of boxes the mouse entered during this period was recorded. Proprioception was also assessed using the beam walk test as previously described (Carter et al., 2001). This test used a wooden beam of about 1 m in length which was elevated from the bench surface. Mice were filmed as they moved along the beam and the percentage of correct steps was calculated by counting the number of missed steps and comparing to the total number of steps taken for each run. All behavior studies were carried out with the experimenter blind to treatment group or genotype. No animals were excluded from the behavioral analysis.

Histology

Tissue preparation

For immunohistochemistry and *in situ* hybridization studies, mice were overdosed with pentobarbital and transcardially perfused, initially with sterile saline and then 20mls of 4% paraformaldehyde (PFA, 0.1M Phosphate buffer (PB)). Once dissected the glabrous skin and sciatic nerves were post-fixed in 4% PFA for 0.5 hours at RT, DRG for 2 hours and spinal cord overnight at 4°C. All tissue was dehydrated for cyroprotection in 30% sucrose (0.1M PB) at 4°C for at least 24 hours. Tissue was then embedded in optimal cutting temperature (OCT) medium (Tissue-Tek) and stored at −80. Tissue was then placed into a solution containing only 30% sucrose before being embedded in OCT. Tissue was sectioned onto Superfrost plus slides (VWR) using a cryostat. For skin, gelatine treated slides were used. Sciatic nerve and DRG sections were cut at 10 μm, skin at 14 μm and spinal cord at 30 μm. The slides were then stored at −80.

Immunohistochemistry (IHC)

Tissue sections were washed once in PBS and then blocked for 1 hour before being incubated overnight at RT with primary antibody diluted in PBS triton-X (0.3%) (Table S7). For nodal staining in the sciatic nerve, tissue sections were subjected to a mild antigen retrieval protocol. This involved placing slides containing tissue sections into an EDTA buffer (10mM tris Base, 1mM EDTA, 0.05% Tween, pH9.0) at 60°C for 4-6 hours before blocking and primary Ab incubation. Primary Ab was washed off in PBS triton-X and tissue was then incubated with secondary antibodies (Table S7) at RT for 3-4 hours. Cultured DRG neurons were fixed in 4% PFA for 10 minutes, washed in PBS, and then incubated with primary Ab diluted in blocking solution (Goat or donkey serum, PBS-Tx 0.3%) overnight at 4°C. Coverslips were washed in PBS and incubated with secondary Ab for 2 hours at RT. Immunostaining was visualized using a confocal microscope (Zeiss) and images acquired using the Zen black software.

Skin whole-mount IHC

Briefly, the hind paw hairy and glabrous skin of mice was dissected and post-fixed in 4% PFA for 2 hours. Following this, samples were washed in PBS and then bleached in Dent's Bleach (33% H_2O_2, 13.3% DMSO, 53.3% methanol), for 24 hours at 4°C with rotation. Samples were blocked and incubated with antibodies in the same manner as mentioned above but with longer incubation times (primary antibodies 4 days, secondary antibodies, 2 days). Following antibody incubation the tissue was cleared using 50% 80% and 100% tetrahydrofuran(THF)/ddH_2O for 30 minutes (100% for 1 hour) and then in 10% 25% 50% and 97% 2-2 Thiodiethanol (TDE)/PBS/ddH_2O, for 2 hours at RT. Samples were stored in 97%TDE/PBS and embedded onto microscope slides using 460μm thick adhesive foam spacers. Immunostaining was visualized using a confocal microscope (Zeiss) and images acquired using the Zen black software.

In situ *hybridization (ISH)*

Once cut, sections were air-dried in the cryostat for 0.5 hours and then stored in the −80°C. ISH was carried out using the RNAScope 2.5 RED chromogenic assay kit and by following the manufacturer's instructions (Advanced Cell Diagnostics). Briefly, tissue sections

were removed from the −80, allowed to equilibrate to RT and re-hydrated in PBS. Pre-treatment required a hydrogen peroxide step at RT; followed by a protease treatment in a hybridization oven at 40°C. Slides were then incubated with the target or control probes at 40°C for 2 hours. For CASPR2 mRNA the probes were designed to target position 3708-5086 of NM_001004357.2. Following probe incubation, slides were subjected to 6 rounds of amplification and the probe signal was developed via a reaction with fast red. To combine with IHC, tissue sections were then washed with PBS-Tx (0.3%) and subjected to the standard IHC protocol.

Electron microscopy and analysis

Animals which were either treated with purified IgG from either Patient 1, Patient 2 or healthy control were terminally anesthetized using sodium pentobarbital (Euthatal; 80 mg/kg, i.p.) and transcardially perfused with 0.9% saline followed and 4% PFA in 0.1 M PB. A section of the sural nerve was taken and post-fixed in 3% glutaraldehyde and 4% PFA in 0.1 M PB) at 4°C overnight, washed in 0.1 M PB, osmicated, dehydrated, and embedded in epoxy resin (TAAB Embedding Materials). Ultrathin sections (90 nm) were taken using a Diatome diamond knife on the Leica UC7 ultramicrotome and mounted onto 200 mesh Cu grids. Sections were post-stained with Reynold's lead citrate for 5 minutes, washed with degassed water and dried. Samples were transferred to a FEI Tecnai 12 transmission electron microscope and imaged at 120kV. Images were acquired using a Gatan OneView CMOS camera with Digital Micrograph 3.0 software. Full montages of grid squares were taken (~25 pictures per mesh) and randomly chosen images from a given grid square were analyzed. The total number of axons, total number of myelinated axons, Schwann cell-myelinated axons, were counted from these montages of grid squares and normalized to the total area. To calculate G-ratios, axon diameters and non-myelinated axons with a diameter > 1 μm, individual pictures at the same magnification were randomly chosen per animal; analysis was performed on all of the axons within each picture and axon diameter and G-ratio (axon diameter/fiber diameter) were calculated using AxioVision LE Rel. 4.2 Software. The examiner was blind to the treatment group.

Image Analysis

Analysis of the signal intensity for *in situ* hybridization studies on DRG was calculated using ImageJ software. In a single image of a section of either L4 or 5 DRG, neurons with a nucleus were circled and the percentage coverage of red signal for that cell profile area measurement was calculated. By eye the cell was then subpopulation defined by using primary antibodies against NF200, IB4, CGRP or TH and the appropriate secondary antibody (Table S7). For each marker at least 3 sections were imaged per animal. On each image a background reading was taken from an area of tissue not containing neuronal cell bodies. An average of this was calculated for each animal and any cell that had percentage coverage of greater than 2 standard deviations from the background mean were defined as being positive for CASPR2 mRNA. For spinal cord sections, CASPR2 mRNA positive cells were defined as those containing 4 or more red dots and the percentage of Pax2 expressing cells positive for CASPR2 mRNA was calculated. For analysis of neuronal activity following formalin (5%) injection, *Cntnap2*$^{-/-}$ and control mice were perfused two hours post injection and tissue was incubated with a primary antibody against c-*fos* (Table S7). NeuN was used to mark neuronal cell bodies (Table S7). The average number of c-*fos* positive neurons in the dorsal horn was calculated from 3-5 sections per animal for both ipsilateral and contralateral sides. For the quantification of DRG neuron subpopulations in *Cntnap2*$^{-/-}$ mice, 3 sections of the L4 DRG were imaged and used for each animal with > 100 cells counted for each subpopulation. The number of cells for each marker is shown as a percentage of the sum of NF200, IB4 and CGRP positive cells. For intraepidermal nerve fiber density counts, 3-4 sections of glabrous skin taken lateral to the proximal end of the most proximal walking pad were used and imaged for each animal. Nerve fibers were identified using a primary antibody against PGP9.5 (Table S7). The numbers of free nerve endings in the epidermis were calculated using the EFNS guidelines which meant that only fibers that penetrated into the epidermis from the dermal layer were counted. For analysis of Pax2 positive inhibitory interneurons in *Cntnap2*$^{-/-}$ mice, tissue was incubated in primary antibody against Pax2 (Table S7) and 3 sections from the L4 segment of the mouse spinal cord were scanned. The number of Pax2 positive neurons in a 10μm depth across the dorsal horn was calculated and shown as the percentage of total neurons (NeuN positive). For human IgG binding studies sciatic nerve, DRG and spinal cord sections were analyzed. Sciatic nerve sections were incubated with a primary antibody against the paranodal marker CASPR, DRG sections with a primary antibody against NeuN to mark neuronal cell bodies and spinal cord sections with Nissl to mark neurons and DAPI for distinguishing cell nuclei (Table S7). Sections were then incubated in the appropriate secondary antibodies including anti-human IgG to visualize CASPR2-Ab binding *in vivo* (Table S7). Permeabilised (0.3% Triton-X) naive wild-type sciatic nerve sections were incubated with purified patient IgG and an antibody against CASPR (paranode) to show the ability of CASPR2-Abs to bind in the sciatic nerve when it has access to the JXP. For analysis of immune cell infiltration in mice treated with purified human IgG from either healthy control or CASPR2-Ab positive patients, primary antibodies against IBA1, Ly6G and CD3 were used to mark, macrophages, neutrophils and T lymphocytes, respectively (Table S7). All of these markers were used to analyze DRG and spinal cord tissue and a primary antibody against NeuN was used to mark neuronal cell bodies. IBA1 was also used in sciatic nerve sections. Between 3 and 7 sections were imaged for each animal and the average number of cells per image calculated. Astrocyte activity was measured using a primary antibody against GFAP (Table S7). The intensity of the GFAP signal in the superficial dorsal horn was measured using the analysis tool in ImageJ and an average taken from 3-5 sections were animal. L4 DRGs were also used for ATF3 analysis. Tissue was incubated with a primary antibody against AFT3 (Table S7) and 3 sections per animal were imaged and the number of ATF3 positive cells was calculated as a percentage of total neurons using a nissl counterstain (Table S7). For nodal counts 3 sciatic nerve sections were imaged for each animal. For the calculation of the number of total nodes, an antibody against CASPR (Table S7) was used to mark the paranode. The number of CASPR positive nodes were counted and shown as the number of nodes per 1000μm^2 of sciatic nerve. Antibodies against CASPR2 and Kv1.1 (Table S7) were used to mark nodes for these proteins and their amount was calculated as a percentage of total CASPR positive nodes. For analysis of the area of CASPR2 and Kv channel

immunostaining in the JXP thresholding of the images was performed using the default setting in the ImageJ software. This helped to remove background noise and the area of immunostaining was then measured for the JXP region of each node, where a node was defined by being CASPR positive. For tissue analysis composite images are shown. All quantification was performed with the experimenter blind to treatment groups.

Brain tissue processing and microglia analysis

Brains were removed and post-fixed for 24 hours at room temperature before transferring to 40% sucrose in PBS-azide (0.01%). Using a freezing stage sledge microtome, free-floating coronal sections were collected in 15 series at a thickness of 50 μm. Similar to that recently described (Coutinho et al., 2017), reactive microglial cells were identified by immunofluorescence using rat anti-CD68 and rabbit anti-IBA1 primary antibodies (Table S7) on free-floating sections. Sections were fixed with 4% formaldehyde, washed with PBS then blocked with 10% normal goat serum in PBS-Triton X-100 (0.3%) for an hour then incubated for 48 hours with primary antibodies at 4°C. The sections were washed the next day with PBS-Triton X-100 (0.3%) then incubated for three hours at room temperature with goat anti-rat (488) and goat anti-rabbit (568) Alexa Fluor secondary antibodies (Table S7) and kept in the dark. Sections were subsequently washed in PBS-T (0.3%), final wash was in PBS then sections were mounted on slides after a brief TNS wash (pH = 7.4) and counterstained with DAPI mounting medium from Vector Laboratories (UK), left to dry for an hour then sealed and stored protected from the light at −20°C until ready for confocal imaging.

Quantification of activated microglia (defined as CD68/IBA1 positive cells) was performed in layers I, II-IV and V-VI in the primary somatosensory cortex (Bregma −1.46 mm). The area was 110.87 μm x 110.87 μm in (x,y) and the z-step/interval was 2 μm and microglial cells were counted within a 50 μm depth. 8 z stacks for layer I and 6 stacks for remaining layers were imaged. An average density was obtained (cells/mm^3) in both hemispheres. At least 150 cells per layer per condition were counted. Microglial morphology was assessed in 20 confocal z stacks/case at 40X magnification (26 μm thickness with imaging every 2 μm) detecting fluorescence in IBA1 expressing cells in layers of the somatosensory cortex. Minimum resolution of 512x512 was used and with a line averaging of 4 to allow for a detailed assessment of microglial cells in each group. A macro (see below) from Fiji (image analysis software) was used to obtain in each stack a clear representation of the cell body and processes of 100 microglial cells per group. Soma size (μm^2) and number of processes per cell was recorded manually in Fiji. Microglia morphology macro: run(“Z Project...,” “projection=[Max Intensity]”); run(“Duplicate...,” “duplicate channels=3”); setAutoThreshold(“Huang”); setOption(“BlackBackground,” false); run(“Convert to Mask”); run(“Despeckle”); run(“Invert”); run(“Analyze Particles...,” “size=500-Infinity pixel show=Masks clear”);

IHC of synaptic proteins

Rabbit anti-Gephyrin antibody and goat anti-VGAT antibody were used to identify the post-synaptic density of symmetric synapses, and IB4 conjugated to biotin was used to label non-peptidergic primary afferents (Table S7). 30 μm sections were mounted onto SuperFrost slides, air-dried for 48 hours, rehydrated in PBS for 15 min then placed in a solution of 50% ethanol: PBS-Azide (0.01%) for 30 min at room temperature. Sections were treated in citrate-EDTA buffer (10 mM citric acid, 2 mM EDTA, 0.05% Tween-20 at pH = 6.2) to a heat-mediated antigen retrieval step in citrate buffer for 10 min. Proteinase K treatment (4 μg/mL) for 10 min followed by a 10 minute pepsin treatment (0.1 mg/mL) at 37°C in 0.2N HCl solution was performed. Sections were incubated with primary antibodies diluted in PBS containing 0.3% Triton X-100 & 0.1% azide (PBSTxAz) for three days at room temperature, washed, then incubated overnight with anti-rabbit and anti-goat secondary antibodies conjugated to Alexa Fluor 488 and Alexa Fluor 546 respectively, and streptavidin conjugated to pacific blue in PBSTxAz (Table S7). Sections were washed; mounted in vectashield and stored at −20°C prior to confocal imaging.

Confocal imaging and analysis of synapses

Images were captured on the Zeiss LSM 700, using 405nm, 488nm and 546nm diode lasers. One z stack per section was taken at an interval of 0.1 μm across the central portion of the superficial dorsal horn, covering lamina 1-3 dorsoventrally. Approximately 50 optical sections per image were taken for analysis.

All image processing and analysis was performed in ImageJ. Z stacks were deconvolved using the WPL deconvolution algorithm in the Parallel Iterative Deconvolution ImageJ plugin (Wendykier and Nagy, 2010) based on the Iterative Deconvolve 3D algorithm, utilizing a theoretical 3D point spread function generated in PSF Lab. Image stacks were filtered with a 3x3 median filter and thresholded using the OTSU method. Lamina 1, 2outer, 2inner and 3 were defined using IB4 labeling, and saved to the ROI Manager. To assess synapse profile density in each lamina, individual z-slices separated by 0.4 μm were isolated and gephyrin+ & VGAT+ synaptic puncta profiles were analyzed separately and for each lamina using the Analyze Particles algorithm in ImageJ. Puncta profile counts in each lamina were averaged over three sections per animal, and plot as profile counts per 100 μm^2.

RNA isolation and cDNA synthesis

Mice were culled using a CO_2 chamber. Dissected DRGs were immediately frozen on liquid nitrogen and stored at −80. RNA was isolated using a combination of TriPure (Roche) and a High Pure RNA tissue kit (Roche). Briefly, tissue was homogenized in Tripure using a handheld homogenizer (Cole-Parmer) treated with chloroform and then subjected to column purification before being eluted in RNase free water. Synthesis of cDNA was carried out using Transcriptor reverse transcriptase (Roche), random hexamers (Invitrogen) and dNTPs (Roche).

Quantitative Real Time PCR

For analysis of mRNA expression using SYBR green cDNA (5ng) and primers (0.5μM) were mixed with LightCycler 480 SYBR Green Master (Roche) in a 1:1 ratio and added to white 384 well plates (Roche). Plates were run on a 45 cycle protocol using the LC 480 II system (Roche). Primers were designed using Primer-BLAST (https://www.ncbi.nlm.nih.gov/tools/primer-blast/). Primer efficiency and specificity were validated before experimental use. Gene expression for each target primer was normalized against 3 reference genes (18 s, GAPDH and HPRT1) using the delta delta CT method. Primer sequences are shown in Table S8. For mRNA expression analysis by means of Taqman technology, custom-made microfluidic Taqman array cards were designed. These cards contained primers and probes to detect a number of pain-related genes as well as 3 reference genes (18 s, GAPDH and HPRT1). Each cDNA sample was diluted in PCR grade water and added to Taqman Universal master mix to produce a final concentration of 1-4ng/μl. Cards were run on a 7900HT Fast Real-Time PCR system (Applied Biosystems) and gene expression calculated using the delta delta CT method. Assay IDs are shown in Table S8.

Electroporation

Electroporation was performed prior to plating using the Neon transfection system (Thermo Fisher Scientific). Dissociated neurons were resuspended at $5x10^6$ cells/ml in 10 μL Buffer R with 1 μg of total plasmid DNA. The electrical protocol applied was three 1500 V pulses of 10 ms duration. After electroporation cells were immediately plated as described above.

Plasmid construction

A plasmid containing full length (FL) human CASPR2 tagged with eGFP was generated as previously described (Irani et al., 2010). This plasmid was used to generate the plasmid containing the short form of CASPR2 (ENST00000463592.3). A PCR reaction was set up using DNA from the plasmid containing FL-CASPR2 and the short transcript isoform was amplified using the following primers GATCCTC GAGATGTCGTCCGCCACCGAC and GATCCCCGGGAAATGAGCCATTCCTTTTTGCTT. These primers were also used to add XhoI and XmaI restriction sites to the 5′ and 3′ ends, respectively. This product was run on an agarose gel and extracted using a DNA gel extraction kit (QIAGEN). The DNA plasmid was digested with XmaI and XhoI to remove the FL-CASPR2 sequence. The SH-CASPR2 sequence was then ligated into the cut plasmid using T4 ligase at 16°C overnight. The DNA plasmid was sequenced to confirm insert of the SH-CASPR2 isoform in frame with eGFP. A plasmid containing EGFP only was used as a control (pAcEGFP-C1, Clontech).

Cell based assay

Patient and health volunteer serum were initially tested for antibodies against CASPR2 and other related proteins such as LGI1, as previously described (Irani et al., 2010). Briefly, Human embryonic kidney 293 (HEK293) cells were cultured in Dulbeccos's modified Eagles's medium with 10% fetal calf serum (TCS Cellworks Ltd) in 6 well plates at 37°C. Penicillin G and streptomycin were added to the culture medium to prevent infection. Cells were transfected with a plasmid containing either full length or the short isoform of human CASPR2 tagged with eGFP. Forty-eight hours after transfection, live HEK cells were treated with diluted serum from patients or healthy control. Samples were diluted in Dulbecco's modified Eagles's medium buffered with HEPES plus 1% bovine serum albumin and a scoring system of 0-4 was used; where 0 indicated no human IgG binding and 4 very high levels of binding. Samples were titrated until a score of 0 was achieved. Titers indicate the last dilution when a score of 1 was given. This same protocol was used on human plasma and purified IgG as well as mouse samples.

Treatment of DRG neurons with human plasma

Plasma was obtained from both CASPR2-Ab positive patients. Before being used on cells, plasma was heated to 56°C for 30minutes to inactivate any potential complement and then placed on ice. Plasma was then added at a concentration of 1:100 to cell cultured medium and added to the cells 3 hours after plating. Electrophysiological recording were performed 1 and 2 days post plasma treatment. For analysis for Kv1.2 staining, cells were fixed 1 day after treatment.

Quantification of membrane Kv1.2

For analysis of DRG neurons from *Cntnap2*$^{-/-}$ mice, cells were cultured for 1 day. For plasma analysis, cells were cultured in the presence of plasma for 1 day. Following fixation DRG coverslips were treated with primary antibodies against Kv1.2, NF200 and Beta-III Tubulin and the appropriate secondary antibodies (Table S7). Coverslips were then imaged using a confocal microscope and around 20 images were taken from each coverslip using the x40 objective. Using ImageJ software profile plots of each cell were made spanning the cell diameter for kv1.2 immunoreactivity and a background reading was also taken. An average of the signal intensity was then taken for the portion of the plot relating the membrane and that relating to the cytoplasm (avoiding the nucleus). Only signal intensities greater than background were used. A ratio of membrane to cytoplasm was calculated and those cells with greater than 1.5 times cytoplasm intensity were defined as being kv1.2 membrane positive.

Electrophysiology

Patch clamping of DRG neurons in vitro

Whole-cell patch clamp recordings were performed at room temperature (22°) using an Axopatch 200B amplifier and Digidata 1550 acquisition system (Molecular Devices). GFP$^+$ DRG were detected with an Olympus microscope with an inbuilt GFP filter set

(470/40x excitation filter, dichroic LP 495 mirror and 525/50 emission filter). Data were low-pass filtered at 2 kHz and sampled at 10 kHz. Series resistance was compensated 70%–90% to reduce voltage errors. Patch pipettes (2–4 MΩ) were pulled from filamental borosilicate glass capillaries (1.5 mm OD, 0.84 mm ID; World Precision Instruments). *Current clamp;* Patch pipettes were filled with internal solution containing (mM): 130 KCl, 1 $MgCl_2$, 5 MgATP, 10 HEPES, and 0.5 EGTA; pH was adjusted to 7.3 with KOH and osmolarity set to 305 mOsm. Extracellular solution contained (mM): 140 NaCl, 4.7 KCl, 1.2 $MgCl_2$, 2.5 $CaCl_2$, 10 HEPES and 10 glucose; pH was adjusted to 7.3 with NaOH and osmolarity was set to 315 mOsm. α-Dendrotoxin (α–DTX, Alomone) was prepared as a 10,000x stock in H_2O and added via the perfusion system. Unless otherwise stated, post-DTX recordings were always made 5 minutes after addition of the drug. Resting membrane potential was assessed in bridge mode, while firing properties were assessed in current clamp mode. Input resistance (R_{Input}) was calculated from the voltage deflections caused by increasing (Δ20 pA) hyperpolarising current pulses. To determine rheobase, cells were depolarised from a holding potential of −60 mV by current steps (50 ms) of increasing magnitude (Δ25 pA) until an action potential was generated. Repetitive firing was assessed by 500 ms depolarising current steps of increasing magnitude (50pA). *Voltage clamp*. Patch pipettes were filled with internal solution containing (mM): 120 K^+ gluconate, 20 KCl, 2 $MgCl_2$, 10 EGTA, 10 HEPES, 1 $CaCl_2$ and 5 MgATP; pH was adjusted to 7.3 with KOH and osmolarity was set to 305 mOsm. Extracellular solution contained (mM): 150 Choline-Cl, 5 KCl, 2 $CaCl_2$, 1 $MgCl_2$, 10 HEPES, 0.1 $CdCl_2$ and 10 glucose; pH was adjusted to 7.4 with KOH and osmolarity was set to 315 mOsm. Outward currents were elicited by depolarising the membrane potential from −70 to +40mV for 500ms in 10mV increments, following a 1 s pre-pulse conditioning step to −40mV. The outward current generated at the end of the depolarising pulse was taken as I_{KD}. DTX sensitive currents were obtained by subtracting I_{KD} post-DTX from pre-drug levels. Data were analyzed by Clampfit 10 software (Molecular Devices)

Ex vivo *skin-nerve preparation*

Skin-nerve primary afferent recordings were conducted in a similar fashion to Milenkovic et al. (2014). Briefly, the hind paw glabrous skin and tibial nerve were dissected from adult male and female *Cntnap2*$^{+/+}$ (n = 16) and *Cntnap2*$^{-/-}$ (n = 15) mice. The skin was maintained in a perfusion chamber in the outside out configuration (epidermis facing up). The chamber was constantly perfused with synthetic interstitial fluid (SIF: 2.0mM CaCl2, 5.5mM Glucose, 10mM HEPES, 3.5mM KCL, 0.7 mM MgSO4, 123mM NaCl, 1.5mM NaH2PO4, 9.5mM Na-gluconate, 7.5mM Sucrose, 1M NaOH; dH_20) at 32°C. Nerve filaments from the tibial nerve were separated and placed onto a silver recording electrode. To assess conduction velocities and to characterize fiber types, receptive fields were electrically stimulated with pulsed supra-threshold currents. The skin was stimulated mechanically using a 0.8mm diameter probe attached to a piezo electric stimulator (Physik Instrument), or a NanoMotor stimulator (Kleindiek), both in conjunction with force sensors (Kleindiek). Stimuli of increasing velocities or forces were applied to identify receptive fields on the skin. Stimulus response functions and AP adaptation properties were analyzed in both the ramp and hold phase of the mechanical stimulus. All stimuli evoked action potentials were visualized using an oscilloscope and recorded using a Powerlab 4.0 system in conjunction with LabChart v7.3 software (ADInstruments).

Extracellular dorsal horn recording

In vivo electrophysiology was conducted on *Cntnap2*$^{+/+}$ and *Cntnap2*$^{-/-}$ male and female mice aged between 9 and 11 weeks old. Animals were initially anaesthetised with 3.5% v/v isofluorane delivered in 3:2 ratio of nitrous oxide and oxygen. Once areflexic, mice were secured in a stereotaxic frame and subsequently maintained on 1.5% v/v isofluorane for the remainder of the experiment. A laminectomy was performed to expose L3-L5 segments of the spinal cord. Extracellular recordings were made from deep dorsal horn wide dynamic range (WDR) lamina V/VI neurones with receptive fields on the glabrous skin of the toes using 0.127 mm 2 MΩ parylene-coated tungsten electrodes (A-M Systems). Neurones were characterized from depths relating to deep dorsal horn laminae (350-700 μm from surface of cord), and were selected on the basis of responses to dynamic brushing, and noxious mechanical and thermal stimulation. Electrical stimulation of WDR neurones was delivered transcutaneously via needles inserted into the receptive field. A train of 16 electrical stimuli (2 ms pulses, 0.5 Hz) was applied at three times the threshold current for C-fiber activation. Responses evoked by A- (0–50 ms) and C-fibers (50–250 ms) were separated and quantified on the basis of latency. Neuronal responses occurring after the C-fiber latency band were classed as post-discharge (PD). The input (I) and the wind-up (WU) were calculated as: Input = (action potentials evoked by first pulse) × total number of pulses (16), wind-up = (total action potentials after 16 train stimulus) − Input. The receptive field was also stimulated using a range of natural stimuli (brush, von Frey filaments – 0.4, 1, 4, 8 and 15 g and heat – 32, 37, 42, 45 and 48°C) applied over a period of 10 s per stimulus and the evoked response quantified. The heat stimulus was applied with a constant water jet onto the center of the receptive field. Acetone (Sigma) and ethyl chloride (50 μl) (Miller Medical Supplies) were applied to the receptive field, described previously as an evaporative innocuous cooling and noxious cooling stimulus respectively. Evoked responses to room temperature water (25°C) were subtracted from acetone and ethyl chloride evoked responses to control for any concomitant mechanical stimulation during application. Natural stimuli were applied starting with the lowest intensity stimulus with approximately 30 s between stimuli in the following order: brush, von Frey, cold, heat, electrical. Receptive fields were determined using a pinch stimulus. An area was considered part of the receptive field if a response of > 30 action potentials over 5 s were obtained. A rest period of 30 s between applications was used to avoid sensitization. Receptive field sizes are expressed as a percentage area of a standardized paw measured using ImageJ (NIH, Bethesda). Data were captured and analyzed by a Cambridge Electronic Design 1401 interface coupled to a computer with Spike2 software (CED, Cambridge, UK) with post-stimulus time histogram and rate functions. A total of 11 neurones were characterized from 7 *Cntnap2*$^{+/+}$ mice, and 10 neurones from 7 *Cntnap2*$^{-/-}$ mice.

Spinal cord slice preparations

Cntnap2$^{+/+}$ (n = 7) and *Cntnap2*$^{-/-}$ (n = 6) mice 5-8 weeks old were decapitated under general anesthesia with isoflurane (1%–3%). The spinal cord was isolated in ice cold dissecting solution that contained the following (in mM): 3.0 KCl, 1.2 NaH_2PO_4, 0.6 $CaCl_2$, 6.5 $MgCl_2$, 26 $NaHCO_3$, 25 glucose, 240 sucrose, oxygenated with 95% O_2 and 5% CO_2. The dura mater was removed and ventral and dorsal roots were trimmed from the cord. The lumbar segment was cut in parasagittal slices (300 μm) with a vibrating blade microtome (MicromHM 650V, Fisher Scientific). Slices were kept at room temperature for at least 30 min in recording solution that contained the following (in mM): 127 NaCl, 3.0 KCl, 1.2 NaH_2PO_4, 2.4 $CaCl_2$, 1.3 $MgCl_2$, 26.0 $NaHCO_3$, 15 glucose, oxygenated with 95% O_2, 5% CO_2.

Cells targeted for patch-clamp recording were selected within the superficial dorsal horn (mostly lamina II), which was visualized under infrared differential interference contrast microscopy on an Olympus BX51WI microscope. Patch pipettes were pulled with a horizontal puller (P-97, Sutter Instruments) from glass capillaries (World Precision Instruments). The pipettes had an electrical resistance of 4 - 6MΩ when filled with internal solution that contained the following (in mM): 130 potassium gluconate, 10 KCl, 2 $MgCl_2$, 10 HEPES, 0.5 EGTA, 2 ATP-Na_2, 0.5 GTP-Na, pH adjusted to 7.3 with 1 M KOH. Patch-clamp signals were amplified and filtered (4 kHz low-pass Bessel filter) with a MultiClamp 700B amplifier (Molecular Devices) and acquired at 10 kHz using a Digidata 1440 A A/D board and pClamp 10 software (Molecular Devices).

Spontaneous EPSCs (sEPSCs) were recorded in dorsal horn neurons which were voltage-clamped at −60mV. To excite central terminals of TRPV1 positive C-fibers, capsaicin (1 μM) was bath applied. Three minutes of raw traces were selected for analysis, in baseline and during peak evoked sEPSC response (typically a few minutes after the start of bath application of capsaicin). sEPSCs were detected offline, using Mini Analysis Program software (Synaptosoft).The investigator was blinded to the genotype of the animals until analysis of each recorded cell was complete. A total of 15 cells was collected from *Cntnap2*$^{-/-}$ mice and 16 cells from *Cntnap2*$^{+/+}$ animals. To assess whether there was a selective response to excitation of central C-fiber terminals either in *Cntnap2*$^{-/-}$ and *Cntnap2*$^{+/+}$ mice, the frequency of capsaicin evoked sEPSCs was analyzed. Also, the mean amplitude of these evoked sEPSCs was compared, between genotypes.

AAV injections for DRG GCaMP6 expression

Mice were anaesthetised using isoflurane (2% O_2) and their body temperature maintained around 37°C. After an initial skin incision over the lumbar region of the spinal cord the intervertebral space between T12 and T13 was exposed. A small catheter (0.2 mm Ø, Braintree Scientific) was inserted under the dura in the caudal direction through a small cut. A volume of 5ul of AAV9.CAG. GCaMP6s.WPRE.SV40 (UPENN Vector Core, AV-1-PV2833, 1.1x1013 g/ml) was infused at a rate of 1.2μl/min after which the cannula remained subdural for a further 2 minutes before withdrawal. The skin was sutured and Carprieve (0.025 mg; Norbrook Laboratories) was administered subcutaneously for post-operative pain management. The mice were allowed to recover for 0.5-2months before *in vivo* calcium imaging

In vivo calcium imaging of DRG

Cntnap2$^{+/+}$ (n = 4) and *Cntnap2*$^{-/-}$ (n = 5) mice were anaesthetised using urethane (12.5%w/v) injected intraperitoneally (IP). The initial dose of 37.5mg (0.3ml) was administered to all mice and further doses (approximately 15 minutes apart) were titrated to the level of anesthesia until surgical depth was achieved. Core body temperature was maintained around 37°C using a homeothermic heating mat (FHC) with feedback from a rectal probe. A tracheal tube was installed but mice breathed freely.

Hair was removed and an incision was made in the skin between L3 – L5 spinal segments. The connective tissue and muscle were removed on and around the vertebrae and a small laterally extended laminectomy was performed around the L4 DRG. The dura and perineurium were left intact and covered using silicone elastomer (World Precision Instruments, Ltd). The spinal column was clamped using vertebral clamps (Precision Systems and Instrumentation) and stabilized on a custom made stage. An Eclipse Ni-E FN upright confocal/multiphoton microscope (Nikon) was used to acquire images using a 10X dry objective. A 488 nm Argon ion laser line was used for excitation and GCaMP signal was collected at 500-550 nm. Time series recordings were typically recorded at 4hz with an in-plane resolution of 512 × 512 pixels and a fully open pinhole.

While imaging the response of the DRG, eight brush stokes were applied onto the ipsilateral paw at approximately 0.5hz in the medial to lateral direction. Pinch was applied with an arterial clamp (World Precision Instruments, Ltd) in 4 positions on the paw: at the ankle, between the ankle and the walking pads, along the walking pads and across the toes. Thermal stimulation (50°C) was applied to the hind paw through a Peltier device (TSAII, Medoc) with a 16x16mm probe. Capsaicin cream (10%) was applied at the end of the experiment to both the hairy and plantar surface of the skin and assessed for 5 minutes after application was completed.

The acquired images were drift corrected using NIS Elements AR 4.30.01 (Nikon, align application). The raw data was background subtracted and normalized by subtracting the baseline and dividing the difference with the baseline to generate ΔF/F. Regions of interest (ROIs) around neuronal cell bodies were chosen using a free hand selection tool in Fiji/ImageJ Version 1.48v. ROIs to determine GCaMP fluorescence were chosen stringently, with minimal overlap to ensure less interference from surrounding somata. ROIs for calculation of neuronal cell body size were then chosen with less stringency, allowing for overlapping regions in which cells are in close proximity, to reflect a more accurate calculation of cell diameter. Responding cells (if their response was 70% above baseline florescence plus 4 standard deviations (STDEV)) were binned into 200μm^2 sizes (where the cell body profile was determined by the

GCaMP expression) and compared between *Cntnap2*$^{+/+}$ and *Cntnap2*$^{-/-}$ mice. To assess spontaneous activity, traces of neuronal GCaMP fluorescence (as $\Delta F/F$), from the first 8 minutes of baseline, were generated for each neuron. A blinded examiner visually determined the presence or absence of spontaneous activity, which was expressed as a percentage of spontaneously active neurons per total number of neurons in each DRG.

QUANTIFICATION AND STATISTICAL ANALYSIS

Data is shown as the mean ± SEM, unless otherwise stated. A Student's t test was used to compare the mean of two groups and when data was not normally distributed a non-parametric test was applied (Mann-Whitney). A one-way ANOVA was used when more than two groups existed. For behavioral studies over time, a repeated-measures two-way ANOVA was used with Holm-Sidak posthoc analysis. For *In vivo* calcium imaging an ANOVA on ranks was performed. For patch clamp experiments multiple Mann-Whitney tests were used for repetitive firing assays on DRG cells and a two-way ANOVA with Holm-Sidak posthoc analysis for current voltage relationships. For pre and post DTX treatment a paired t test was performed. For *ex vivo* skin nerve preparation data a two-way ANOVA or a two-way repeated-measure ANOVA with Bonferroni posthoc analysis was used. For dorsal horn recordings a two-way repeated-measure ANOVA with Bonferroni posthoc analysis was applied. Sample sizes for each experimental can be found in the figure legend. Significance for all experiments was placed at $p < 0.05$. Statistical tests were carried out with GraphPad prism or SigmaStat.

Neuron, Volume *97*

Supplemental Information

Immune or Genetic-Mediated Disruption of CASPR2 Causes Pain Hypersensitivity Due to Enhanced Primary Afferent Excitability

John M. Dawes, Greg A. Weir, Steven J. Middleton, Ryan Patel, Kim I. Chisholm, Philippa Pettingill, Liam J. Peck, Joseph Sheridan, Akila Shakir, Leslie Jacobson, Maria Gutierrez-Mecinas, Jorge Galino, Jan Walcher, Johannes Kühnemund, Hannah Kuehn, Maria D. Sanna, Bethan Lang, Alex J. Clark, Andreas C. Themistocleous, Noboru Iwagaki, Steven J. West, Karolina Werynska, Liam Carroll, Teodora Trendafilova, David A. Menassa, Maria Pia Giannoccaro, Ester Coutinho, Ilaria Cervellini, Damini Tewari, Camilla Buckley, M. Isabel Leite, Hendrik Wildner, Hanns Ulrich Zeilhofer, Elior Peles, Andrew J. Todd, Stephen B. McMahon, Anthony H. Dickenson, Gary R. Lewin, Angela Vincent, and David L. Bennett

Figure S1

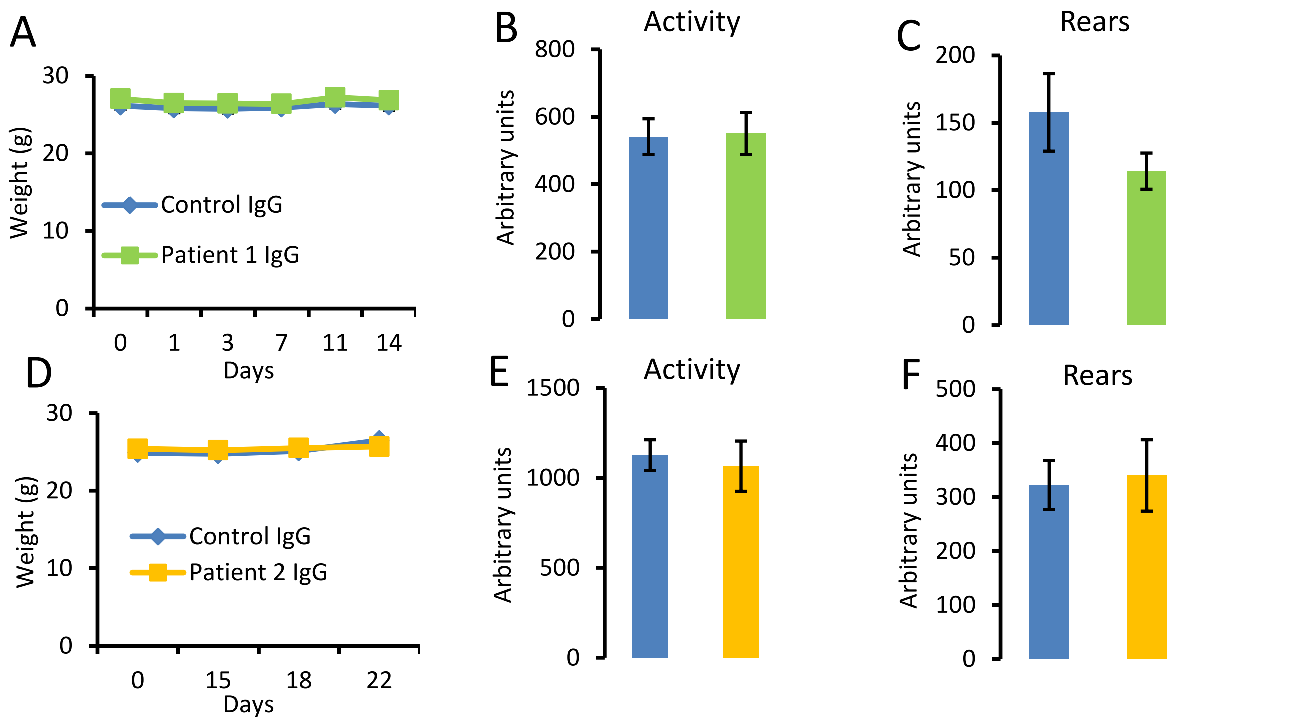

A
30
20
10
0
Weight (g)
Control IgG
Patient 1 IgG
0
1
3
7
11
14
Days
B
Activity
800
600
400
200
0
Arbitrary units
C
Rears
200
150
100
50
0
Arbitrary units
D
30
20
10
0
Weight (g)
Control IgG
Patient 2 IgG
0
15
18
22
Days
E
Activity
1500
1000
500
0
Arbitrary units
F
Rears
500
400
300
200
100
0
Arbitrary units

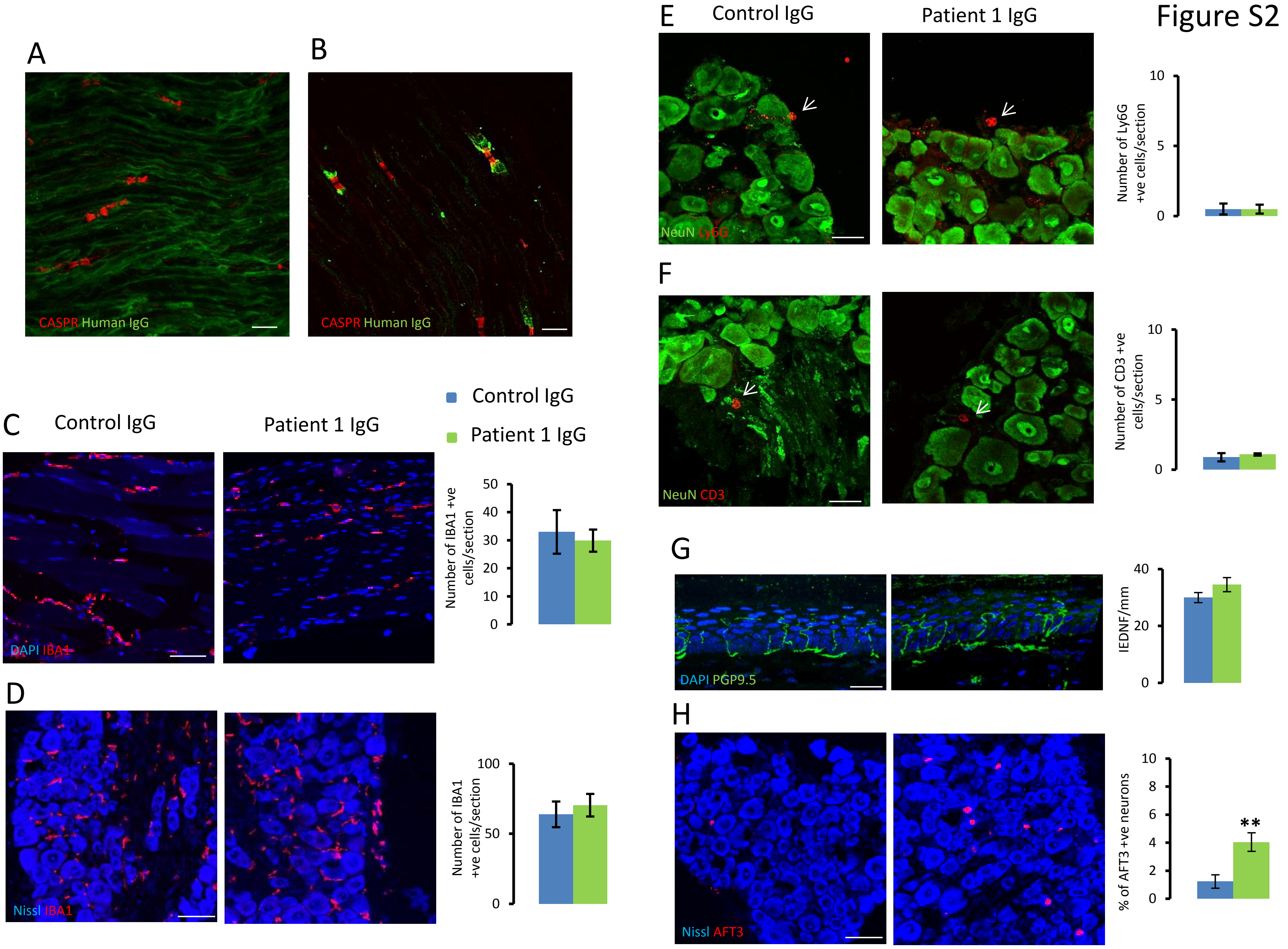
Figure S2
A
B
CASPR Human IgG
CASPR Human IgG
C
Control IgG
Patient 1 IgG
DAPI IBA1
Number of IBA1 +ve cells/section
D
Nissl IBA1
Number of IBA1 +ve cells/section
E
Control IgG
Patient 1 IgG
NeuN Ly6G
Number of Ly6G +ve cells/section
F
NeuN CD3
Number of CD3 +ve cells/section
G
DAPI PGP9.5
IEDNF/mm
H
Nissl AFT3
% of AFT3 +ve neurons
**

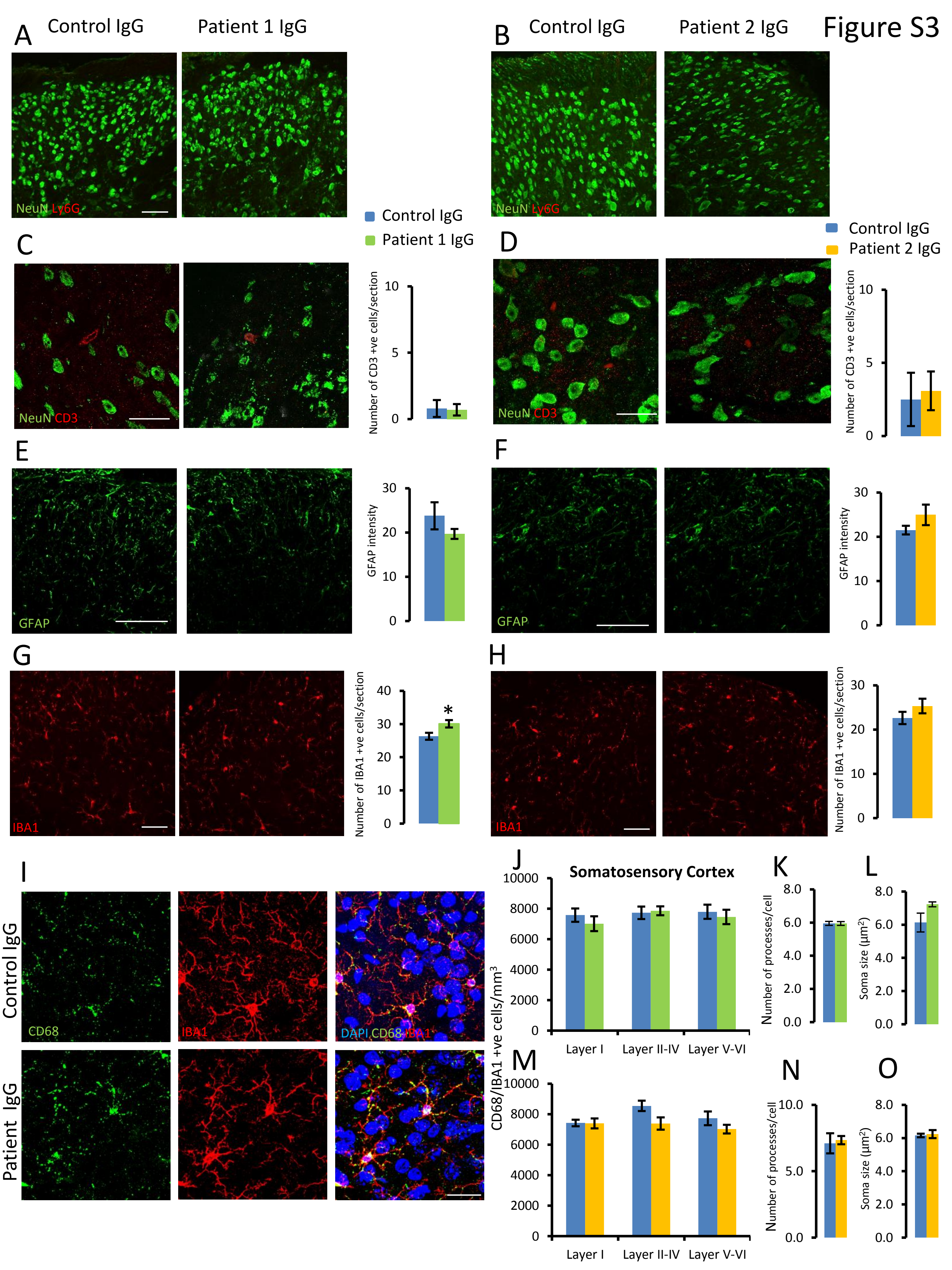

Figure S3
A
Control IgG
Patient 1 IgG
NeuN Ly6G
B
Control IgG
Patient 2 IgG
NeuN Ly6G
C
Control IgG
Patient 1 IgG
NeuN CD3
Number of CD3 +ve cells/section
D
Control IgG
Patient 2 IgG
NeuN CD3
Number of CD3 +ve cells/section
E
GFAP
GFAP intensity
F
GFAP
GFAP intensity
G
IBA1
Number of IBA1 +ve cells/section
H
IBA1
Number of IBA1 +ve cells/section
I
Control IgG
Patient IgG
CD68
IBA1
DAPI CD68 IBA1
J
Somatosensory Cortex
CD68/IBA1 +ve cells/mm³
Layer I
Layer II-IV
Layer V-VI
K
Number of processes/cell
L
Soma size (µm²)
M
N
O

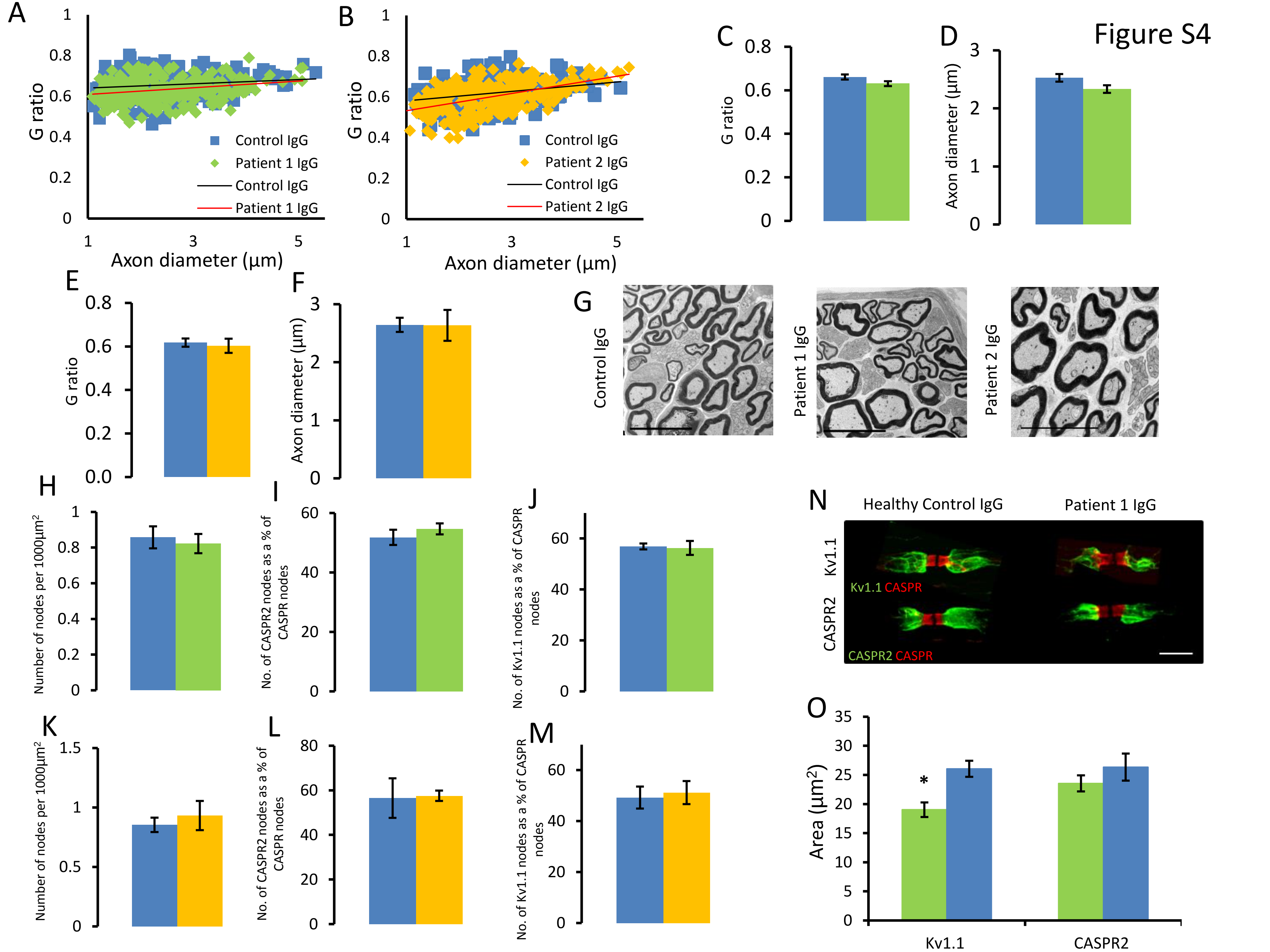

Figure S4
A
G ratio
Axon diameter (μm)
Control IgG
Patient 1 IgG
Control IgG
Patient 1 IgG
B
G ratio
Axon diameter (μm)
Control IgG
Patient 2 IgG
Control IgG
Patient 2 IgG
C
G ratio
D
Axon diameter (μm)
E
G ratio
F
Axon diameter (μm)
G
Control IgG
Patient 1 IgG
Patient 2 IgG
H
Number of nodes per 1000μm2
I
No. of CASPR2 nodes as a % of CASPR nodes
J
No. of Kv1.1 nodes as a % of CASPR nodes
N
Healthy Control IgG
Patient 1 IgG
Kv1.1
Kv1.1 CASPR
CASPR2
CASPR2 CASPR
K
Number of nodes per 1000μm2
L
No. of CASPR2 nodes as a % of CASPR nodes
M
No. of Kv1.1 nodes as a % of CASPR nodes
O
Area (μm2)
*
Kv1.1
CASPR2

Figure S5

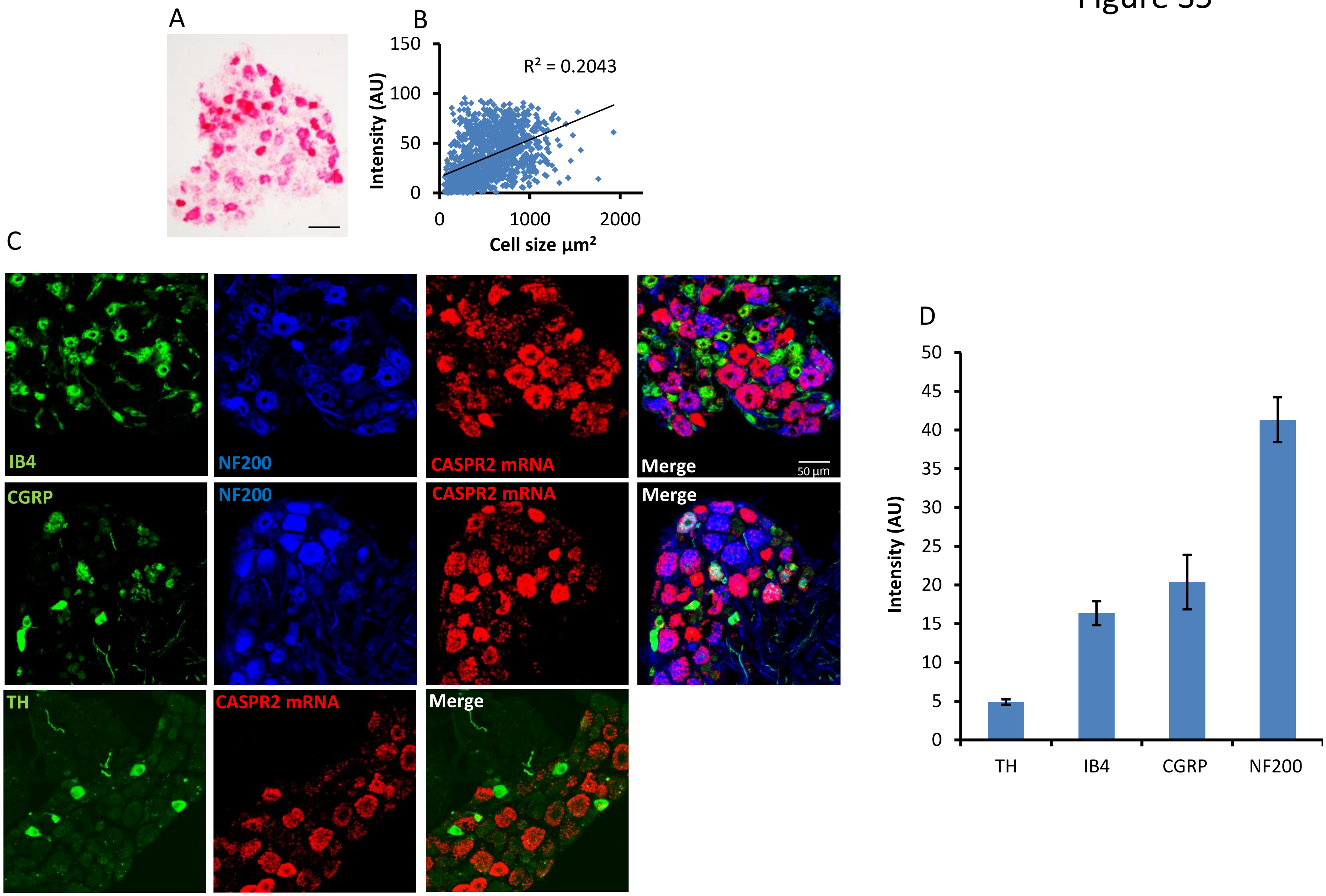
A
B
150
100
50
0
Intensity (AU)
R² = 0.2043
0
1000
2000
Cell size µm²
C
IB4
NF200
CASPR2 mRNA
Merge
50 µm
CGRP
NF200
CASPR2 mRNA
Merge
TH
CASPR2 mRNA
Merge
D
50
45
40
35
30
25
20
15
10
5
0
Intensity (AU)
TH
IB4
CGRP
NF200

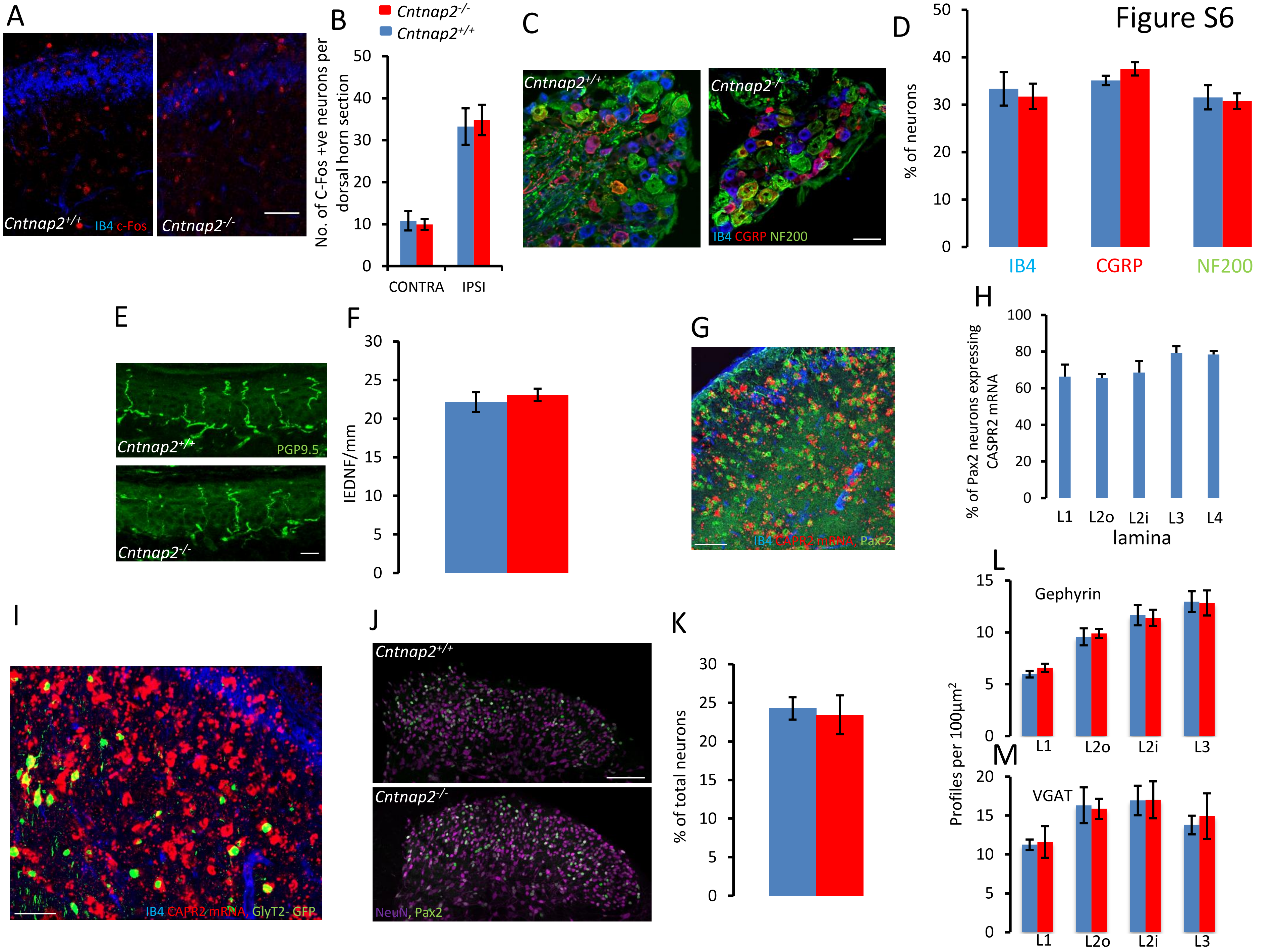

Figure S6
A
Cntnap2+/+ IB4 c-Fos
Cntnap2-/-
B
Cntnap2-/-
Cntnap2+/+
No. of C-Fos +ve neurons per dorsal horn section
CONTRA
IPSI
C
Cntnap2+/+
Cntnap2-/-
IB4 CGRP NF200
D
% of neurons
IB4
CGRP
NF200
E
Cntnap2+/+
PGP9.5
Cntnap2-/-
F
IEDNF/mm
G
IB4 CAPR2 mRNA, Pax-2
H
% of Pax2 neurons expressing CASPR2 mRNA
L1
L2o
L2i
L3
L4
lamina
I
IB4 CAPR2 mRNA, GlyT2- GFP
J
Cntnap2+/+
Cntnap2-/-
NeuN, Pax2
K
% of total neurons
L
Gephyrin
M
VGAT
Profiles per 100µm²

Figure S7

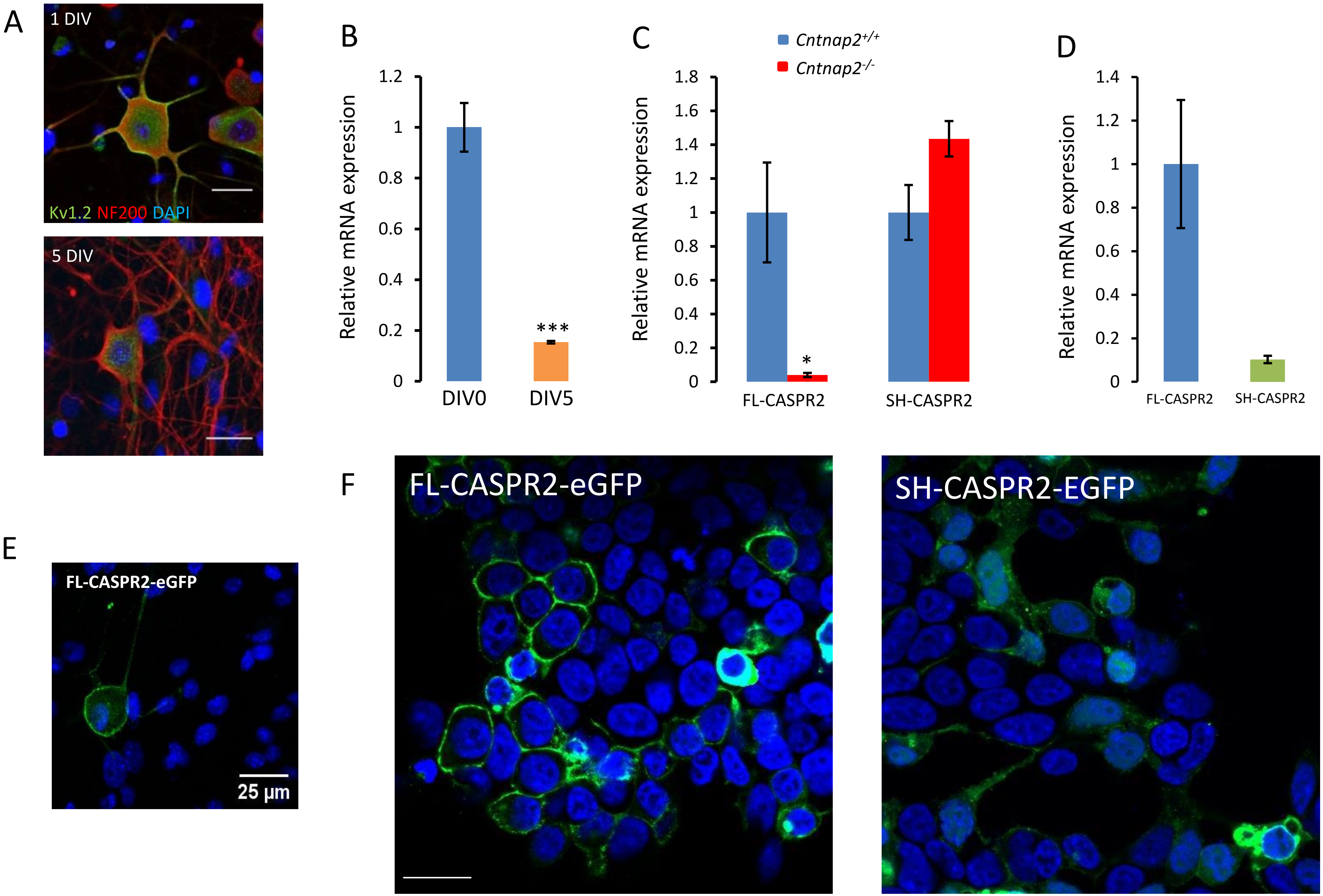
A
1 DIV
Kv1.2 NF200 DAPI
5 DIV
B
Relative mRNA expression
1.2
1
0.8
0.6
0.4
0.2
0
***
DIV0
DIV5
C
Cntnap2+/+
Cntnap2-/-
Relative mRNA expression
1.8
1.6
1.4
1.2
1
0.8
0.6
0.4
0.2
0
*
FL-CASPR2
SH-CASPR2
D
Relative mRNA expression
1.4
1.2
1
0.8
0.6
0.4
0.2
0
FL-CASPR2
SH-CASPR2
E
FL-CASPR2-eGFP
25 µm
F
FL-CASPR2-eGFP
SH-CASPR2-EGFP

Figure S8

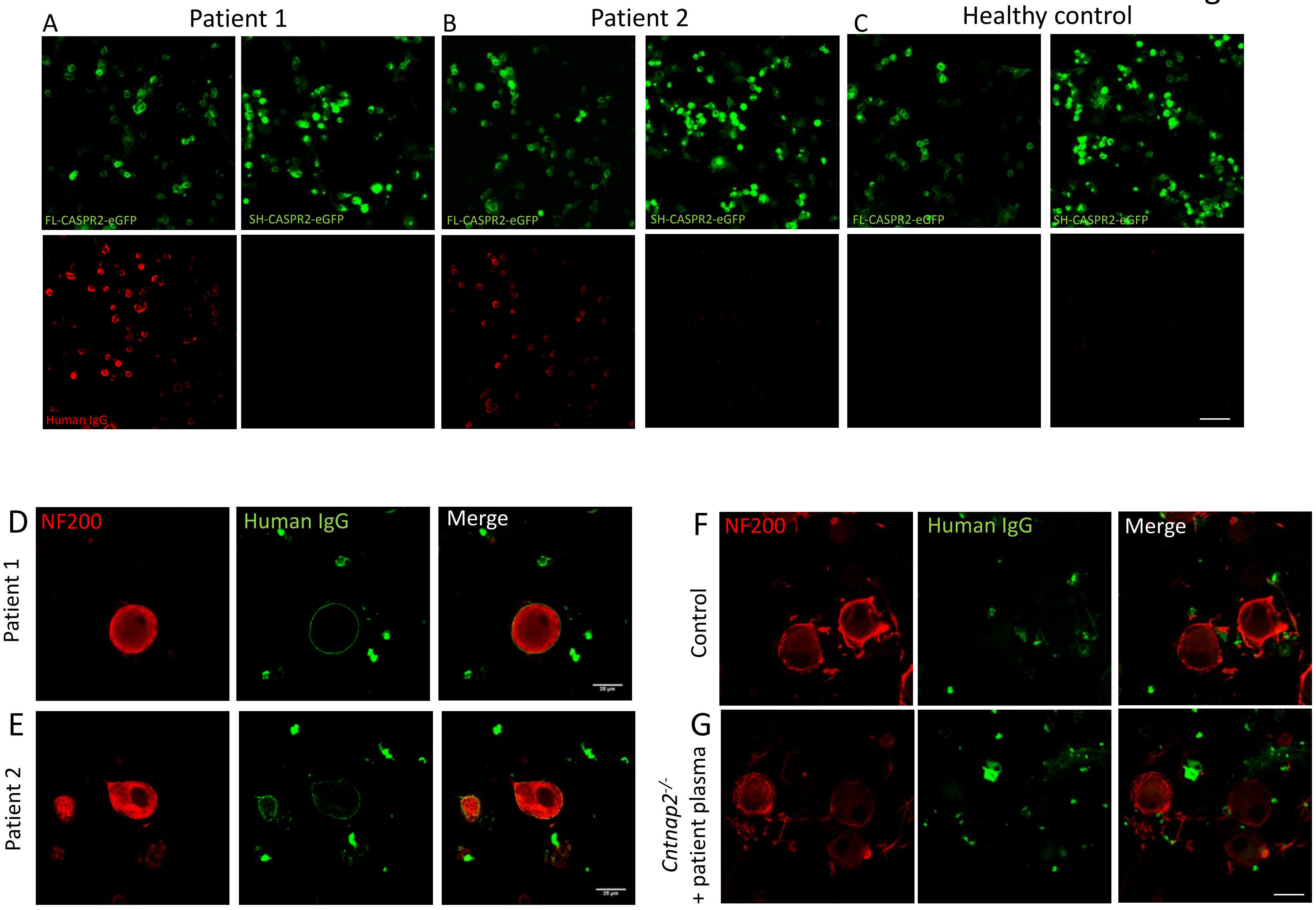

A
Patient 1
B
Patient 2
C
Healthy control
FL-CASPR2-eGFP
SH-CASPR2-eGFP
FL-CASPR2-eGFP
SH-CASPR2-eGFP
FL-CASPR2-eGFP
SH-CASPR2-eGFP
Human IgG
D
NF200
Human IgG
Merge
Patient 1
E
Patient 2
F
NF200
Human IgG
Merge
Control
G
Cntnap2-/-
+ patient plasma

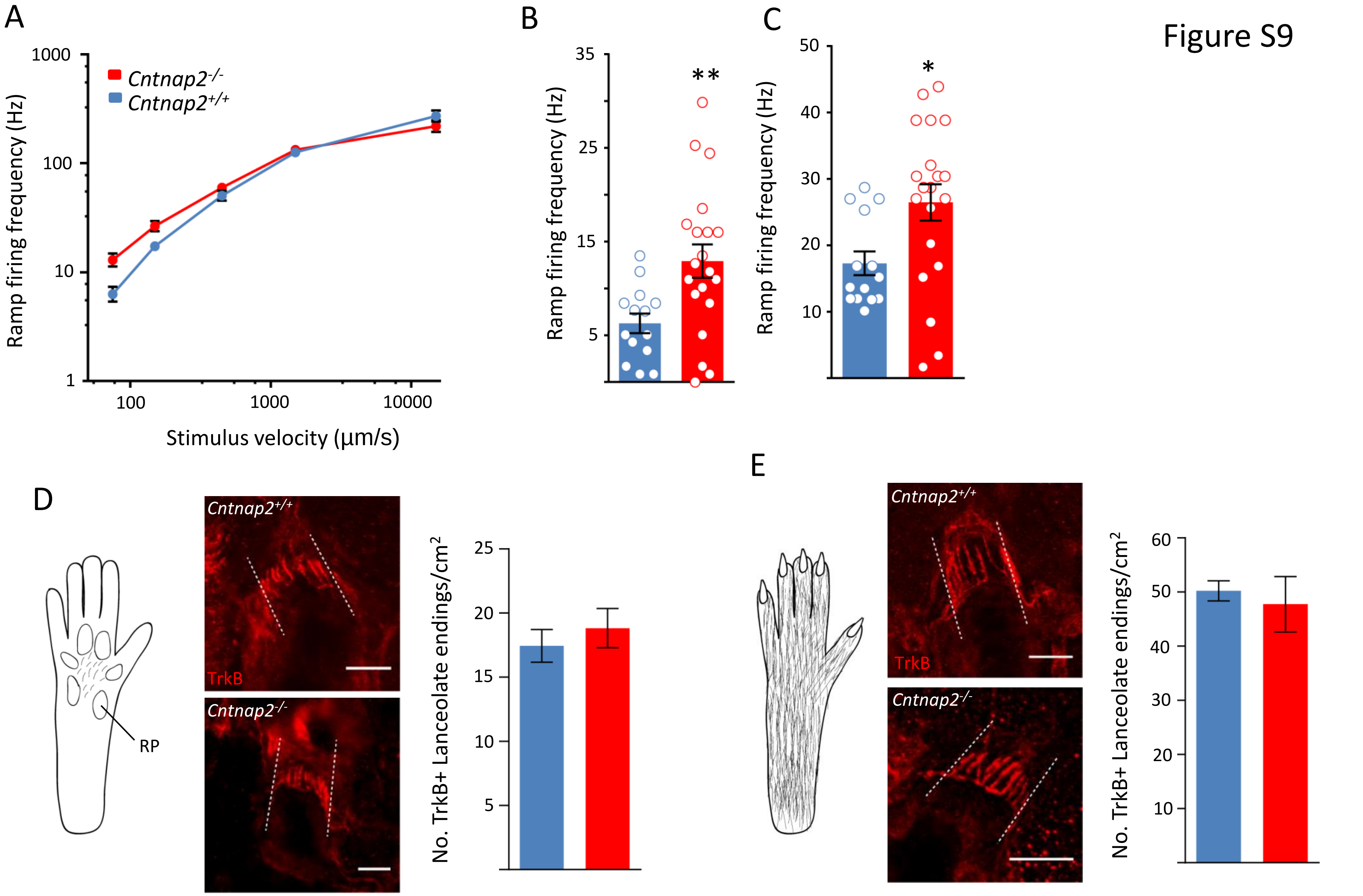

Figure S9
A
Cntnap2-/-
Cntnap2+/+
Ramp firing frequency (Hz)
1000
100
10
1
100
1000
10000
Stimulus velocity (µm/s)
B
Ramp firing frequency (Hz)
35
25
15
5
**
C
Ramp firing frequency (Hz)
50
40
30
20
10
*
D
RP
Cntnap2+/+
TrkB
Cntnap2-/-
No. TrkB+ Lanceolate endings/cm2
25
20
15
10
5
E
Cntnap2+/+
TrkB
Cntnap2-/-
No. TrkB+ Lanceolate endings/cm2
60
50
40
30
20
10

Figure S10

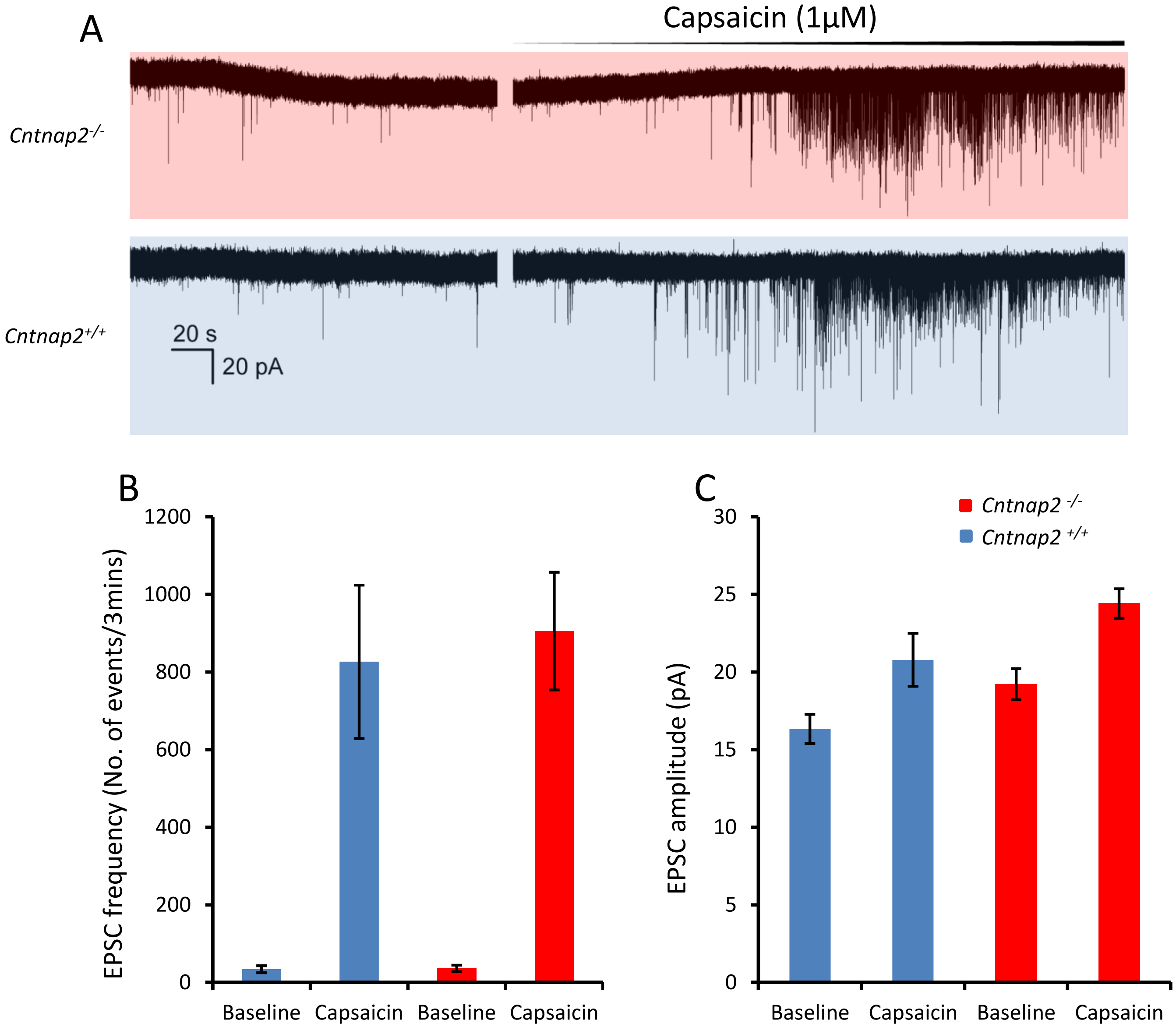
A
Capsaicin (1μM)
Cntnap2-/-
Cntnap2+/+
20 s
20 pA
B
EPSC frequency (No. of events/3mins)
1200
1000
800
600
400
200
0
Baseline
Capsaicin
Baseline
Capsaicin
C
Cntnap2 -/-
Cntnap2 +/+
EPSC amplitude (pA)
30
25
20
15
10
5
0
Baseline
Capsaicin
Baseline
Capsaicin

Supplementary Figure 1 Mice treated with patient IgG do not develop sickness and display normal motor activity. Related to Figure 1

(A and D) Weight measurements over time for mice treated with IgG from CASPR2-Ab positive patients or healthy control.

(B and E) Locomotor activity was assessed using the open field test in mice treated with IgG from CASPR2-Ab positive patients or healthy control. An area under the curve analysis of the number of boxes entered within a 3 minute period was used to incorporate data taken from multiple time points across the dosing regime.

(C and F) Rearing behaviour measured during the open field test, data shown as area under the curve to incorporate data taken from multiple time points across the dosing regime.

For A-C, n=8, for D-F n=9. All data shown as mean ± SEM

Supplementary Figure 2 Passive transfer of patient CASPR2-Abs does not cause gross inflammation or damage to the mouse peripheral nerve. Related to Figure 2

(A) Sciatic nerve section from a mouse treated in vivo with Patient IgG. CASPR (red) is used to mark the paranode and there is no specific binding to nodal regions of human IgG (green). Scale bar 10µm.

(B) Sciatic nerve section taken from WT mouse, permeabilised with Triton-X to allow access to the JXP and subsequently treated with Patient IgG. Clear binding of human IgG to the JXP can be seen using IgG from a CASPR2-Ab positive patient. Scale bar 10µm.

(C and D) Representative images of sciatic nerve (C) and DRG (D) sections from mice treated with either control or Patient 1 IgG. Nissl or DAPI in blue, the macrophage marker IBA1 shown in red. Quantification shows no difference between the numbers of IBA1 positive cells between treatment groups. Data shown as the average number of IBA1 positive cells per image, 3 images per animal, n=5. Scale bar 50µm

(E and F) Representative images of DRG sections from mice treated with either control or Patient 1 IgG stained for the neutrophil marker Ly6G (red, E) and the T cell marker CD3 (red, F). The number of positive cells was very low and no difference was found between groups. Data shown as the average number of cells per DRG section, 3-4 images per animal, n=4. Scale bar 25 µm.

(G) Representative images of mouse glabrous skin. PGP9.5 (green) was used to mark nerve fibres, DAPI shown in blue. Quantification showed no difference in the number of IEDNFs between treatment groups, 3 images per animal, n=5. Scale bar 25µm

(H) Representative images of mouse DRG sections immunostained for the injury marker ATF3 (red). Quantification showed a small but significant increase in ATF3 positive neurons in those mice treated with patient 1 IgG versus controls. The number of ATF3 positive cells shown as a percentage of nissl (blue) positive neurons, 3-4 images per animal, n=5. Scale bar 50µm.

For H Students t test, $^{**}p<0.01$ versus Control IgG group. All data shown as mean ± SEM

Supplementary Figure 3 Passive transfer of patient CASPR2-Abs does not cause a cellular inflammatory response in the CNS. Related to Figure 2

(A and B) Representative images of spinal cord stained for the neutrophil marker Ly6G (red) from mice treated with either control, Patient 1 (A) or Patient 2 IgG (B). No neutrophils were detected in the spinal cord, scale bar 50 µm.

(C and D) Very few CD3 (red) positive cells (a marker of T cells) were detected in spinal cord from control or Patient IgG treated mice (C and D, Patient 1 and 2 IgG groups respectively). No difference was found in the number of T cells between treatment groups. Data shown as the average number of CD3 positive cells per section, 3-4 images per animal, n=4. Scale bar 25 µm.

(E and F) Representative images of GFAP (green) intensity, used as an indicator of astrocyte reactivity, in spinal cord sections from mice treated with control versus Patient 1 (E) and Patient 2 IgG (F). No significant difference was seen between treatment groups. Data shown as the average intensity of GFAP staining per section, 3-4 images per animal, n=4. Scale bar 50 µm.

(G and H) Representative images of microglia, stained using the marker IBA1 (red), in spinal cord sections taken from mice treated with control versus Patient 1 (G) or Patient 2 (H) IgG. Quantification shows a small but significant increase in microglia number in the spinal cord from mice treated with Patient 1 IgG versus control. No difference was seen for Patient 2 IgG. Data shown as the average number of IBA1 positive cells per section, 3-4 images per animal, n=4. Scale bar 50 µm.

(I) Representative images of CD68 (a marker of microglia activation, green) and IBA1 (red) colocalisation in the somatosensory cortex of mice treated with control or Patient IgG. DAPI is shown in blue, scale bar 20 µm.

(J and M) No difference was found in the density of CD68/IBA1 positive microglia in layer I, Layers II-IV and Layers V-VI of the somatosensory cortex in mice treated with Patient 1 (J) or Patient 2 IgG (M) versus control, n=4.

(K, L, N & O) Morphological analysis of microglia showed no difference between treatment groups. There was no difference in the number of processes per cell between microglia in control mice and mice treated with either Patient 1 (K) or Patient 2 (N) IgG. And there was no difference in the soma size of microglia in control versus Patient 1 (L) or Patient 2 (O) treated mice.

For G Students t test, *$p<0.05$ versus Control IgG group. All data shown as mean ± SEM

Supplementary Figure 4 Analysis of peripheral nerve ultrastructure and nodes in mice treated with patient CASPR2-Abs. Related to Figure 2

(A&B) Scatter plots showing the G ratio plotted against axon diameter of individual axons from sural nerve taken from mice treated with either Patient 1 IgG (A) or Patient 2 IgG (B) versus control IgG, n=3, > 250 axons counts per group.

(C-F) on average no difference was seen for G ratios (C&E) or the axon diameter (D&F) between groups treated with either patient or control IgG, n=3 mice per group.

(G) Representative EM images of sural nerve from mice treated with Patient 1, Patient 2 or control IgG. Scale bar 10µm.

(H and K) Quantification of the number of bilateral CASPR positive nodes between mice treated with patient or control IgG. Data shown as number of nodes per 1000μm^2 area.

(I and L) Quantification of the number of CASPR2 positive nodes as a percentage of nodes with CASPR staining between treatment groups.

(J and M) Quantification of the number of Kv1.1 positive nodes as a percentage of nodes with CASPR staining between treatment groups.

(N) High power representative images of single nodes as marked by CASPR (red) from the mouse sciatic nerve. Kv1.1 (green, top) and CASPR2 (green, bottom) staining is reduced in mice treated with patient 1 IgG. Scale bar 10µm

(O) Quantification shows a significant reduction in the area of Kv1.1 immunostaining in the patient 1 IgG group versus control.

(H-J and O) n=5 animals, (K-M) n=4 animals. 3-6 images per animal.

For O Students t test , *p<0.05 versus control IgG group. All data shown as mean ± SEM

Supplementary Figure 5 CASPR2 expression in DRG neurons. Related to Figure 3

(A) Light microscope image of ISH for CASPR2 mRNA in WT L4 mouse DRG. Scale bar 100µm.
(B) Correlation between the intensity of CASPR2 mRNA signal versus DRG neuron cell size. Data obtained from L4 and 5 DRG from WT mice (n=3). A total of 1437 cells were analysed. Individual data points shown.
(C) Combined immunofluorescence with ISH for DRG neuron markers (IB4, CGRP, NF200 and TH) and CASPR2 mRNA.
(D) Average intensity of CASPR2 mRNA signal in different populations of DRG neurons from WT mouse L4 and 5 DRG (3-4 images per animal, n=3). Data shown as mean ±SEM

Supplementary Figure 6 No gross anatomical changes found within the sensory nervous system of *Cntnap2*$^{-/-}$ mice. Related to Figure 3

(A) Representative images of spinal cord from both *Cntnap2*$^{+/+}$ and *Cntnap2*$^{-/-}$ mice taken ipsilateral to formalin (5%) injection into the hind paw stained with the neuronal activity marker c-fos (red). IB4 binding shown in blue. Scale bar 50µm.

(B) Quantification shows a clear increase in the number of c-fos positive cells in the ipsilateral dorsal horn versus contralateral following formalin injection. However no difference was seen between genotypes. Data shown as the average of 5 images per animal, *Cntnap2*$^{+/+}$ (n=5) and *Cntnap2*$^{-/-}$ (n=6).

(C) Representative images of DRG from both *Cntnap2*$^{+/+}$ and *Cntnap2*$^{-/-}$ mice, immunostained for the sensory neuronal markers IB4 (blue), CGRP (red) and NF200 (green). Scale bar 50µm

(D) Quantification of sensory neurons expressing IB4, CGRP and NF200. No difference was seen between genotypes. Data shown as the average of 2-4 images per animal, *Cntnap2*$^{+/+}$ (n=4) and *Cntnap2*$^{-/-}$ (n=5).

(E) Representative images of mouse glabrous skin immunostained for PGP9.5 to mark free nerve endings in the epidermis. Scale bar 25µm

(F) Quantification of IEDNF showed no differences between genotypes (3 images per animal, n=6 for both genotypes).

(G) Representative image of a WT dorsal horn of the spinal cord showing immunostaining for Pax2 (green), binding of IB4 (blue), and ISH for CASPR2 mRNA (red). Scale bar 50µm.

(H) Quantification of the percentage of Pax2 positive neurons that also express CASPR2 mRNA from different lamina within the dorsal horn of the spinal cord of WT mice (3-5 images per animal, n=3).

(I) Image from Glycine transporter 2-GFP reporter mice showing CASPR2 mRNA (red) expression in inhibitory interneurons in deep dorsal horn. IB4 binding shown in blue. Scale bar 50µm.

(J) Representative images of the dorsal horn of the mouse spinal cord. Immunostaining for the pan-neuronal marker NeuN (purple) and the inhibitory interneuron marker Pax2 (green) is shown. Scale bar 100µm

(K) Quantification of the percentage of Pax2 immunoreactive neurons shows no difference between genotypes, n=4.

(L and M) Quantification of gephyrin (a postsynaptic marker of inhibitory synapses, L) or VGAT (presynaptic marker of inhibitory synapses, M) positive puncta in *Cntnap2*$^{+/+}$ and *Cntnap2*$^{-/-}$ mice (n=4).

All data shown as mean ±SEM

Supplementary Figure 7 Expression of FL- and SH-CASPR2 mRNA in mouse DRG and overexpression *in vitro*. Related to Figure 5

(A) Representative image of Kv1.2 expression (green) in cultured WT DRG neurons after 1 and 5 days in vitro (DIV). NF200 in red, DAPI in blue. Scale bar 25µm.

(B) qPCR showing that CASPR2 mRNA expression is significantly reduced in mouse DRG neurons after 5 DIV, n=3 independent cultures.

(C) qPCR showing that the FL-CASPR2 is lost in DRG taken from *Cntnap2*$^{-/-}$. The mRNA for SH-CASPR2 is still expressed, n=4 per genotype.

(D) In lumbar DRG from WT mice, the mRNA of FL-CASPR2 is more greatly expressed (~10 times) than the shorter isoform, n=4.

(E) Representative image showing membrane localisation of the FL-CASPR2 tagged with eGFP in cultured mouse DRG neurons.

(F) Representative images of HEK cells transfected with plasmids containing either FL-CASPR2 or SH-CASPR2 tagged with eGFP. Membrane localisation of eGFP seen in the HEK cells transfected with FL-CASPR2, but not the shorter isoform. Scale bar 25µm.

For B and C Students t test, *p<0.05, ***p<0.001 versus either DIV0 (B) or *Cntnap2*$^{+/+}$ group (C). All data shown as mean ±SEM.

Supplementary Figure 8 Patient CASPR2-Abs bind WT mouse DRG neurons, but do not bind to the short CASPR2 isoform or DRG neurons from *Cntnap2*$^{-/-}$ mice. Related to Figure 5 and 8

(A-C) CBA using HEK cells overexpressing either FL-CASPR2 or SH-CASPR2 tagged to eGFP. Immunoreactivity for human IgG (red) shown only on cells overexpressing FL-CASPR2, but not SH-CASPR2 when treated with both patient 1 and patient 2 plasma (A and B). No human IgG immunostaining was seen in HEK cells overexpressing either isoform of CASPR2 for the healthy control plasma (C). Scale bar 50µm.

(D and E) Cultured mouse WT DRG neurons show membrane immunostaining for human IgG (green) following treatment of live cells with both Patient 1 (A) and Patient 2 (B) plasma. These cells were mainly NF200 positive (red).

(F) No binding of IgG from healthy control plasma on DRG neurons

(G) Patient IgG from plasma does not bind to cultured DRG neurons from *Cntnap2*$^{-/-}$ mice. Scale bar 25µm.

Supplementary Figure 9 Genetic ablation of FL-CASPR2 causes D-hair hyperexcitability at slow velocities with no anatomical changes at the lanceolate endings. Related to Figure 6

(A) The stimulus response curves of both *Cntnap2*$^{+/+}$ and *Cntnap2*$^{-/-}$ D-hairs to a large range of increasing stimulus velocities (*Cntnap2*$^{+/+}$ n=14, *Cntnap2*$^{-/-}$ n=20 units recorded from 16 *Cntnap2*$^{+/+}$ and 15 *Cntnap2*$^{-/-}$ mice).

(B&C) Ramp firing frequency was significantly increased at stimulation velocities of 75µm/s (B) and 150µm/s (C) (where each point is a single recording), indicating that D-hairs show significant hyperexcitability compared to *Cntnap2*$^{+/+}$ controls.

(D&E) Using whole-mount IHC, TrkB+ Lanceolate endings were visualised and quantified in both hind paw glabrous (D) and hind paw hairy (E) skin. There was no differences in the number of TrkB+ D-hairs in *Cntnap2*$^{-/-}$ or *Cntnap2*$^{+/+}$ mice in either skin type. White dashed lines denote hair follicle orientation, scale bar 20µm, (*Cntnap2*$^{-/-}$ n=3 animals, (D) 87 TrkB+ Lanceolate endings, (E) 197 TrkB+ Lanceolate endings: *Cntnap2*$^{+/+}$ n=3 animals, (D) 40 TrkB+ Lanceolate endings, (E) 220 TrkB+ Lanceolate endings). RP=running pad.

For B and C students t-test*p<0.05, **p<0.01 versus *Cntnap2*$^{+/+}$. All data shown as mean ±SEM.

Supplementary Figure 10 No change in spontaneous or capsaicin evoked EPSCs in dorsal horn neurons from *Cntnap2*$^{-/-}$ mice. Related to Figure 7

(A) Representative voltage-clamp traces showing spontaneous and capsaicin (1µM) evoked EPSCs in lamina II dorsal horn neurons from *Cntnap2*$^{-/-}$ (top) and control mice (bottom).
(B) Quantification of the frequency of EPSCs recorded for 3 minutes at baseline and during capsaicin application. A clear increase in EPSC frequency is seen in both *Cntnap2*$^{-/-}$ and *Cntnap2*$^{+/+}$ mice following capsaicin treatment. However, there was no difference in the frequency of capsaicin-evoked EPSCs between genotypes.
(C) Quantification of the amplitude of EPSCs recorded at baseline and during capsaicin application. There was no difference in the amplitude of spontaneous or capsaicin-evoked EPSCs between genotypes.
Data was taken from 6 *Cntnap2*$^{-/-}$ (n=15 cells) and 7 *Cntnap2*$^{+/+}$ mice (n=16 cells). All data shown as the mean±SEM.

Table S1 qPCR analysis of gene expression in sciatic nerve and DRG from control versus Patient IgG treated mice. Related to Figure 2

Gene name	Sciatic nerve		DRG	
	**Control IgG**	**Patient IgG**	**Control IgG**	**Patient IgG**
Ccl2^	1.0±0.25	0.54±0.08	1.0±0.14	0.99±0.06
Ifnb1	1.0±0.19	0.60±0.20	ND	ND
Il18	1.0±0.37	0.92±0.35	1.0±0.21	0.97±0.24
Il1B	ND	ND	ND	ND
Il6	1.0±0.73	0.87±0.52	1.0±0.53	1.56±0.58
Tnf	ND	ND	ND	ND

Samples were pooled from patient 1 and 2 IgG treated mice and controls to reduce variability. No significant differences were seen in the expression of cytokines and chemokines between treatment groups, n=6 for sciatic nerve control IgG, n=7 for all other groups. ND indicates that the transcript was not detected. Expression was normalised to control values and the fold change is shown for the Patient IgG group. ^qPCR performed using SYBR green. All other qPCR performed using Taqman probes. All data shown as the mean±SEM.

Table S2. EM analysis of sural nerve in mice treated with either healthy control or patient IgG. Related to Figure 2

Parameter	Control IgG	Patient 1 IgG	Control IgG	Patient 2 IgG
No. of myelinated fibres x1000/mm^2	38.9±4.4	40.6±3.2	33.9±6.3	39.0±11.1
No. of unmyelinated fibres x1000/mm^2	152.4±44.4	139±14.7	172.7±27.4	104.4±16.6
No. of remak bundles x1000/mm^2	14.5±3.9	13.6±1.4	12.1±2.0	8.1±0.05
Average no. of C-fibres/remak bundle	10.3±0.5	10.2±0.3	13.6±0.5	12.3±2.0
Schwann cell nuclei x1000/mm^2	1.1±0.03	0.9±0.45	1.7±0.2	1.6±0.1

3 animals used per group. No significant difference between treatment groups. All data shown as mean±SEM.

Table S3 Locomotor activity in *Cntnap2*$^{-/-}$ mice. Related to Figure 3

Behavioural test	*Cntnap2*$^{+/+}$	No. of mice	*Cntnap2*$^{-/-}$	No. of mice
Beam test – Percentage of correct steps	87.8±2.6%	6	83.4±3.6%	4
Accelerating RotaRod (4-32rpm)	179.2±12.3	13	216.9±27.4	8
Constant speed RotaRod (28rpm)	53.2±12.1	13	138.2±39.6*	8
Open field – No. of grid boxes entered in 3 minute period	131.7±14.3	13	148.7±17.4	8

Student's t test *p<0.05 versus *Cntnap2*$^{+/+}$ group. All data shown as mean±SEM.

Table S4 Expression levels of pain-related genes are not changed in the DRG of *Cntnap2*$^{-/-}$ mice. Related to Figure 3

Gene	*Cntnap2*$^{+/+}$	*Cntnap2*$^{-/-}$
Atf3^	1.0±0.08	1.0±0.1
Bdnf	1.0±0.05	0.83±0.07
Cana2d1	1.0±0.04	1.0±0.06
Cntn2^	1.0±0.07	1.2±0.05
Hcn2	1.0±0.11	1.1±0.07
Kcna1^	1.0±0.13	1.4±0.15
Kcna2^	1.0±0.07	1.2±0.17
Kcns1	1.0±0.06	0.96±0.02
Ngf	1.0±0.09	1.0±0.08
Ngfr	1.0±0.1	0.97±0.06
Ntrk1	1.0±0.04	0.98±0.07
Ntrk2	1.0±0.05	0.99±0.06
Ntrk3	1.0±0.03	1.0±0.03
Oprm1	1.0±0.03	0.91±0.03
Pdyn	1.0±0.1	1.2±0.08
Ptgs2	1.0±0.4	0.52±0.09
P2rx3	1.0±0.1	1.0±0.09
Scn3a	1.0±0.3	0.86±0.12
Scn9a	1.0±0.08	0.92±0.03
Scn10a	1.0±0.04	0.95±0.02
Scn11a	1.0±0.07	0.97±0.05
Trpa1	1.0±0.03	0.96±0.08
Trpm8^	1.0±0.1	0.96±0.08
Trpv1	1.0±0.08	0.89±0.07
VGF	1.0±0.1	1.0±0.4

Relative gene expression from mouse Lumbar DRG, n=4 per genotype. No statistically significant differences found between genotypes, students t test. ^qPCR performed using SYBR green. All other qPCR performed using Taqman probes. All data shown as mean±SEM.

Table S5 Biophysical properties in cultured DRG neurons from *Cntnap2*$^{-/-}$ are normal. Related to Figure 5

	Small (<25µm)		Medium (25-35 µm)		Large (>35 µm)	
	+/+	*-/-*	*+/+*	*-/-*	*+/+*	*-/-*
**R_{Input} (MΩ)**	327.11±11.43	335.42±16.01	98.33±10.32	96.76±10.34	57.89±17.90	53.22±13.87
**RMP (mV)**	-49.74±1.73	-48.97±1.81	-61.87±0.85	-59.19±1.45	-61.93±2.16	-63.83±0.70
**Capacitance (pF)**	23.39±0.93	21.87±1.35	31.75±1.18	31.10±2.22	63.50±4.59	70.23±5.25
**No. of cells**	47	49	20	20	12	13

No statistical difference found between genotypes. All data shown as mean±SEM

Table S6. Stimulus response and mechanical latency in primary afferent fibres in *Cntnap2*$^{+/+}$ and *Cntnap2*$^{-/-}$ mice. Related to Figure 6

		RAM		SAM		D-Hair		A-M		C-M		C-MH	
Cntnap2		+/+	-/-	+/+	-/-	+/+	-/-	+/+	-/-	+/+	-/-	+/+	-/-
n		23	19	14	22	14	20	25	20	20	19	15	13
Conduction velocity (m/s)		13.91 ±0.54	13.88 ±0.59	14.02 ±0.75	14.19 ±0.55	5.66 ±0.73	7.07 ±0.41	5.15 ±0.47	5.49 ±0.44	0.86 ±0.05	0.73 ±0.04	0.51 ±0.02	0.43 ±0.11
Mechanical threshold (mN)		10.08 ±2.79	10.40 ±2.26	12.22 ±1.72	9.76 ±2.27	1.31 ±0.40	0.67 ±0.20	49.91 ±2.70	49.20 ±2.93	66.49 ±8.97	70.72 ±12.49	114.0 ±27.58	109.60 ±21.97
Stimulus-Response Velocity	µm/s	Firing frequency (Hz)											
	75	5.45 ±1.94	3.03 ±0.80	14.50 ±3.41	14.50 ±2.48	6.26 ±1.04	12.91 ±1.77						
	150	10.65 ±2.65	8.04 ±1.76	16.59 ±4.40	19.28 ±3.68	17.28 ±1.78	26.45 ±2.73						
	450	27.93 ±4.45	20.09 ±3.33	29.85 ±6.54	34.59 ±5.40	50.33 ±5.40	59.49 ±4.56						
	1,500	74.61 ±9.39	59.17 ±8.64	67.09 ±11.27	69.47 ±9.23	125.76 ±13.41	132.41 ±8.83						
	15,000	264.24 ±23.20	261.92 ±20.76	209.74 ±18.56	192.34 ±16.93	270.86 ±34.35	218.89 ±25.75						
Stimulus-Response Force	mN	Firing frequency (Hz)											
	14			3.33 ±1.32	3.92 ±1.38			2.24 ±0.79	1.67 ±0.79	4.45 ±0.93	4.10 ±0.70	1.61 ±0.60	2.62 ±0.75
	49			9.73 ±3.50	11.18 ±3.69			7.16 ±2.083	5.54 ±2.18	10.05 ±1.83	9.45 ±1.47	4.43 ±1.54	5.70 ±1.36
	96			13.42 ±3.69	12.74 ±2.55			12.15 ±2.20	9.45 ±2.03	13.81 ±2.29	15.44 ±2.56	7.14 ±2.07	7.14 ±1.43
	190			20.17 ±4.52	13.70 ±2.29			21.20 ±2.40	19.72 ±2.78	20.57 ±2.60	26.31 ±3.41	9.96 ±2.10	9.93 ±1.93
	292			13.56 ±3.53	10.98 ±1.77			26.00 ±2.75	23.04 ±2.03	22.36 ±3.44	25.15 ±3.02	11.35 ±2.61	9.38 ±2.10
		µm/s											
Mechanical latency	Stim 1	475.70 ±95.56	461.70 ±90.56	413.77 ±43.84	332.63 ±39.91	161.30 ±42.13	95.87 ±37.54	92.61 ±74.25	76.56 ±62.61	115.48 ±62.88	116.24 ±57.16	186.08 ±61.92	357.15 ±154.36
	Stim 2	224.82 ±39.46	205.38 ±40.18	235.06 ±23.69	223.85 ±29.78	73.70 ±19.14	52.00 ±15.61	76.40 ±58.14	18.74 ±3.45	40.08 ±12.00	60.58 ±36.56	115.31 ±77.30	69.52 ±18.02
	Stim 3	60.10 ±10.71	65.80 ±9.65	75.83 ±6.33	63.84 ±8.98	13.93 ±3.22	15.38 ±5.01	30.43 ±9.25	22.56 ±4.60	21.99 ±3.03	22.10 ±2.20	131.40 ±64.65	38.18 ±6.61
	Stim 4	18.00 ±2.92	18.35 ±2.31	22.85 ±1.98	23.53 ±2.72	5.39 ±1.10	6.74 ±1.32	27.73 ±3.60	25.27 ±4.75	26.27 ±5.03	24.63 ±2.20	80.66 ±36.34	39.05 ±6.16
	Stim 5	3.22 ±0.23	3.35 ±0.21	3.76 ±0.24	4.71 ±0.50	2.08 ±0.23	2.75 ±0.27	28.92 ±3.35	28.00 ±4.85	32.26 ±4.31	37.28 ±4.64	60.17 ±11.19	42.54 ±6.50

Data taken from 16 *Cntnap2*$^{+/+}$ and 15 *Cntnap2*$^{-/-}$ mice. Note: adaptation properties were unchanged between genotypes for AMs and SAMs during the Hold phase of the Ramp hold protocol at 190mN and 292mN. All data shown as mean±SEM.

Table S7. Primary and Secondary antibodies and fluorescent dyes used for histology. Related to STAR methods.

Antigen	Company	Host/Fluorophore	Dilution
ATF3	Santa Cruz	Rabbit	1:500
CGRP	Enzo Life Sciences	Sheep	1:400
CGRP	Peninsula Laboratories	Rabbit	1:1000
IB4 target	Sigma	Conjugated to biotin	1:100
PGP9.5	Ultraclone	Rabbit	1:1000
NF200	Sigma	Mouse	1:250
NF200	Abcam	Chicken	1:10000
CASPR2	Abcam	Rabbit	1:100
CASPR	Gift – Bhat MA	Guinea pig	1:1500
IBA1	Wako	Rabbit	1:1000
Kv1.1	Gift – Trimmer J	Rabbit	1:200
Kv1.2	UC Davis/NIH NeuroMab Facility	Mouse	1:100
Beta-III-tubulin	Sigma	Mouse	1:1000
Pax2	Thermo Fisher Scientific	Rabbit	1:200
NeuN	Abcam	Rabbit	1:1000
NeuN	Millipore	Chicken	1:500
TrkB	R&D Systems	Goat	1:250
Tyrsosine hydroxylase	Millipore	Sheep	1:200
Gephyrin	Frontier Institute	Rabbit	1:200
Vgat	Frontier Institute	Goat	1:200
c-fos	Santa Cruz	Rabbit	1:500
CD3	Abcam	Rabbit	1:500
CD68	BioRad	Rat	1:400
Ly6G	R&D Systems	Rat	1:100
GFAP	Dako	Rabbit	1:500
GFP	Abcam	Chicken	1:1000
Rabbit IgG	Thermo Fisher Scientific	Alexa 488	1:1000
Rabbit IgG	Jackson ImmunoResearch	Cy3	1:500

	Labs		
Rabbit IgG	Thermo Fisher Scientific	Pacific Blue	1:500
Rabbit IgG	Thermo Fisher Scientific	546	1:500
Sheep IgG	Jackson ImmunoResearch Labs	Cy3	1:500
Streptavidin	Thermo Fisher Scientific	Alexa 405	1:500 and 1:100
Streptavidin	Thermo Fisher Scientific	Pacific blue	1:500
Mouse IgG	Thermo Fisher Scientific	Alexa 488	1:1000
Mouse IgG	Thermo Fisher Scientific	Pacific Blue	1:500
Guinea pig IgG	Thermo Fisher Scientific	Alexa 568	1:500
Human IgG	Thermo Fisher Scientific	Alex 488	1:1000
Human IgG	Thermo Fisher Scientific	Alex 546	1:1000
Nissl	Thermo Fisher Scientific	NeuroTrace 530/615	1:100
Sheep IgG	Thermo Fisher Scientific	Alexa 488	1:1000
Rat IgG	Thermo Fisher Scientific	Alexa 488	1:500
Rat IgG	Thermo Fisher Scientific	Alexa 546	1:500
Chicken IgG	Thermo Fisher Scientific	Alexa 488	1:1000
Chicken IgG	Thermo Fisher Scientific	Alexa 546	1:1000

Table S8. Sequences or assay IDs for primers used in qPCR. Related to STAR methods.

Gene name	Forward primer (5'-3')	Reverse primer (5'-3')	Assay ID
Trpm8	CAGCCTGGGGTCCAGAATTT	AATGATACGAGGCCACAGCC	-
Aft3	GTGCCTGCAGAAAGAGTCAGA	GAGGTTCCTCTCGTCTTCCG	-
FL-Cntnap2	GCTCCCTCACCACTGACTTC	GCAAGTTTCCTGTGGGCTTG	-
SH-Cntnap2	ATTCCAGCATCTGGCGACAA	CCTTGGGATGCAGGTGGATT	-
Kcna1	TTACCCTGGGCACGGAGATA	ACACCCTTACCAAGCGGATG	-
Kcna2	CATCTGCAAGGGCAACGTCAC	CCTTTGGAAGGAAGGAGGCA	-
Cntn2	CCCAGTATCAAAGCCCGGTT	ACAGGGTTCCCAAAGGCAAA	-
Ccl2	CTGTAGTTTTTGTCACCAAGCTCA	GTGCTGAAGACCTTAGGGCA	
18s	GGACCAGAGCGAAAGCATTTG	GCCAGTCGGCATCGTTTATG	-
Hprt1	GTCCTGTGGCCATCTGCCTAG	TGGGGACGCAGCAACTGACA	-
Gapdh	TGTGTCCGTCGTGGATCTGA	TTGCTGTTGAAGTCGCAGGAG	-
Hcn2	-	-	Mm00468538_m1
Kcns1	-	-	Mm00492824_m1
P2rx3	-	-	Mm00523699_m1
Scn3a	-	-	Mm00658167_m1
Scn9a	-	-	Mm00450762_s1
Scn10a	-	-	Mm00501467_m1
Scn11a	-	-	Mm00449377_m1
Trpa1	-	-	Mm01227437_m1
Trpv1	-	-	Mm01246302_m1
Cacna2d1	-	-	Mm00486607_m1
Bdnf	-	-	Mm04230607_s1
Ngf	-	-	Mm00443039_m1
Ngfr	-		Mm01309635_m1
Ntrk1	-	-	Mm01219406_m1
Ntrk2	-	-	Mm00435422_m1
Ntrk3	-	-	Mm00456222_m1
Vgf	-	-	Mm01204485_s1
Oprm1	-	-	Mm00440568_m1
Pdyn	-	-	Mm00457573_m1
Ptgs2	-	-	Mm00478374_m1
Ifnb1			Mm00439552_s1
Il18			Mm00434225_m1
Il1B			Mm01336189_m1
Il6			Mm99999064_m1
Tnf			Mm99999068_m1
18s	-	-	Hs99999901_s1
Hprt1	-	-	Mm00446968_m1
Gapdh	-	-	Mm99999915_g1

Data S1 Patient 2 case history. Related to Figure 1&2

A 71 year old man presented with a 2 year history of burning pain in the feet; when walking. He described the sensation as if walking on glass. He subsequently developed paraesthesia of the feet as well as paraestheia and clumsiness of the hands. He denied muscle weakness but had noted that his balance had deteriorated. He had developed a dry mouth and impotence but denied any other autonomic symptoms. He had noted that his speech had become slurred and at times noted difficulty swallowing. He complained of cognitive symptoms and in particular short term memory loss. He also developed new episodes of brief alterations in consciousness which lasted 10 seconds, were associated with a sensation he described as 'goosebumps' and reduced awareness of his surroundings. These occurred up to 10 times a day and were thought to potentially represent complex partial seizures. He had a past medical history of type II diabetes mellitus diagnosed 5 years ago which was well controlled on metformin 500mg TDS with no diabetic retinopathy on retinal screening and no micro-albuminuria. His pain had not responded to amitriptyline but he reported some improvement on Gabapentin 600mg TDS. On pain screening questionnaires he scored 4 on the DN4 and 25 on PainDETECT consistent with a diagnosis of neuropathic pain. The pain severity score on the Brief Pain Inventory score was 6.83 and pain interference score was 4.25. On a body map he labelled the pain as affecting his hands and feet.

On clinical examination blood pressure was 120 mmHg systolic and 62 mmHg diastolic with no postural drop. Eye movements were normal. He had a mild dysarthria and broad based gait with a positive Romberg's sign. There was no muscle wasting, power was normal throughout. All deep tendon reflexes were preserved other than the ankle jerks which were absent. On sensory examination pin prick, light touch and proprioception were normal. Vibration sense was impaired in the toes. Thermal thresholds (cool detection, warm detection and thermal sensory limen) were normal.

Nerve conduction studies showed normal sensory and motor conduction and on EMG there was no evidence of neuromyotonia. A skin biopsy was taken from 10cm above the left lateral malleolus and intra-epidermal nerve fibre density was normal relative to age and gender matched controls (4.7 fibres/mm). MRI of the brain and spinal cord was normal with no evidence of cerebellar atrophy and no features suggestive of limbic encephalitis. A barium swallow demonstrated laryngeal penetration of liquids and occasionally solids.

A Dendrotoxin assay for anti-voltage gated potassium channel complex antibodies was strongly positive (2689 pmol/l), with the presence of antibodies directed against CASPR2 but not LGI1. The patient was treated with prednisolone and 5 plasma exchanges. The titre of anti-voltage gated potassium channel complex antibody dropped to 602pmol/l (CASPR2 antibodies remained positive). The episodes of altered consciousness resolved and his balance improved. There was also some improvement in the burning pain and paraesthesia although these symptoms did not completely resolve. He was managed with ongoing immunosuppression with mycophenolate 1g BD.
